# Supplementary material for: Self assembling nanoparticle enzyme clusters provide access to substrate channeling in multienzymatic cascades
Source: Nat Commun. 2023 Mar 30;14:1757. doi: 10.1038/s41467-023-37255-9 (PMC10060375; doi:10.1038/s41467-023-37255-9)
Supplement: Supplementary file 1 — Supplementary Information [file 41467_2023_37255_MOESM1_ESM.pdf]

SUPPLEMENTARY INFORMATION

Self Assembling Nanoparticle Enzyme Clusters Provide Access to Substrate Channeling in Multienzymatic Cascades

Joyce C. Breger,[1,*] James N. Vranish,[1,2,*] Eunkeu Oh,[3] Michael H. Stewart,[3] Kimihiro Susumu,[3] Guillermo Lasarte Aragonés,[1,4] Gregory A. Ellis,[1] Scott A. Walper,[1] Sebastián A. Díaz,[1] Shelby L. Hooe,[1,5] William P. Klein,[1,5] Meghna Thakur,[1,4] Mario G. Ancona,[6,7] and Igor L. Medintz[1**]

[1]Center for Bio/Molecular Science and Engineering, Code 6900
U.S. Naval Research Laboratory
Washington, D.C. 20375, USA

[2]Department of Chemistry, Engineering, and Physics
Franciscan University of Steubenville
Steubenville, OH 43952, USA

[3]Optical Sciences Division, Code 5611
U.S. Naval Research Laboratory
Washington, D.C. 20375, USA

[4]College of Science
George Mason University
Fairfax, VA 22030, USA

[5]National Research Council
Washington, D.C. 20001, USA

[6]Electronic Science and Technology Division, Code 6800
U.S. Naval Research Laboratory
Washington, D.C. 20375, USA

[7]Department of Electrical and Computer Engineering
Florida State University
Tallahassee, FL 32310, USA

[*]These authors contributed equally
[**]Email: igor.medintz@nrl.navy.mil

Keywords: Substrate channeling, enzyme, nanoparticle, quantum dot, 2-D nanoplatelet, synthetic biology, biocatalysis, glycolysis, diffusion, cascade.

Supplementary Information Table of Contents

Section	Page #
**Table of Contents**	**2**
**Supplementary Methods**	**3**
Chemical structure of the QD and AuNP ligands	3
Chemical structure of key cofactors and co-substrates	3
Enzyme sequences	4
Individual enzyme assays	7
Enzyme ratios per NP utilized	20
Kinetic simulations	22
Modeling of glycolytic cascades assembled on nanoparticles	22
Optimization of turnover	25
Possibilities of channeling	28
Thermodynamic analysis and ΔG	30
Mass spectral analysis of substrates, intermediaries, and products	36
HPLC purification of 3-PG from the 7 enzyme cascade	37
**Supplementary Data**	**38**
Physicochemical analyses of NP-enzyme cluster formation	38
Discussion of results from the physicochemical analyses	40
Geometric estimates of enzyme fitting to the NPs	41
Agarose gel mobility assays and PAGE analysis	44
TEM analysis of nanoclusters (QD ratio *versus* nanocluster size)	57
Dynamic light scattering estimating the number of QDs in clusters	63
FRET assays estimating the number of QDs in clusters	65
Estimating labeled enzyme incorporation into clusters with FRET	67
Numerical simulation of the formation of nanoparticle aggregates	71
Supplementary assay data	75
Comparison of enzyme activity when diluted on and off NP	75
Enzyme kinetics at different ratios on/off NP	77
Mass spectral analysis of substrates, intermediaries and products	89
Test of reverse direction gluconeogenic reactions starting with 3-PG	91
Assay shaking and testing different enzyme assembly orders with QDs	93
Forward, backward, and batch assembly results	94
Testing of other nanoparticle and enzyme aggregated materials	96
**Supplementary References**	**104**

Supplementary Methods

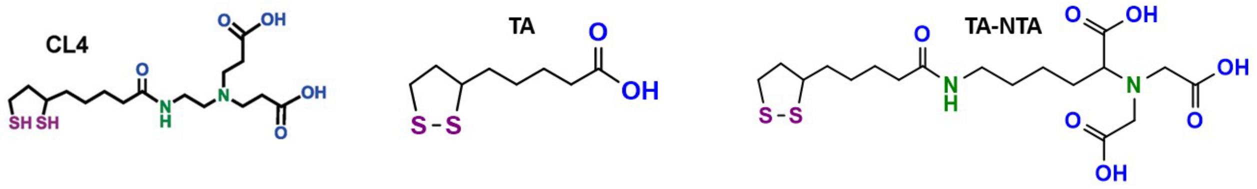

Supplementary Figure 1. Chemical structure of the QD and AuNP ligands. (Left-to-right) The compact ligand 4 (CL4) structure is shown with the dithiols in the open dithiolane configuration. Also shown are thioctic acid (TA) and thioctic acid-nitrilotriacetic acid (TA-NTA) with the dithiols in closed form.

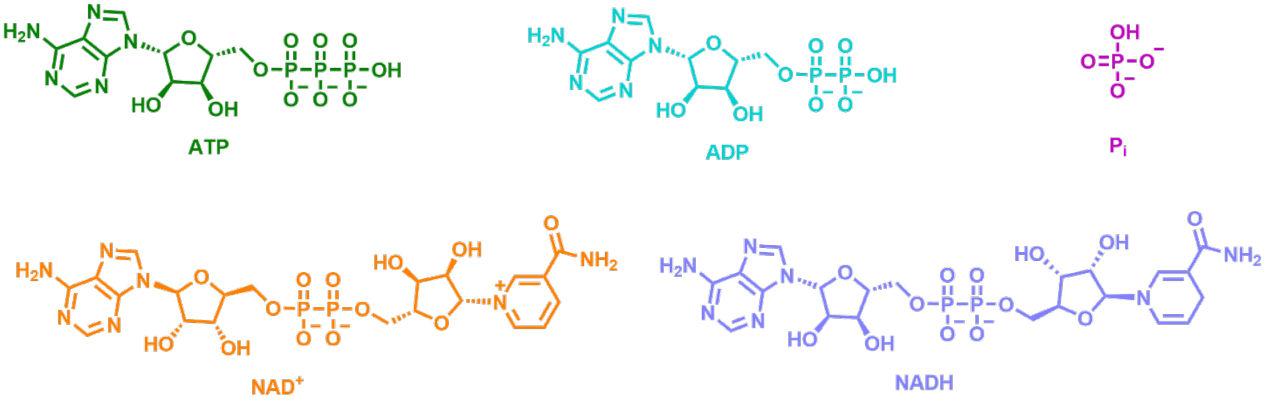

Supplementary Figure 2. Chemical structure of key cofactors and co-substrates.

Enzymes sequences (given N- to C-terminal for protein monomers).

Amylase (Amy):
MGAVNGKGMNPDYKAYLMAPLKKIPEVTNWETFENDLRWAKQNGFYAITVDFWWGD
MEKNGDQQFDFSYAQRFAQSVKNAGMKMIPIISTHQCGGNVGDDCNVPIPSWVWNQK
SDDSLYFKSETGTVNKETLNPLASDVIRKEYGELYTAFAAAMKPYKDVIAKIYLSGGPA
GELRYPSYTTSDGTGYPSRGKFQAYTEFAKSKFRLWVLNKYGSLNEVNKAWGTKLISE
LAILPPSDGEQFLMNGYLSMYGKDYLEWYQGILENHTKLIGELAHNAFDTTFQVPIGAKI
AGVHWQYNNPTIPHGAEKPAGYNDYSHLLDAFKSAKLDVTFTCLEMTDKGSYPEYSMP
KTLVQNIATLANEKGIVLNGENALSIGNEEEYKRVAEMAFNYNFAGFTLLRYQDVMYN
NSLMGKFKDLLGVTPVMQTIVVKNVPTTIGDTVYITGNRAELGSWDTKQYPIQLYYDSH
SNDWRGNVVLPAERNIEFKAFIKSKDGTVKSWQTIQQSWNPVPLKTTSHTSSWLEHHHH
HH

Maltase (Mal):
MGSSHHHHHHSSGLVPRGSHMMTISDHPETEPKWWKEATIYQIYPASFKDSNNDGWGD
LKGITSKLQYIKDLGVDAIWVCPFYDSPQQDMGYDISNYEKVWPTYGTNEDCFELIDKT
HKLGMKFITDLVINHCSTEHEWFKESRSSKTNPKRDWFFWRPPKGYDAEGKPIPPNNWK
SFFGGSAWTFDETTNEFYLRLFASRQVDLNWENEDCRRAIFESAVGFWLDHGVDGFRID
TAGLYSKRPGLPDSPIFDKTSKLQHPNWGSHNGPRIHEYHQELHRFMKNRVKDGREIMT
VGEVAHGSDNALYTSAARYEVSEVFSFTHVELGTSPFFRYNIVPFTLKQWKEAIASNFLF
INGTDSWATTYIENHDQARSITRFADDSPKYRKISGKLLTLLECSLTGTLYVYQGQEIGQI
NFKEWPIEKYEDVDVKNNYEIIKKSFGKNSKEMKDFFKGIALLSRDHSRTPMPWTKDKP
NAGFTGPDVKPWFFLNESFEQGINVEQESRDDDSVLNFWKRALQARKKYKELMIYGYD
FQFIDLDSDQIFSFTKEYEDKTLFAALNFSGEEIEFSLPREGASLSFILGNYDDTDVSSRVL
KPWEGRIYLVKLE

Invertase (Inv):
MGFNFNASRWTRAQAMKVNKFDLTTSMPEIGTDFPIMRDDLWLWDTWPLRDINGNPV
SFKGWNVIFSLVADRNIPWNDRHSHARIGYFYSKDGKSWVYGGHLLQESANTRTAEWS
GGTIMAPGSRNQVETFFTSTLFDKNGVREAVAAVTKGRIYADSEGVWFKGFDQSTDLF
QADGLFYQNYAENNLWNFRDPHVFINPEDGETYALFEANVATVRGEDDIGEDEIGPVPA
NTVVPKDANLCSASIGIARCLSPDRTEWELLPPLLTAFGVNDQMERPHVIFQNGLTYLFT
ISHDSTYADGLTGSDGLYGFVSENGIFGPYEPLNGSGLVLGGPASQPTEAYAHYIMNGL
VESFINEIIDPKSGKVIAGGSLAPTVRVELQGHETFATEVFDYGYIPASYAWPVWPFPDRR
KLEHHHHHH

Glucokinase (Glk):
MGSSHHHHHHSSGLVPRGSHMTKYALVGDVGGTNARLALCDIASGEISQAKTYSGLDY
PSLEAVIRVYLEEHKVEVKDGCIAIACPITGDWVAMTNHTWAFSIAEMKKNLGFSHLEII
NDFTAVSMAIPMLKKEHLIQFGGAEPVEGKPIAVYGAGTGLGVAHLVHVDKRWVSLPG
EGGHVDFAPNSEEEAIILEILRAEIGHVSAERVLSGPGLVNLYRAIVKADNRLPENLKPKD
ITERALADSCTDCRRALSLFCVIMGRFGGNLALNLGTFGGVFIAGGIVPRFLEFFKASGFR
AAFEDKGRFKEYVHDIPVYLIVHDNPGLLGSGAHLRQTLGHIL

Phosphoglucose isomerase (PGI):
MGSSHHHHHHSSGLVPRGSHMKNINPTQTAAWQALQKHFDEMKDVTIADLFAKDGDR
FSKFSATFDDQMLVDYSKNRITEETLAKLQDLAKECDLAGAIKSMFSGEKINRTENRAV
LHVALRNRSNTPILVDGKDVMPEVNAVLEKMKTFSEAIISGEWKGYTGKAITDVVNIGI
GGSDLGPYMVTEALRPYKNHLNMHFVSNVDGTHIAEVLKKVNPETTLFLVASKTFTTQ
ETMTNAHSARDWFLKAAGDEKHVAKHFAALSTNAKAVGEFGIDTANMFEFWDWVGG
RYSLWSAIGLSIVLSIGFDNFVELLSGAHAMDKHFSTTPAEKNLPVLLALIGIWYNNFFG
AETEAILPYDQYMHRFAAYFQQGNMESNGKYVDRNGNVVDYQTGPIIWGEPGTNGQH
AFYQLIHQGTKMVPCDFIAPAITHNPLSDHHQKLLSNFFAQTEALAFGKSREVVEQEYR
DQGKDPATLDYVVPFKVFEGNRPTNSILLREITPFSLGALIALYEHKIFTQGVILNIFTFDQ
WGVELGKQLANRILPELKDDKEISSHDSSTNGLINRYKAWRG

Phosphofructokinase I (FPK):
MGSSHHHHHHSSGLVPRGSHMVRIYTLTLAPSLDSATITPQIYPEGKLRCTAPVFEPGGG
GINVARAIAHLGGSATAIFPAGGATGEHLVSLLADENVPVATVEAKDWTRQNLHVHVE
ASGEQYRFVMPGAALNEDEFRQLEEQVLEIESGAILVISGSLPPGVKLEKLTQLISAAQKQ
GIRCIVDSSGEALSAALAIGNIELVKPNQKELSALVNRELTQPDDVRKAAQEIVNSGKAK
RVVVSLGPQGALGVDSENCIQVVPPPVKSQSTVGAGDSMVGAMTLKLAENASLEEMVR
FGVAAGSAATLNQGTRLCSHDDTQKIYAYLSR

Fructose-bisphosphate aldolase (FBA):
MGSSHHHHHHSSGLVPRGSHMSKIFDFVKPGVITGDDVQKVFQVAKENNFALPAVNCV
GTDSINAVLETAAKVKAPVIVQFSNGGASFIAGKGVKSDVPQGAAILGAISGAHHVHQM
AEHYGVPVILHTDHCAKKLLPWIDGLLDAGEKHFAATGKPLFSSHMIDLSEESLQENIEI
CSKYLERMSKIGMTLEIELGCTGGEEDGVDNSHMDASALYTQPEDVDYAYTELSKISPR
FTIAASFGNVHGVYKPGNVVLTPTILRDSQEYVSKKHNLPHNSLNFVFHGGSGSTAQEIK
DSVSYGVVKMNIDTDTQWATWEGVLNYYKANEAYLQGQLGNPKGEDQPNKKYYDPR
VWLRAGQTSMIARLEKAFQELNAIDVL

Triose phosphate isomerase (TPI):
MGSSHHHHHHSSGLVPRGSHMRHPLVMGNWKLNGSRHMVHELVSNLRKELAGVAGC
AVAIAPPEMYIDMAKREAEGSHIMLGAQNVDLNLSGAFTGETSAAMLKDIGAQYIIIGHS
ERRTYHKESDELIAKKFAVLKEQGLTPVLCIGETEAENEAGKTEEVCARQIDAVLKTQG
AAAFEGAVIAYEPVWAIGTGKSATPAQAQAVHKFIRDHIAKVDANIAEQVIIQYGGSVN
ASNAAELFAQPDIDGALVGGASLKADAFAVIVKAAEAAKQA

Glyceraldehyde-3-phosphate dehydrogenase (GPD):
MGSSHHHHHHSSGLVPRGSHMTIKVGINGFGRIGRIVFRAAQKRSDIEIVAINDLLDADY
MAYMLKYDSTHGRFDGTVEVKDGHLIVNGKKIRVTAERDPANLKWDEVGVDVVAEA
TGLFLTDETARKHITAGAKKVVMTGPSKDNTPMFVKGANFDKYAGQDIVSNASCTTNC
LAPLAKVINDNFGIIEGLMTTVHATTATQKTVDGPSHKDWRGGRGASQNIIPSSTGAAK
AVGKVLPELNGKLTGMAFRVPTPNVSVVDLTVRLEKAATYEQIKAAVKAAAEGEMKG
VLGYTEDDVVSTDFNGEVCTSVFDAKAGIALNDNFVKLVSWYDNETGYSNKVLDLIAH
ISK

Phosphoglycerate kinase (PGK):
MGSSHHHHHHSSGLVPRGSHMSVIKMTDLDLAGKRVFIRADLNVPVKDGKVTSDARIR
ASLPTIELALKQGAKVMVTSHLGRPTEGEYNEEFSLLPVVNYLKDKLSNPVRLVKDYLD
GVDVAEGELVVLENVRFNKGEKKDDETLSKKYAALCDVFVMDAFGTAHRAQASTHGI
GKFADVACAGPLLAAELDALGKALKEPARPMVAIVGGSKVSTKLTVLDSLSKIADQLIV
GGGIANTFIAAQGHDVGKSLYEADLVDEAKRLLTTCNIPVPSDVRVATEFSETAPATLKS
VNDVKADEQILDIGDASAQELAEILKNAKTILWNGPVGVFEFPNFRKGTEIVANAIADSE
AFSIAGGGDTLAAIDLFGIADKISYISTGGGAFLEFVEGKVLPAVAMLEERAKK

Phosphoglycerate mutase (PGM):
MGSSHHHHHHSSGLVPRGSHMSVSKKPMVLVILDGYGYREEQQDNAIFSAKTPVMDAL
WANRPHTLIDASGLEVGLPDRQMGNSEVGHVNLGAGRIVYQDLTRLDVEIKDRAFFAN
PVLTGAVDKAKNAGKAVHIMGLLSAGGVHSHEDHIMAMVELAAERGAEKIYLHAFLD
GRDTPPRSAESSLKKFEEKFAALGKGRVASIIGRYYAMDRDNRWDRVEKAYDLLTLAQ
GEFQADTAVAGLQAAYARDENDEFVKATVIRAEGQPDAAMEDGDALIFMNFRADRAR
EITRAFVNADFDGFARKKVVNVDFVMLTEYAADIKTAVAYPPASLVNTFGEWMAKND
KTQLRISETEKYAHVTFFFNGGVEESFKGEDRILINSPKVATYDLQPEMSSAELTEKLVA
AIKSGKYDTIICNYPNGDMVGHTGVMEAAVKAVEALDHCVEEVAKAVESVGGQLLITA
DHGNAEQMRDPATGQAHTAHTNLPVPLIYVGDKNVKAVAGGKLSDIAPTMLSLMGME
IPQEMTGKPLFIVE

Enolase (Eno):
MGSSHHHHHHSSGLVPRGSHMSKIVKIIGREIIDSRGNPTVEAEVHLEGGFVGMAAAPSG
ASTGSREALELRDGDKSRFLGKGVTKAVAAVNGPIAQALIGKDAKDQAGIDKIMIDLDG
TENKSKFGANAILAVSLANAKAAAAAKGMPLYEHIAELNGTPGKYSMPVPMMNIINGG
EHADNNVDIQEFMIQPVGAKTVKEAIRMGSEVFHHLAKVLKAKGMNTAVGDEGGYAP
NLGSNAEALAVIAEAVKAAGYELGKDITLAMDCAASEFYKDGKYVLAGEGNKAFTSEE
FTHFLEELTKQYPIVSIEDGLDESDWDGFAYQTKVLGDKIQLVGDDLFVTNTKILKEGIE
KGIANSILIKFNQIGSLTETLAAIKMAKDAGYTAVISHRSGETEDATIADLAVGTAAGQIK
TGSMSRSDRVAKYNQLIRIEEALGEKAPYNGRKEIKGQA

Pyruvate kinase II (PykA):
MGSSHHHHHHSSGLVPRGSHMSRRLRRTKIVTTLGPATDRDNNLEKVIAAGANVVRMN
FSHGSPEDHKMRADKVREIAAKLGRHVAILGDLQGPKIRVSTFKEGKVFLNIGDKFLLD
ANLGKGEGDKEKVGIDYKGLPADVVPGDILLLDDGRVQLKVLEVQGMKVFTEVTVGG
PLSNNKGINKLGGGLSAEALTEKDKADIKTAALIGVDYLAVSFPRCGEDLNYARRLARD
AGCDAKIVAKVERAEAVCSQDAMDDIILASDVVMVARGDLGVEIGDPELVGIQKALIRR
ARQLNRAVITATQMMESMITNPMPTRAEVMDVANAVLDGTDAVMLSAETAAGQYPSE
TVAAMARVCLGAEKIPSINVSKHRLDVQFDNVEEAIAMSAMYAANHLKGVTAIITMTES
GRTALMTSRISSGLPIFAMSRHERTLNLTALYRGVTPVHFDSANDGVAAASEAVNLLRD
KGYLMSGDLVIVTQGDVMSTVGSTNTTRILTVE

Lactate dehydrogenase (LDH):
MGSSHHHHHHSSGLVPRGSHMKLAVYSTKQYDKKYLQQVNESFGFELEFFDFLLTEKT
AKTANGCEAVCIFVNDDGSRPVLEELKKHGVKYIALRCAGFNNVDLDAAKELGLKVVR
VPAYDPEAVAEHAIGMMMTLNRRIHRAYQRTRDANFSLEGLTGFTMYGKTAGVIGTGK

IGVAMLRILKGFGMRLLAFDPYPSAAALELGVEYVDLPTLFSESDVISLHCPLTPENYHL
LNEAAFDQMKNGVMIVNTSRGALIDSQAAIEALKNQKIGSLGMDVYENERDLFFEDKS
NDVIQDDVFRRLSACHNVLFTGHQAFLTAEALTSISQTTLQNLSNLEKGETCPNELV

Individual enzyme assays.

Amylase (Amy):

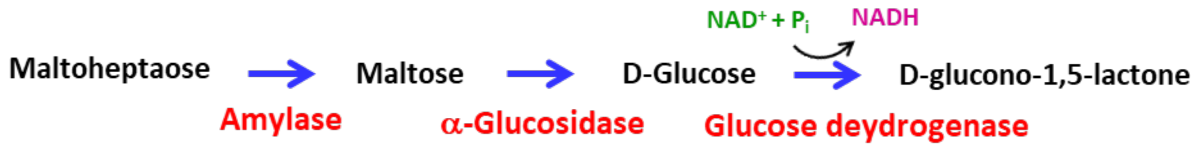

Supplementary Figure 3. Coupled enzyme format used to monitor amylase activity. Amylase concentration was fixed while all other enzymes and co-substrates were in vast excess and saturating.

NADH formation was measured as a function of time to determine the apparent kinetic parameters of amylase as displayed on QDs or free in solution at the same concentration. Stock solutions were prepared in 250 mM HEPES, pH = 7.4 and consisted of: 1 μM solution of amylase, 0.5 μM 520 QD capped with CL4, 750 U/mL α-glucosidase, or 3833 U/mL of glucose dehydrogenase. 40 mg of maltoheptaose was weighed and dissolved in 0.8 mL buffer while 54 mg of NAD trihydrate was dissolved in 1.66 mL 90 mM NaOH. To prepare the 520 QD-amylase bioconjugates 7.2 pmol of amylase was allowed to assemble with 0 to 57.6 pmol of 520 QD for at least 30 min at 4 ºC in a 1.5 mL Eppendorf tubes. Afterwards, 58 units (U) of α-glucosidase and 29 U of glucose dehydrogenase were added to each Eppendorf tube. The total volume for each tube was brought up to 1450 μL with buffer. The substrate consisted of decreasing amounts of maltoheptaose and a constant amount of NAD^+ in 250 mM HEPES, pH = 7.4. Briefly, 25 μL of sample and 25 μL of substrate was added to each well of a 384-well plate as described previously.[1] The final concentrations for the assay were 2.5 nM amylase, 20 U α-glucosidase, 10 U glucose dehydrogenase, 300 μM NAD^+, and 78 to 10,010 μM maltoheptaose. Absorbance at 340 nm was followed on a Tecan Spark plate reader utilizing a kinetic program that consisted of shaking the plate for 2 sec prior to taking a reading every 24 seconds. Absorbance values were converted to concentration values utilizing the Beer–Lambert equation (A = εbc), where $\varepsilon = 6220\ M^{-1}\ cm^{-1}$ and a path length (b) of 0.516 cm. The linear portions of the progress curves where utilized to determine the initial rates, which were then plotted *versus* maltotetrose concentration. The Michaelis-Menten equation was fitted to the resulting curves utilizing Sigma-Plot's enzyme kinetics module to determine the apparent kinetic variables. We note that in some assays maltotetrose was substituted for maltoheptaose depending upon availability of these specialty chemicals.

Maltase (Mal):

The apparent kinetic parameters of maltase on or off QDs was determined by monitoring the formation of *p*-nitrophenol from commercial 4-nitrophenyl α-D-glucopyranoside over time. Stock solutions were made in 250 mM HEPES buffer pH = 7.4 consisting of: 2.5 μM maltase, 0.5 and 5 μM QD 520 CL4, or 100 mg/mL 4-nitrophenyl α-D-glucopyranoside. Briefly, 25 pmol of maltase were allowed to assemble with 0 to 100 pmol of QD in 5 mL Eppendorf tubes at 4 ºC for at least 30 min. Afterwards, the total volume was brought up to 4.2 mL. For NPLs, 18 pmol of maltase were allowed to assemble with 0 to 72 pmol NPLs in 2 mL Eppendorf tubes at 4ºC for at least 30 min. Afterwards, the total volume was brought up to 1.5 mL with HEPES buffer. Serial dilutions ranging in concentration from 78 to 10,000 μM of 4-nitrophenyl α-D-glucopyranoside were made in 250 mM HEPES buffer. Briefly, 25 μL of sample and 25 μL of substrate was added to each well of a 384-well plate as described previously.[1] The final concentration of maltase was 3 nM when attached to QDs, 6 nM when attached to NPLs, QDs ranged in concentration from 0 to 12 nM, NPLs ranged in concentration 0 to 24 nM, while substrate ranged in concentration from 39 to 5000 μM. Absorbance at 405 nm was measured every 23 s utilizing a plate reader as described for amylase. Absorbance values were converted to concentration values utilizing a standard curve of serial dilutions of *p*-nitrophenol ($\varepsilon \sim 18\,000$ M^{-1} cm^{-1}) as described in refs.[2-5] Kinetic parameters were determined in the same manner as described for amylase.

Invertase (Inv):

Invertase (Inv) catalyzes the conversion of sucrose into fructose and glucose serving as an additional route for glucose addition into the glycolysis pathway. NADH formation was measured over time to determine the apparent kinetic parameters when invertase was free in solution or when attached to a nanoparticle such as a QD or NPL in a coupled assay format with glucokinase (Glk) and glucose-6-phosphate dehydrogenase. Stock solutions prepared in 250 mM HEPES pH=7.4 buffer consisted of 20 μM invertase and 6.2 μM 520 QD capped with CL4 or 1.6 μM NPL. To prepare the bioconjugates, 248 pmol invertase was combined with 0 to 248 pmol of NP to achieve ratios of invertase to NP of 0 to 4. The enzyme was allowed to assemble to the NP at 4 ºC on ice for at least 30 min. Following assembly, 248 pmol of Glk was added followed by 12.4 U of glucose-6-phosphate dehydrogenase, the final volume of each stock invertase-NP solution was 655 μL. Stock substrate solutions ranged in concentration from 2.24 to 288 mM sucrose containing 30 mM $MgCl_2$, 15 mM ATP, and 600 μM NAD^+ in 250 mM HEPES buffer. Briefly, 25 μL of invertase-NP bioconjugate and 25 μL of substrate was added to each well of a 384-well plate as described previously.[1] The final concentration of all components in the well are as follows: 189 nM invertase, 0 to 189 nM QD or NPL, 189 nM Glk, 9.5 U glucose-6-phosphate dehydrogenase, 15 mM $MgCl_2$, 7.5 mM ATP, 300 μM NAD^+, and sucrose ranging in concentration from 1.12 to 143.5 mM. Absorbance at 340 nm was followed on a Tecan Spark plate reader utilizing a kinetic program that consisted of shaking the plate for 5 sec prior to taking an absorbance reading for up to 16 h. Absorbance values were converted to concentration values utilizing the Beer–Lambert equation ($A = \varepsilon bc$) where $\varepsilon = 6220\ M^{-1}\ cm^{-1}$ and a path length (b) of 0.516 cm. The linear portions of the progress curves where utilized to determine the initial rates, which were then plotted *versus* sucrose concentration. The Michaelis-Menten equation was fitted to the resulting curves utilizing Sigma-Plot's enzyme kinetics module to determine the apparent kinetic variables.

Glucokinase (Glk):

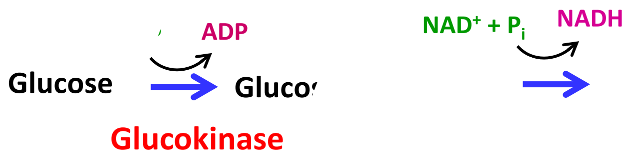

Supplementary Figure 4. Coupled enzyme format used to monitor glucokinase activity. Glucokinase concentration was fixed while glucose-6-phosphate dehydrogenase and the co-substrates were in vast excess and saturating.

Apparent kinetic parameters for glucokinase were determined in a coupled assay format by measuring NADH formation. Stock solutions of 1 µM glucokinase and 1 µM 520 QD capped with CL4 were prepared in 250 mM HEPES buffer pH = 7.44. Briefly, 42 pmol of glucokinase were allowed to assemble to 0 to 210 pmol of QDs at 4 ºC for at least 30 min in 5 mL tubes. The volume was brought up to 2.1 mL with HEPES buffer. Stock substrate solutions consisting of 20 mM $MgCl_2$, 20 mM ATP, 13 U/mL glucose-6-phosphate dehydrogenase, 400 µM NAD^+, and glucose ranging in concentration from 5 to 10,000 µM in water and 1 M HEPES. Briefly, 10 µL of sample and 30 µL of substrate was added to each well of a 384-well plate in a similar manner as described above. The final concentration of the components are as follows: 5 nM glucokinase, 0 to 25 nM 520 QD, 15 mM $MgCl_2$, 15 mM ATP, 10 U/mL glucose-6-phosphate dehydrogenase, 300 µM NAD^+, 3.4 to 7500 µM glucose, and 250 mM HEPES, pH=7.4. Absorbance of NAD at 340 nm was measured utilizing a Tecan Spark plate reader, taking a measurement every 30 s as described previously. Concentration values were determined utilizing Beer–Lambert equation where ε = 6220 M^{-1} cm^{-1} and the path length (b) was 0.413 cm. The apparent kinetic values where determined in the same manner as described above.

Phosphoglucose isomerase (PGI):

PGI catalyzes the conversion of glucose-6-phosphate to fructose-6-phosphate and is also capable of working in the reverse direction. To take advantage of measuring a colorimetric product, we characterized PGI's ability to catalyze fructose-6-phosphate to glucose-6-phosphate conversion utilizing Abcam's Phosphoglucose Isomerase Colorimetric Assay Kit which produces a colorimetric signal *via* an included probe. Briefly, 0.1 pmol of PGI was allowed to assemble 0 to 1 pmol 520 QD capped with CL4 at 4 ºC for at least 30 min in buffer provided by the kit in 1.5 mL Eppendorf tubes. After incubation, the total volume was brought up to 400 μL. 5.5 mg of fructose-6-phosphate was dissolved in 0.6 mL of buffer. The kit instructions were followed except the volumes were scaled down for use in 384 well plate format and the kit substrate was replaced with our stock of fructose-6-phosphate that was diluted from 50 to 0.08 mM to create substrate mixes. To a 384 well plate, 12.5 μL of sample and 12.5 μL of the substrate mix was added to each well in a similar manner as described for amylase. The final concentration of PGI was 105 pM while the QD concentration ranged from 0 to 1.05 pM. The final substrate concentration ranged from 6.55 to 4,000 μM. Absorbance at 450 nm was measured every 30 sec utilizing a plate reader as described for amylase. Absorbance values were converted to concentration values utilizing a standard curve following the kit's instructions and the kinetic parameters were calculated as described for amylase.

Phosphofructokinase (PFK):

Phosphofructokinase catalyzes the conversion of fructose-6-phosphate to fructose-1,6-bisphosphate and its activity on or off QD's was assessed with Sigma-Aldrich's Phosphofructokinase (PFK) Activity Colorimetric Assay Kit. Briefly, enzyme was assembled to 520 QDs capped with CL4 in a similar manner to that described above. The substrate in the kit was replaced with 8 mg of fructose-6-phosphate dissolved in 0.5 mL of assay buffer. The substrate was diluted while keeping the rest of components of the assay kit constant such that the final concentration of fructose-6-phospate ranged from 19.5 to 10,000 μM. To a 384 well plate, 12.5 μL of enzyme-QD bioconjugate and 12.5 μL of substrate mix was added and absorbance was measured at 450 nm over time with a plate reader as described for amylase. Absorbance values were converted to concentration values using a standard curve following the kit's instructions and kinetic parameters determined as described above. The final concentration of enzyme was 3 nM while the QD concentration varied from 0 to 30 nM.

Fructose-bisphosphate Aldolase (FBA):

Fructose-bisphosphate aldolase catalyzes the conversion of fructose-1,6-bisphosphate to D-glyceraldehyde 3-phosphate and dihydroxyacetone phosphate. The activity of FBA was determined utilizing Sigma-Aldrich's Aldolase Activity Colorimetric Assay Kit. Enzyme was assembled to 520 QDs capped with CL4 as described above where the final enzyme concentration was 3 nM while the final QD concentration varied from 0 to 30 nM. The substrate from the kit was replaced with a 16 mg/mL fructose 1,6 bisphosphate solution and the final substrate concentration ranged from 19.5 to 10,000 μM. The kit instructions were followed but scaled down for use in a 384 well plate. The kinetic variables were determined in a similar manner as that described above.

Triose phosphate isomerase (TPI):

Triose phosphate isomerase catalyzes the interconversion of dihydroxyacetone phosphate and D-glyceraldehyde 3-phosphate. Abcam's Triose Phosphate Isomerase Activity Assay Kit was utilized to determine the apparent kinetic characteristics of TPI. The final concentration of TPI was kept constant at 3 nM, while the 520 QDs capped with CL4 concentration ranged from 0 to 30 nM. Bioconjugates were assembled in a similar manner as described above. The substrate from the kit, dihydroxyacetone phosphate, was weighed and found to have an average weight of 2.34 mg which was dissolved in 55 μL of assay kit buffer. The kit instructions were followed with the exception of the substrate, which was diluted to have an estimated final concentration ranging from 9 to 4753 μM dihydroxyacetone phosphate. The absorbance was measured at 450 nm in a similar manner as described above. Analysis to determine apparent kinetic values was also performed in a similar manner as described for amylase.

Glyceraldehyde-3-phosphate dehydrogenase (GPD):

Glyceraldehyde-3-phosphate dehydrogenase (GPD) catalyzes the conversion of glyceraldehyde 3-phosphate to 1,3-bisphosphateglycerate utilizing NAD^+ as a cofactor. The formation of NADH was monitored over time by measuring the absorbance at 340 nm utilizing a plate reader as described for amylase and other enzymes above. Briefly, GPD was allowed to self-assemble to 520 QDs capped with CL4 in a similar manner as described above with a final concentration of GPD at 3 nM and QD final concentration ranging from 0 to 12 nM. The substrate solution consisted of 300 μM NAD^+, 4 mM phosphate solution, and glyceraldhyde-3-phosphate ranging in final concentration from 39 to 10,000 μM. The phosphate solution consisted of equimolar amounts of monobasic phosphate and dibasic phosphate. Measurement and analysis was performed in a similar manner as described for amylase and other enzymes above.

Phosphoglycerate kinase (PGK):

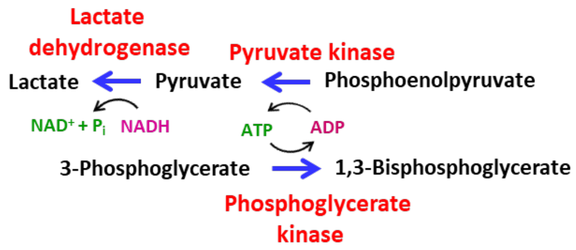

Supplementary Figure 5. Coupled enzyme format used to monitor phosphoglycerate kinase activity. Phosphoglycerate kinase concentration was fixed while the other enzymes and co-substrates were in vast excess and saturating.

Phosphoglycerate kinase catalyzes the conversion of 1,3-bisphosphoglycerate to 3-phosphoglycerate and also functions in the reverse direction. In the reverse reaction, ATP is converted to ADP which when coupled with LDH and PykA activity can be monitored by NADH consumption. This enables us to determine the apparent kinetic variables of PGK when in the presence of saturating amounts of LDH and PykA. PGK was allowed to assemble to 520 QDs capped with CL4 in for at least 2 h at 4 ºC in a similar manner as described above followed by the addition of 150 fold excess peptide (sequence N-C terminus: GSGAAALSHHHHHH-CONH $_2$; where the C-terminus is blocked with an amide) compared to QD. The peptide was synthesized as described in refs.[6-8] and was utilized to assemble to and block any open space left on the QDs to prevent LDH and PykA, which were present in solution, from also attaching to QD.[9-11] Following the addition of peptide, the bionjugates were allowed to assemble for at least 2 h more at 4 ºC. Substrate and the bioconjugates where dispensed to a 384-well plate as described for amylase and other enzymes above. The final PGK concentration was maintained at 3 nM while the QD concentration ranged from 0 to 30 nM and the peptide concentration range from 0 to 4.4 μM. The substrate solution consisted of the following components at their final concentrations: 15 mM $MgCl_2$, 10 mM ATP, 5 mM phosphenolpyruvate, 1.5 mM NADH, 200 nM LDH, 600 nM PykA, 250 mM HEPES, and 78 to 10,000 μM 3-phosphoglycerate. The decrease in absorbance at 340 nm was measured over time with a plate reader in a similar manner describe above. Absorbance values where converted to NADH concentration as described for amylase and the absolute value of initial rates from the linear portions of the curves where utilized to create data points that could be fitted to the Michaelis-Menten equation employing Sigma Plot's enzyme module.

Phosphoglycerate mutase (PGM):

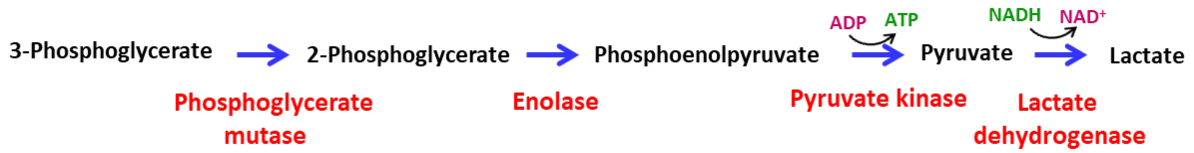

Supplementary Figure 6. Coupled enzyme format used to monitor phosphoglycerate mutase activity. Phosphoglycerate mutase concentration was fixed while the other enzymes and co-substrates were in vast excess and saturating.

Phosphoglycerate mutase catalyzes the conversion of 3-phosphoglycerate to 2-phosphoglycerate by transferring the phosphate group from the C-3 carbon of 3-phosphoglycerate to the C-2 carbon. A coupled assay format was utilized to measure the apparent kinetics parameters of PGM with saturating amounts of Eno, Pyk, and LDH. The consumption of NADH was measured over time in similar manner as described for PGK. PGM was allowed to assemble to 520 QDs capped with CL4 as described for amylase with a final enzyme concentration of 12 nM while the QD concentration varied from 0 to 120 nM. Excess scFv TNT-$(His)_{12}$ protein, as described in refs.,[12-16] was utilized to fill in free space on the QD surface as in the above example after allowing PGM to assemble on the QDs for at least 2 h at 4 ºC. A 30-fold excess of protein to QD was utilized to ensure all free space on the QD surface was occupied by allowing the protein to self-assemble *via* poly-histidine metal affinity coordination chemistry for at least 2 h at 4 ºC. The final concentration of protein varied from 0 to 3.6 µM. Substrate solutions consisted of 3-phosphoglycerate ranging in concentration from 625 to 80,000 µM, 15 mM $MgCl_2$, 10 mM ADP, 1.5 mM NADH, 4 mM phosphate, 3 µM Eno, 3 µM LDH, 6 µM PykA, and 250 mM HEPES. The decrease in absorbance at 340 nm was measured with a plate reader as described previously and the initial rates were calculated determined utilizing the Michaelis-Menten equation as above.

Enolase (Eno):

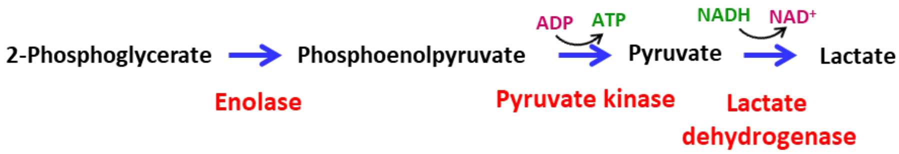

Supplementary Figure 7. Coupled enzyme format used to monitor enolase activity. Enolase concentration was fixed while the other enzymes and co-substrates were in vast excess and saturating.

Enolase catalyzes the conversion of 2-phosphoglycerate to phosphoenolpyruvate. To measure the apparent kinetic parameters, a coupled assay format was utilized with saturating amounts of PykA and LDH where NADH consumption was measured over time similar in manner as described for PGK. Eno was allowed to assemble to QDs as described for amylase with a final enzyme concentration of 3 nM while the QD varied from 0 to 30 nM. Excess blocking peptide was again employed to fill in space on the QD surface, preventing the binding, of LDH and PykA as described above for PGK. The final concertation of peptide to QD varied from 0 to 4.5 μM. The substrate solutions consisted of 15 mM $MgCl_2$, 10 mM ADP, 1.5 mM NADH, 0.6 μM LDH, 1 μM PykA, 250 mM HEPES and ranged in concentration from 78 to 10,000 μM 2-phosphoglycerate. The decrease in absorbance at 340 nm was measured with a plate reader as described previously. The initial rates were calculated and fitted with the Michaelis-Menten equation as described above for PGK.

Pyruvate kinase A (PykA) and lactic acid dehydrogenase (LDH):

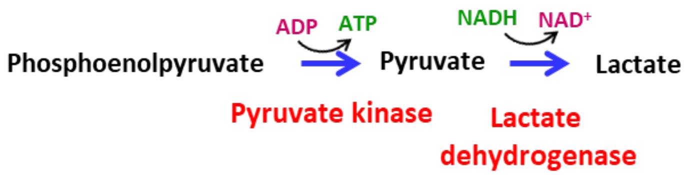

Supplementary Figure 8. Coupled and direct enzyme format used to monitor pyruvate kinase and lactic acid dehydrogenase activity. For the coupled assay, pyruvate kinase concentration was fixed while the other enzyme and co-substrates were in vast excess and saturating.

Pyruvate kinase and lactic acid dehydrogenase activity on and off 520 QDs capped with CL4 were assayed using the methodology originally described in ref.[17] The apparent kinetic parameters of LDH were assayed by measuring the consumption of NADH over time as pyruvate was converted to lactate. LDH was attached to NPs such as QDs and NPLs at ratios ranging from 0 to 12. Stock solutions of LDH at 0.35 µM, 520 CL4 QD at 0.35 µM or 0.035 µM, and NPL at 0.35 µM or 0.035 µM were made in 250 mM HEPES buffer, pH = 8.5. To 1.5 mL Eppendorf tubes, 3.5 pmol of LDH was added followed by 0 to 14 pmol of QD or NPL. The solution was allowed to assemble for at least 30 min at 4 ºC followed by the addition of 250 mM HEPES buffer until reaching the final volume of 700 µL. Stock substrate solutions were made by serially diluting a 112 mM pyruvate solution in 250 mM HEPES buffer pH = 8.5. Pyruvate stock solutions ranged in concentration from 0.9 to 112 mM. To a 384 well plate, 25 µL of LDH free in solution or attached to a NP was added followed by 25 µL of the substrate solution containing pyruvate, $MgCl_2$, and NADH in a similar manner as described previously. The final concentrations of each component in the well is as follows: 2.5 nM LDH, 0 to 10 nM NP, 400 µM NADH, 15 mM $MgCl_2$, and 0.4 to 54 mM pyruvate. The plate was immediately put into a Tecan Spark plate reader and the absorbance was read at 340 nm over time in a similar manner as described for amylase. The apparent kinetics parameters were determined in a similar manner as described above.

7 enzyme assays with 5 nm diameter AuNP scaffolds. 5 nm diameter AuNPs were prepared with surfaces containing 50% TA / 50% NTA ligands. The night before their intended use, an aliquot of AuNPs were incubated in a 200 fold excess amount of $NiCl_2 \cdot 6H_2O$. The solution was brought up to a final volume to achieve a stock solution of 425 nM AuNPs and 85 μM Ni^{+2}. Samples were incubated with the 7E cascade as described previously but allowed to assemble overnight. Aliquots of each sample were added to 384-well plate and the NADH formation followed over time as described in previous sections.

7 enzyme assays with commercial 525 ITK carboxyl QD scaffolds. The 7E system was assembled to 525 ITK carboxyl QD scaffolds in the same manner as described previously with only one modification. An aliquot of 8 μM ITK carboxyl QDs was incubated in the presence of 200-fold excess $NiCl_2 \cdot 6H_2O$ overnight. The divalent nickel coordinates with the terminating carboxyl functional groups of the stabilizing ligands and coordinates with the polyhistidine tail of the enzymes. The following day, the stock solution was brought to a final volume using 250 mM HEPES to achieve a 425 nM ITX carboxyl QD and 85 μM Ni^{2+} solution, then subsequently aliquoted as described above under the same reaction conditions.

7 enzyme assays with dendrimer scaffold. Assays contained 13.8 nM Glk, 2.5 nM PGI, 22.5 nM PFK, 30 nM Ald, 2.5 nM TPI, 67.5 nM GAPDH, 18.8 nM PGK, 15 mM $MgCl_2 \cdot 6H_2O$, 7.5 mM ATP, 7.5 mM ADP, 10 mM glucose monohydrate, 4 mM dibasic/monobasic phosphate, and 2.25 mM NAD^+ in 250 mM HEPES at 30 C. Dendrimer stock was incubated with 10× $NiCl_2 \cdot$6H2O overnight prior to enzyme immobilization. Enzyme samples were allowed to assemble at 4°C for 5 hours prior to running the assay.

Enzyme ratios per NP utilized.

Several different sets of enzyme ratios per NP were utilized throughout this study and these are listed in **Supplementary Table 2**. As indicated, the empirical set of ratios for the 7E system was chosen based on considering relative activity of each enzyme and then trying to intuit a working set for initial proof-of-concept experiments. Later studies utilized 'optimized' ratios that were based on numerical modeling of the glycolytic cascades. A detailed description of the modeling is provided below. From the numerical simulations, an initial 'optimized' set of ratios were obtained, which was then slightly improved upon by serial testing in side-by-side assays against other sets of ratios where minor changes were made in the stoichiometry of a particular enzyme relative to the rest present. The results of these assays were compared to each other and iterative changes made until the ratios in **Supplementary Table 2** were reached. Further attempts to optimize the ratios did not yield significantly better results. An example of how enzyme ratios evolved in this process for the 7E system is provided below in **Supplementary Table 1**. From this Table, one can see that after numerical optimization, the values of each number did not vary typically more than 2-fold and their magnitude relative to each other was remarkably consistent; the importance of the latter point, which concerns relative ratios being maintained, is made clearer in the subsequent description and discussion of the kinetic modeling.

Supplementary Table 1. Example of an evolution of enzyme ratios utilized

**Enzyme**	**Empirical**	**Opt 1 (k_{cat}/K_M Supp. Table 4)**	**Opt 2 initial (Optimal Supp. Table 5)**	**Opt 2 final used in experiments**
**Glk**	**1**	**1.5**	**8.7**	**5.5**
**PGI**	**1**	**1**	**2**	**1**
**FPK**	**10**	**9**	**12.6**	**9**
**FBA**	**10**	**12**	**13**	**12**
**TPI**	**1**	**5**	**1.2**	**1**
**GPD**	**10**	**27**	**13.9**	**27**
**PGK**	**0.5**	**7.5**	**11.6**	**7.5**

Notes: Values shown are the ratio of a given enzyme added per unit QD/NPL (1) present in the assembly solution. The actual amount of enzyme added or present in an assay will vary as a function of the final QD/NPL concentration. For example, using the 7E system at Opt 2 final ratios, the listed values are the number of each enzyme added per QD/NPL present in that reaction. Supp. – Supplementary.

Supplementary Table 2. Enzyme ratios utilized in different experimental configurations

	7 Enzymes (7E)						8-10 Enzymes			4 Enzymes
**Enzyme**	**Empirical**	**Opt 1**	**Opt 2**	**High ptn**	**Med ptn**	**Low ptn**	**7E + Amy/Malt**	**7E + Inv**	**7E + Amy/Malt/Inv**	**4E**
**Amy**	--	--	--	--	--	--	6.4	--	6.4	--
**Mal**	--	--	--	--	--	--	8.5	--	8.5	--
**Inv**	--	--	--	--	--	--	--	11.9	11.9	--
**Glk**	1	1.5	5.5	15	1.5	0.2	7.5	7.5	7.5	--
**PGI**	1	1	1	2.7	0.3	0.03	2	2	2	--
**FPK**	10	9	9	24.5	2.5	0.31	10	10	10	--
**FBA**	10	12	12	32.7	3.3	0.41	12	12	12	--
**TPI**	1	5	1	2.7	0.3	0.03	1	1	1	--
**GPD**	10	27	27	73.6	7.4	0.93	27	27	27	--
**PGK**	0.5	7.5	7.5	20.5	2	0.26	9.5	9.5	9.5	--
**PGM**	--	--	--	--	--	--	--	--	--	18
**Eno**	--	--	--	--	--	--	--	--	--	8
**PykA**	--	--	--	--	--	--	--	--	--	19
**LDH**	--	--	--	--	--	--	--	--	--	19
**Used in Figs:**	3a	3a	3a-f, 4a-c 5a,b,d-f, 6f,7b,c,e,f,h,i, SI 33,SI 41, SI 42, SI 44-49	SI 28 SI 29	SI 28 SI 29	Fig S28 Fig S29	5c 6b,c	6a	6f	6e,f SI 4

Notes: Values shown are the ratio of a given enzyme added per unit QD/NPL (1) present in the assembly solution. Where applicable, figures indicate the concentrations of QD/NPL utilized. The actual amount of enzyme added or present in an assay will vary as a function of the final QD/NPL concentration. For example, using the 7E system at Opt 2 ratios, the listed values are the number of each enzyme added per QD/NPL. SI – Supplementary Information.

Kinetic simulations

Modeling of glycolytic cascades assembled on nanoparticles. The modeling approach used in this paper is the same as that employed in [17, 18] and those references should be consulted for a more in-depth discussion of the underlying assumptions and methods. Here, we give an abbreviated summary with focus on certain critical aspects. The approach is macroscopic, and we assume the diffusion times of all solutes to be short compared to the reaction times so that a chemical kinetic description is appropriate. All of the enzyme reactions both in solution and when conjugated to NPs are taken to obey Michaelis-Menten (MM) kinetics in order to utilize the corresponding kinetic descriptors derived using that formalism. For simplicity and because of our interest in channeling, we generally ignore back-reactions (*i.e.*, those of gluconeogenesis) and product inhibition, though as reactants build up these effects are known to occur[19, 20] and indeed are seen in some of our experiments at long times. To avoid the latter issue, all of our kinetic values are determined from either the initial log phase growth or the linearly increasing portions of the progress curves.

For clarity in illustrating methods, we here write out the reactions for a four-enzyme system consisting of phosphoglycerate mutase (denoted PGM or E_1 below), enolase (Eno or E_2), pyruvate kinase (PykA or E_3), and lactate dehydrogenase (LDH or E_4). The initial substrate (S) is 3-phosphoglycerate (or 3-PG), the final product (P) is lactate, and the turnover is monitored by using UV-Vis to follow the conversion of NADH (N) to NAD$^+$ (N_+). The reactions of this system are assumed to be of MM form:

$$E_1 + S \underset{k_{-1}}{\overset{k_1}{\rightleftarrows}} C_1 \xrightarrow{k_{cat1}} E_1 + I_1 \qquad E_2 + I_1 \underset{k_{-2}}{\overset{k_2}{\rightleftarrows}} C_2 \xrightarrow{k_{cat2}} E_2 + I_2$$

$$E_3 + I_2 + A_2 \underset{k_{-3}}{\overset{k_3}{\rightleftarrows}} C_3 \xrightarrow{k_{cat3}} E_3 + I_3 + A_3$$

$$E_4 + I_3 + N \underset{k_{-4}}{\overset{k_4}{\rightleftarrows}} C_4 \xrightarrow{k_{cat4}} E_4 + P + N_+ + P_I$$

(Supplementary Eq. 1)

In these reactions, the enzyme-substrate complexes and the reaction intermediates are symbolized by C_i and I_i, respectively, while P_I denotes a phosphate ion (*e.g.*, $H_2PO_4^-$). Assuming well-stirred conditions and lowest order kinetics (and writing the concentrations of each reactant/product by

the corresponding small letters), the reactions in (Supplementary Eq. 1) will be governed by the following ordinary differential equations (ODEs):

$$\frac{ds}{dt} = -k_1 e_1 s + k_{-1} c_1 \qquad \frac{dc_1}{dt} = k_1 e_1 s - (k_{-1} + k_{cat1}) c_1$$

$$\frac{di_1}{dt} = k_{cat1} c_1 - k_2 e_2 i_1 + k_{-2} c_2 \qquad \frac{dc_2}{dt} = k_2 e_2 i_1 - (k_{-2} + k_{cat2}) c_2$$

$$\frac{di_2}{dt} = k_{cat2} c_2 - k_3 e_3 i_2 a_2 + k_{-3} c_3 \qquad \frac{dc_3}{dt} = k_3 e_3 i_2 a_2 - (k_{-3} + k_{cat3}) c_3$$

$$\frac{di_3}{dt} = k_{cat3} c_3 - k_4 e_4 i_3 n_p + k_{-4} c_4 \qquad \frac{dc_4}{dt} = k_4 e_4 i_3 n_p - (k_{-4} + k_{cat4}) c_4$$

$$\frac{di_4}{dt} = k_{cat4} c_4 \qquad \frac{da_2}{dt} = -k_3 e_3 i_2 a_2 - k_{-3} c_3 \qquad \frac{dn_p}{dt} = -k_4 e_4 i_3 n_p - k_{-4} c_4$$

(Supplementary Eq. 2)

with $e_{0i} = e_i + c_i$ where e_{0i} is the known initial concentration of each enzyme. This coupled set of 11 differential equations is readily integrated numerically; for this purpose we use MATLAB and specifically their stiff solver ode15s.

The kinetic coefficients in (Supplementary Eq. 2), or in analogous equations for other cascades, are estimated using individual control experiments in which all but one enzyme are included in excess so that each such experiment is rate-limited by the single enzyme of interest. Coefficient values determined in this way are listed in the main manuscript **Table 2** for the various enzymes either free in solution or attached to NPs. Given that these values are measured in independent experiments (and assuming the NP values remain the same whether the NP is by itself or part of a cluster), we are then in position to make predictions of cascade behavior and to compare with experiment as a test of our understanding. One use of such simulations (see the next section) is to predict the relative enzyme concentrations in the cascade that most efficiently generate final product. The optima obtained from these studies are then used as an initial basis for the final experimental ratios as discussed in the section above. A second application of simulation, pursued previously in ref.[17] and ref.,[21] is as a tool for investigating whether the experimentally observed turnover is being sped up by channeling. In this regard, we can test a channeling hypothesis at any step of the cascade by examining what we call "maximum channeling". In this limit, the intermediate for any particular step is assumed to be delivered from the previous step directly to the next enzyme without being slowed by diffusion/dilution. In other words, that intermediate is effectively present at much higher concentration and so the corresponding rate is elevated.

“Maximum channeling” means this rate is so high that it is no longer a rate-limiter in the overall process and can be ignored. From this description it is clear that this type of approximation can be investigated at each step individually and/or at all steps simultaneously.   Comparisons with experiment are then used to draw conclusions as discussed below in the section on channeling.

Optimization of turnover. As a crude basis for designing an enzyme cascade, one can observe that for all but the first enzyme the corresponding substrates (*i.e.*, the intermediates of the cascade such as the I_i in Supplementary Eq. 1) will initially be at very low concentration and so one might expect the turnover from these component reactions to be dominated by their respective catalytic efficiencies k_{cat}/K_M. With this as a guide, a simplest choice for the optimal enzyme concentrations would be to take them in inverse proportion to k_{cat}/K_M, and values chosen according to this rule for 4, 7, and 9 enzyme cascades are given in **Supplementary Tables 3** and **4** under the heading of k_{cat}/K_M. The only enzyme not subject to this argument is the first since the starting substrate is present at high concentration, and so its turnover would instead seem set by k_{cat}. One can thus expect that the optimum concentrations of this first enzyme will likely be different from the values predicted by k_{cat}/K_M.

Supplementary Table 3. Optimized ratios for 4 enzyme cascade*

Enzyme	Free		On QD	
	k_{cat}/K_M	Optimal	k_{cat}/K_M	Optimal
PGM	55.4	17.9	51.4	16.7
Eno	1.4	7.7	6.3	8.1
PykA	1.0	18.7	1.0	19.0
LDH	5.2	18.8	4.3	19.2

*The values in each column of this table, and **Supplementary Table 4** below, add to 63 and would give the enzyme/QD ratio if each QD carried 63 enzymes. Since only the ratios among these numbers are meaningful, any other normalization may be used.

Supplementary Table 4. Optimized ratios for 7 and 9 enzyme cascades

Enzyme	7 Enzyme		9 Enzyme	
	k_{cat}/K_M	Optimal	k_{cat}/K_M	Optimal
Amy			3	5.4
Mal			8	6.8
Glk	1.5	8.7	1.3	7.4
PGI	1	2	1	2
FPK	9	12.6	7.4	10
FBA	12	13	10	10.6
TPI	5	1.2	4.1	0.5
GPD	27	13.9	22	10.8
PGK	7.5	11.6	6.2	9.5

To do a better job of predicting the "best" enzyme concentrations for a given cascade, we can formulate an optimization problem where we look to maximize the amount of final product generated at a specific end time. Since the output will rise with higher enzyme concentrations (assuming all else unchanged), the optimization should be carried out under a constraint such as

fixing the total number of enzymes, in which case we are just optimizing the relative proportions of the different enzymes. A second consideration is the choice of the end time, which we want to be relatively large so that a significant amount of final product forms. However, if our simulation is run with a finite supply of reactants, then the end time should also not be so large as to run into resource limitations. A final point is that in some cases one enzyme is much faster than the others and thus is needed only at very low concentrations so long as it is not entirely absent/inactive. To avoid the latter issue experimentally, we add a constraint that each enzyme must be present at least at some minimum concentration or enzyme/QD ratio.

For a given set of enzyme concentrations, the calculation needed to find the concentration of final product at the specified end time is simply a numerical integration of the governing ODEs, *e.g.*, (Supplementary Eq. 2) or analogous equations for other enzyme cascades. Although this calculation is not especially intensive, an optimization algorithm requires that it be done a great many times and so minimizing the number of these integrations is desirable. A second issue is that derivative information is not available, except by numerical approximation, and so well-established methods of derivative-free optimization would seem to be recommended.[22] In order to get a better view of the issues, we began by using a brute-force search approach. Not surprisingly, this was found to be effective only for systems involving a small number of enzymes, and it became increasingly time consuming (on a desktop computer) when the cascade grew to more than about 7 enzymes. To improve on this we considered several alternative methods and settled on Bayesian optimization because of its reputation for performance with expensive, non-convex cost functions, and without derivative information.[23] In particular, we employed a hybrid method known as Bayesian adaptive direct search (or BADS) that combines a search algorithm with Bayesian optimization in order to achieve a good balance between exploration and exploitation.[24]

The kinetic coefficient values obtained for the 4, 7, and 9 enzyme cascades by numerical optimization are given in **Supplementary Tables 3** and **4** under the heading of Optimal. The values obtained can be seen to be rather different from those based on k_{cat}/K_M, and this is especially so for the case with fewest enzymes where the poor assumption mentioned earlier about the first enzyme is most impactful. As an illustration of the improvements possible by using the numerical optimization, in **Supplementary Figure 9** we plot the generation of final product *versus* time for the 7 and 9 enzyme cascades (where the saturation at long times is due to the consumption of all of the starting substrate). The differences in the predictions of the two approaches to optimization

exceed a factor of 2 at long times. The fact that for a 9 enzyme system these time savings are on the order of a day or more makes them seem especially significant. It should also be noted that these optima assume no channeling is occurring, and as we see in the next section such channeling can greatly accelerate the turnover. Examples of how these optimized enzyme ratios led to the actual ratios used in experiments and the improvements seen in turnover are given in the preceding section and in the main manuscript.

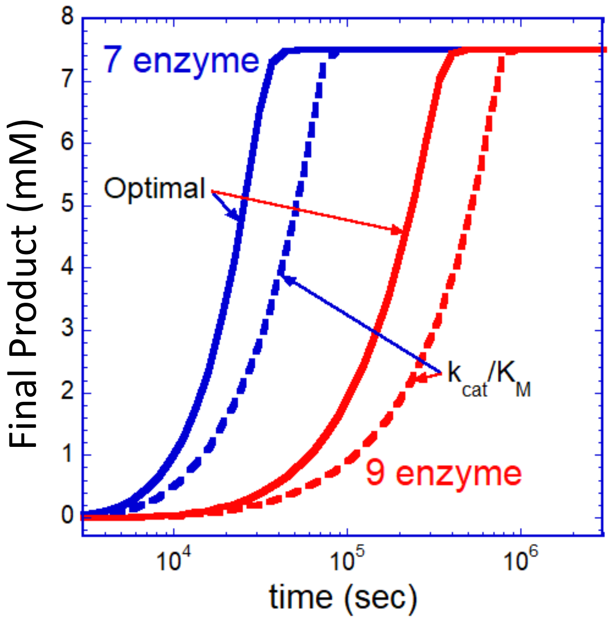

Supplementary Figure 9. Predictions of the optimal generation of product for the 7 and 9 enzyme cascades with the optima found either analytically based on the values of k_{cat}/K_M (dashed) or numerically (solid).

Possibilities of channeling. When combined with the coefficient values of manuscript **Table 2** measured in independent experiments, kinetic modeling of the type described above can open up a line of evidence regarding channeling as was previously pursued in refs.[17, 21] Here, we discuss this in the context of the 4 enzyme system for which some experimental data is shown in **Supplementary Figure 10**. The corresponding simulations in **Supplementary Figure 11** have the same enzyme ratios as were used experimentally (which were the optimal values given in **Supplementary Table 3** for the free system). Seen on the left in **Supplementary Figure 11A** are simulations assuming the QD concentration is 50 nM. If no channeling is present (brown curve), then the prediction (again based on coefficient values measured in independent experiments) is far slower than what is seen experimentally (green curve in **Supplementary Figure 10**). This is evidence that a speed-up due to channeling is occurring experimentally in the 4-enzyme cascade. To learn more about this channeling, in **Supplementary Figure 11A** we also show the results of various versions of maximum channeling, *viz*., operating only on the 1st intermediate (2-phosphoglycerate, red curve), only on the 2nd (phosphoenolpyruvate, blue curve), only the 3rd (pyruvate, green curve), and on all three intermediates (light blue curve). Of these, we can see that only the case with maximum channeling of the pyruvate corresponds well with experiment. The plot on the right in **Supplementary Figure 11B** examines this case further for other QD concentrations, and the agreement with experiment (**Supplementary Figure 10**) is quite good. The main disagreement is an initial delay that is seen experimentally but not in the simulation. The delay or lag time to linear increase in experiments is due to the slow buildup of the reaction flux to get to the steady state (due to the high K_M and ΔG values) as discussed below.

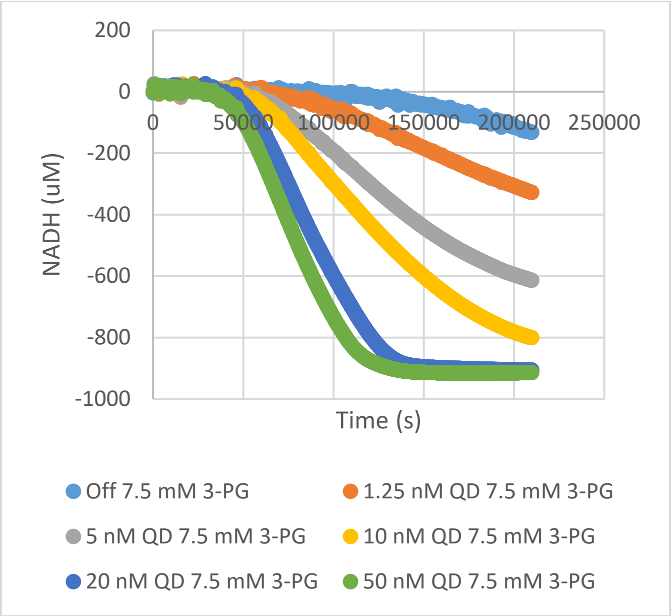

Supplementary Figure 10. Experimental data for the disappearance of the initial NADH with time as the 4-enzyme reaction proceeds. The amount of starting substrate (3-PG) is fixed and each trace has a different concentration of QDs (and hence more enzymes present per cluster at the same fixed ratios).

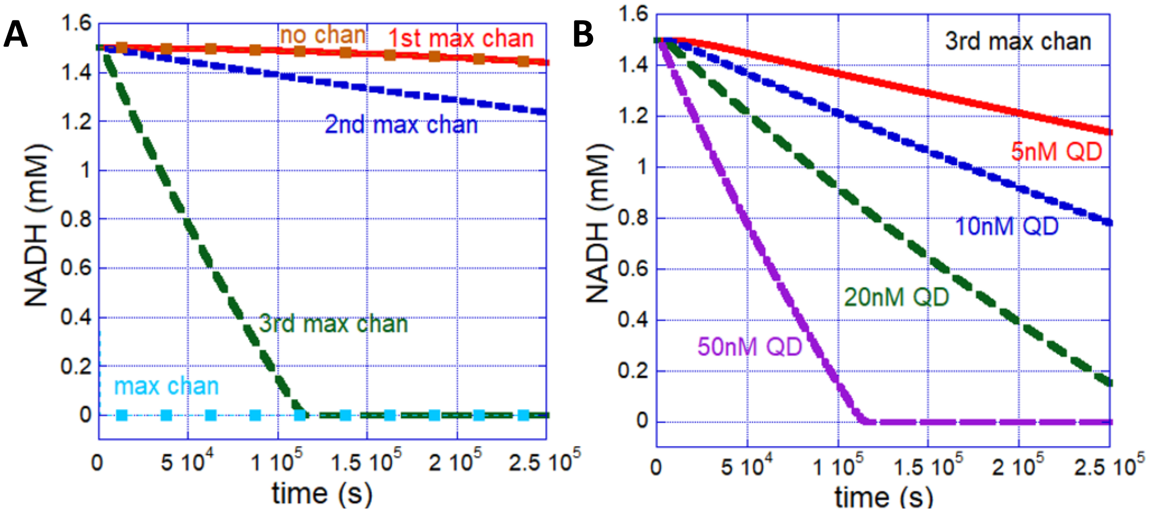

Supplementary Figure 11. Simulation plots that are analogous to the experimental plot of **Supplementary Figure 10**. **(A)** Predictions with no channeling and with various versions of maximum channeling - 1st, 2nd, or 3rd intermediate only, and all intermediates for a 50 nM QD concentration. The no channeling case is far slower and the full channeling case is far faster than experiment, whereas assuming only the 3rd intermediate is channeled gives reasonable agreement. **(B)** Plots with maximum channeling of only the 3rd intermediate for various QD concentrations as in **Supplementary Figure 10**.

Thermodynamic analysis and ΔG. The basic equation for determining the Gibbs free energy is given by:[25]

$$\Delta G = \Delta G^0 + RT\ln K, \qquad \text{(Supplementary Eq. 3)}$$

where R is the universal gas constant (8.314 J mol^{-1} K^{-1}), T is temperature (298.15 Kelvin = 25 °C), K = equilibrium constant (or, K can be replaced with J^+/J^-). At equilibrium, ΔG = 0 and RTlnK = ΔG^0. There are 4 main values of ΔG relevant to glycolysis:

- $\Delta_r G^0$ = the ΔG at "standard conditions", usually 25 °C, 1 bar pressure, 1M of substrate and 1M of product, ***without*** taking into account pH or ionic strength
- $\Delta_r G'^0$ = the ΔG at "standard conditions", usually 25 °C, 1 bar pressure, 1M of substrate and 1M of product, ***with*** taking into account pH or ionic strength
- $\Delta_r G'$ = the ΔG at ***actual reaction concentrations***, usually 25 °C, 1 bar pressure, ***with*** taking into account pH or ionic strength
- $\Delta_r G'^m$ = the ΔG at "***biochemical*** standard conditions", usually 25 °C, 1 bar pressure, 1 ***mM*** of substrate and 1 ***mM*** of product, ***with*** taking into account pH or ionic strength

To help understand the 7 enzyme (7E) and 4 enzyme (4E) pathways, we sought to estimate their thermodynamic parameters using the program eQuilibrator 3.0 (https://equilibrator.weizmann.ac.il/static/classic_rxns/about.html#about-equilibrator).[25] This program gives estimates of $\Delta_r G'^m$ values (rather than the more common $\Delta_r G'^0$), as seems preferable given that it more closely approximates the metabolite concentrations in cells and in the pathways studied herein. Even better would be $\Delta_r G'$ but determining the needed concentrations throughout the entire course of the reactions was beyond the scope of this work. For the calculations, the pH was assumed to be the pH of the buffer (pH 8) since the buffer was at a high relative concentration (250 mM). The ionic strength was determined using the equation $½\Sigma(c*z^2)$ where c = concentration and z = charge. Although the HEPES buffer is zwitterionic and therefore does not contribute *per se* to ionic strength,[26] its pH was adjusted to 8 with NaOH which then contributes. To this end, the web-based calculator (https://www.liverpool.ac.uk/pfg/Tools/BuffferCalc/Buffer.htm) was used and the ionic strength of the buffer at 25 °C was estimated to be 187 mM. The ionic strengths for the 7E system was calculated as per **Supplementary Table 5** and for the 4E system in **Supplementary Table 6**. For

the combined 11 enzyme pathway the ionic strength was assumed to be the average. Any changes in pH or ionic strength resulting from the addition of the NPs or enzymes ignored because of their low concentrations.

Supplementary Table 5. Values used for the upstream 7 enzyme cascade (Glk → PGK).

Compound	mM	Ion_1 charge	Ion_2 charge	Calculation	IS (mM)
$MgCl_2$	15	+2	-1	$½[(15*2^2)+(30*1^2)]$	45
ATP-Na_2	7.5	-2	+1	$½[(7.5*2^2)+(15*1^2)]$	22.5
ADP-Na	7.5	-1	+1	$½[(7.5*1^2)+(7.5*1^2)]$	7.5
Glucose	20	0	0	N/A	0
Monobasic phosphate[a]	4	+1	-1	$½[(4*1^2)+(4*1^2)]$	4
Dibasic phosphate[a]	4	+1	-2	$½[(8*1^2)+(4*2^2)]$	12
NAD^+	1.13	+1	N/A	$½[(1.13*1^2)]$	0.6
HEPES, pH 8[b]	250				187
**TOTAL**					**278.6**

[a]Phosphate was made from monobasic (NaH_2PO_4) and dibasic phosphate (Na_2HPO_4), 4 mM each.
[b]HEPES ionic strength due to changing pH of buffer.

Supplementary Table 6. Values used for the downstream 4 enzyme cascade (PGM → LDH).

Compound	mM	Ion_1 charge	Ion_2 charge	Calculation	IS (mM)
$MgCl_2$	15	+2	-1	$½[(15*2^2)+(30*1^2)]$	45
ADP-Na	10	-1	+1	$½[(10*1^2)+(10*1^2)]$	10
3-phosphoglycerate-Na_2	7.5	-2	+1	$½[(7.5*2^2)+(15*1^2)]$	22.5
Monobasic phosphate[1]	4	+1	-1	$½[(4*1^2)+(4*1^2)]$	4
Dibasic phosphate[1]	4	+1	-2	$½[(8*1^2)+(4*2^2)]$	12
NADH-Na_2	1.5	-2	+1	$½[(1.5*2^2)+(3*1^2)]$	4.5
HEPES, pH 8[2]	250				187
**TOTAL**					**285**

[1]Phosphate was made from monobasic (NaH_2PO_4) and dibasic phosphate (Na_2HPO_4), 4 mM each.
[2]HEPES ionic strength due to changing pH of buffer.

The eQuilibrator program was used under the Pathway Analysis tab. The example Pathway definition file (example_fermentation.tsv) was modified for each pathway. Specifically, for the 7 upstream enzymes, the last five reactions going from D-glycerate-3-phosphate to ethanol were removed. For the 4 downstream enzymes, the first seven reactions going from glucose to D-glycerate-3-phosphate and the acetaldehyde to ethanol reaction were removed, and the reaction of pyruvate to acetaldehyde was changed to pyruvate + NADH ⇔ Lactate + NAD^+. Note that the flux for reactions D-glyceraldehyde 3-phosphate ⇔ 1,3-bisphosphoglycerate and below was set at 2, whereas for reactions above this the flux was set at 1 to account for the splitting of the glucose molecule. The min and max concentrations were set at the min/max concentration of reactants for each pathway but are immaterial for $\Delta_r G'^m$ values. The pH was set at 8.0, the pMg was set at 1.8 (= rounded -log(0.015)), the ionic strength set (rounded to 0.28 M, 0.29 M, and 0.28 M, respectively) as delineated above, and the Analysis method as MDF. Note that ratios were adjusted for the 7–10 enzyme system with "1" for all enzymes except glyceraldehyde 3-phosphate dehydrogenase and phosphoglycerate kinase, to account for two glyceraldehyde 3-phosphates and two 1,3-bisphosphoglycerates per glucose, respectively. The pathway analysis was then performed, and the $\Delta_r G'^m$ values recorded (**Supplementary Table 7**). The cumulative $\Delta_r G'^m$ values (starting at 0 kJ/mol) and the K_M for the reactions on QDs were then plotted.

Supplementary Table 7. Estimated ΔG values for the 4E and 7E cascades. Values remain unchanged for the entire 11E cascade.

	$\Delta_r G'^m$, all values in kJ/mol		
**Reaction**	**Enzyme**	**7E, $\Delta_r G'^m$**	**4E, $\Delta_r G'^m$**
D-Glucose + ATP <=> D-Glucose 6-phosphate + ADP	Glk	-21.6	
D-Glucose 6-phosphate <=> D-Fructose 6-phosphate	PGI	+7.6	
ATP + D-Fructose 6-phosphate <=> ADP + Fructose-1,6-bisphosphate	FPK	-22.1	
Fructose-1,6-bisphosphate <=> Glycerone phosphate + D-Glyceraldehyde 3-phosphate	FBA	+4.8	
Glycerone phosphate <=> D-Glyceraldehyde 3-phosphate	TBI	+6.1	
Pi + NAD^+ + D-Glyceraldehyde 3-phosphate <=> NADH + 1,3-Bisphosphoglycerate	GPD	+15.9	
ADP + 1,3-Bisphosphoglycerate <=> ATP + D-Glycerate-3-phosphate	PGK	-20.2	
D-Glycerate-3-phosphate <=> D-Glycerate-2-phosphate	PGM		+3.4
D-Glycerate-2-phosphate <=> Phosphoenolpyruvate + H_2O	Eno		-4.2
ADP + Phosphoenolpyruvate <=> Pyruvate + ATP	PykA		-23.1
Pyruvate + NADH <=> Lactate + NAD^+	LDH		-17.4

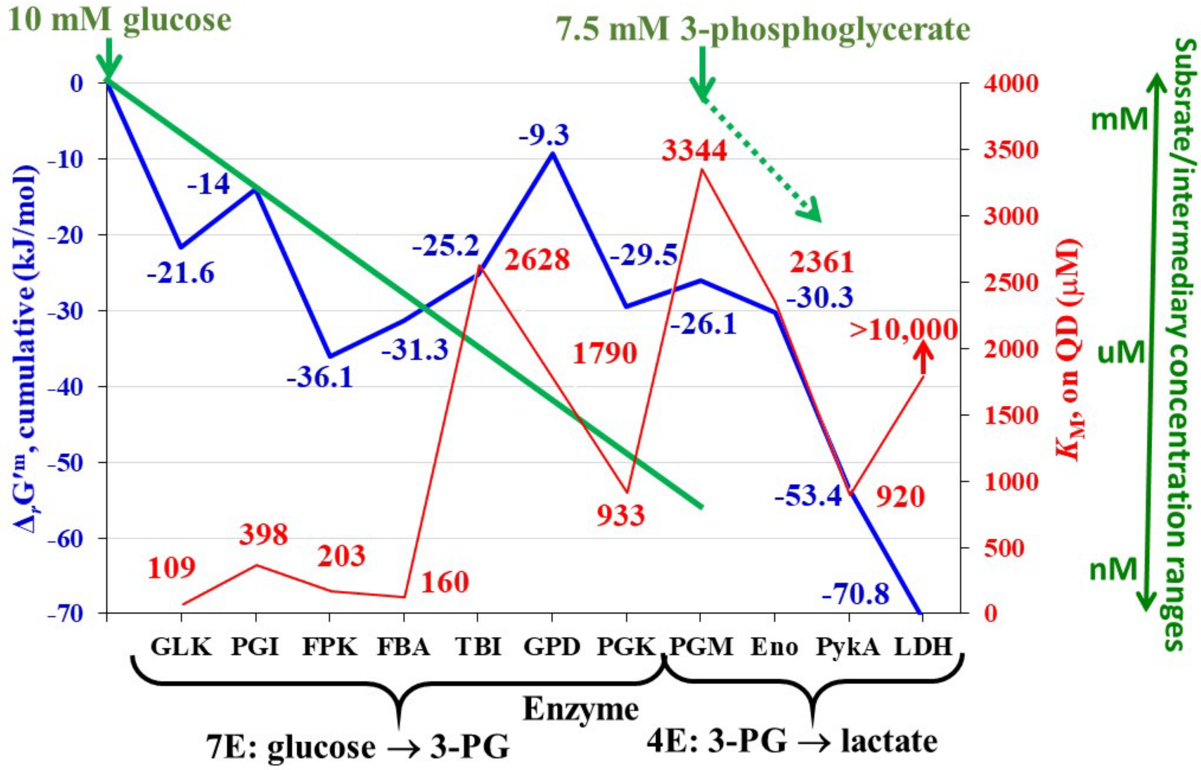

Supplementary Figure 12. Plot of $\Delta_r G'^m$ (kJ/mol), K_M (µM), and approximate substrate/intermediary concentration ranges (log scale) determined from a combined 11 enzyme cascaded assay using LCMS analysis. For this determination, the assays were sampled at t = 40,000 seconds which corresponds to a plateau for the 7E reaction. Sampling of the reactions at 80,000 seconds of later did not increase the apparent concentration of any of the last 3 intermediates in the 4E assay significantly.

In looking at **Supplementary Figure 12**, it is clear that sufficient initial substrate is available going into the 7E cascade (Glk→PGI→PFK→FBA→TBI→GPD→PGK) to drive the first enzyme of the cascaded reaction at near steady state and the cumulative $\Delta_r G'^m$ is favorable as are the K_M's of the first four enzymes to push the reaction forward quite rapidly. This is verified by the approximate concentrations of intermediates measured as well. We believe that this initially high rate of channeled flux in conjunction with the favorable $\Delta_r G'^m$ of PGK is cumulatively sufficient to move the reaction forward and to overcome the unfavorable $\Delta_r G'^m$ and K_M of TBI and GPD while still benefiting from the channeling present. However, in the 11E cascade (Glk→PGI→PFK→FBA→TBI→GPD→PGK→PGM→Eno→PykA→LDH) the amount of 3-PG being encountered by the last 4 enzymes, in the high nM to low uM range, is so low compared to the K_M of each of these enzymes, and especially that of PGM (≥ 100-fold below PGM's K_M),

that most of the reactions do not occur until a much higher amount of 3-PG very slowly builds up. For the latter, so much time is needed that channeling is no longer a factor at all. The latter 4E cascade (PGM→Eno→PykA→LDH) does utilize channeling when initiated with high concentrations of 3-PG as shown in manuscript **Figure 5E** but still requires 10-12 hours to move into concerted log-phase catalytic flux. This complex mismatch of unfavorable $\Delta_r G'^m$, K_M, and low initial substrate concentration do not allow for the subsequent 4E system to be appended to the end of the 7E cascade and still benefit from the presence of the channeling phenomena. In this scenario, intermediary diffusion away to the bulk probably becomes dominant again. Presumably, if the K_M and $\Delta_r G'^m$ values of the 4E system were quite close to that of the 7E's first four (Glk→PGI→PFK→FBA), then channeling would have been seen in the conjoined 11E system.

Mass spectral analysis of substrates, intermediaries, and products. LC-MS analyses were carried out on a Waters Acquity UPLC H-Class (Waters Corp, Milford, MA) equipped with a SQ2 MS detector based on a single quadrupole analyzer and an Electrospray Ionization source as previously described. [17, 27-29] System control and data acquisition was achieved with MassLynx V4.1 software. Chromatographic separations for maltose, glucose, glucose-6-phosphate, fructose-1,6-biphosphate, glyceraldehyde-3-phoshpate and phosphoenolpyruvate were performed using H_2O and methanol both with 0.1% formic acid as mobile phase. Gradient conditions were focused to separate first target analytes to avoid ion suppression from sample matrix components as follows; initial conditions (0% methanol) for 1 min., increased to 80% in 3 min, 100% in 10 min and return to initial conditions in 5 min up to 15 min total run time, with a flow rate of 0.35 mL min^{-1} and a constant column temperature of 50˚C on a C18 BEH (1.7 µm, 2.1 mm x 100 mm). Lactate and pyruvate were separated using on a Glycan BEH Amide (130Å pore size, 1.7 µm 2.1 x 100 mm) using Mobile Phase's A (5:95 acetonitrile: 30mM NH_4COOH) and B (85:15 acetonitrile:30 mM NH_4COOH) in gradient mode. Elution profile was as follows: 100% B for 2 min; 70% B in 8 min and back to initial conditions in 14 min (100% B), with a constant flow rate of 0.4 mL min^{-1} and a constant column temperature of 50 ˚C The quadrupole mass spectrometer was operated in SIR (Single Ion Recording) mode optimized for each individual analyte. Optimized cone voltages for each analyte were the following: maltose (26V); glucose and glyceraldehyde-3-phoshpate (22V); glucose-6-phosphate (30V); fructose-1,6-biphosphate (32V); phosphoenolpyruvate (20V) and pyruvate and lactate (10V). The MS capillary voltage was kept at 3.8 kV. Total injection volume was 2 µL for every sample, except invertase samples, which were injected with 10 µL.

HPLC purification of 3-PG from the 7 enzyme cascade. To purify 3-phosphoglyceric acid (3PG), the 7 enzyme reaction was run as described in a microtiter plate and stored at -80 °C. Plates were thawed, wells combined, methanol and acetonitrile were added, the sample was placed at -20 °C for at least 20 minutes, and then it was centrifuged at 16,000×g for 10 minutes. Supernatant was removed and dried under vacuum. Samples were then purified on a Waters Acquity H-class UPLC using a 4.6 x 150 mm Xbridge BEH Glycan (Amide) XP 2.5 μm column (Waters Corporation, Milford, MA, USA). Buffer A was 95:5 H_2O:acetonitrile with 30 mM ammonium formate pH 3; Buffer B was 15:85 H_2O:acetonitrile with 30 mM ammonium formate pH 3. The following method was used with 50 μL sample injections at 45 °C with a flow rate of 0.8 mL/min: 0 min, 100% B; 5 min, 100% B; 27 min, 100-70% B (linear); 33 min, 70-50% B (linear); 43 min, 50% B; 44 min, 50-100%B (linear); 60 min, 100% B. Fractions containing 3PG were dried under vacuum and then dissolved in H_2O, methanol, and acetonitrile for further purification using a 2.1 x 100 mm Atlantis Premier Z-HILIC 1.7 μm column (Waters Corporation, Milford, MA, USA). Buffer A was H_2O with 10 mM ammonium acetate pH 9; Buffer B was 10:90 H_2O:acetonitrile with 10 mM ammonium acetate pH 9. The following method was used with 15 μL sample injections at 45 °C with a flow rate of 0.25 mL/min: 0 min, 90% B; 1 min, 90% B; 13 min, 90-60% B (linear); 18.5 min, 60% B; 19 min, 60-90%B (linear); 26 min, 90% B. Fractions containing 3PG were dried under vacuum, dissolved in H_2O, and then an aliquot was diluted to make a solution of H_2O, methanol, and acetonitrile for determination of 3PG concentration. Samples were quantitated on a Waters Acquity H-class UPLC in-line to a Waters SQ Detector 2 mass spectrometer in negative mode, using a 2.1 x 100 mm Atlantis Premier Z-HILIC 1.7 μm column (Waters Corporation, Milford, MA, USA). Buffer A was H_2O with 10 mM ammonium acetate pH 9; Buffer B was 10:90 H_2O:acetonitrile with 10 mM ammonium acetate pH 9. The following method was used with 2 μL sample injections at 45 °C with a flow rate of 0.25 mL/min: 0 min, 90% B; 1 min, 90% B; 13 min, 90-60% B (linear); 18.5 min, 60% B; 19 min, 60-90% B (linear); 26 min, 90% B. Samples were compared to a standard curve of commercial 3PG standard for reference with succinic acid-2,2,3,3-d4 (SAd4) as the internal standard. MS was conducted in negative mode with the following parameters: capillary voltage, 3 kV; selected ion recording (SIR) of SAd4 at 121.04 *m/z*, span of 0.5 Da, dwell time of 0.050 sec, and cone voltage of 26 V; 3-PG at 184.98 *m/z*, span of 0.5 Da, dwell time of 0.150 sec, and cone voltage of 10 V; and MS scan from 20 to 3000 *m/z* with a scan duration of 0.2 sec.

Supplementary Data

Physicochemical analyses of NP-enzyme cluster formation.

Detailed physicochemical analyses/metrology were undertaken to confirm that the enzymes used in experiments did indeed assemble to the NP materials utilized here, to confirm cluster formation, characterize cluster formation, and to also provide some insight into the underlying kinetic mechanisms and channeling properties of the nanoclusters that formed. Most of this analysis focused on the 520 QDs and the 7E Opt 2 ratios unless otherwise indicated. An overview of each analysis is provided below to explain the rationale for undertaking each and the goals desired from that analysis. These include:

(i) ***Geometric estimates of enzyme fitting to the NPs.*** A preliminary estimate of how many of each enzyme could fit around each of the individual NPs was undertaken as described and this information was utilized for estimating the empirical ratios along with being utilized in some of the numerical simulations. This data was also helpful for defining the boundaries that were used in the low and high protein cluster formation of structures analyzed by TEM in **Supplementary Figures 34-35**.

(ii) ***Agarose gel mobility assays***. These were utilized to confirm that each enzyme used in this study did assemble to the NP materials. Gel images from the ratiometric assembly of each enzyme to the 520 QD samples are presented here. Assembly of the enzymes to the other NP materials was confirmed as well with agarose gels but typically with one large ratio on/off the NPs (data not shown). Changes in gel mobility were also used to test for multienzyme assembly to QDs.

(iii) ***PAGE analysis***. Similar to the agarose gel mobility assays undertaken for individual enzyme assembly to the NPLs, polyacrylamide gel electrophoresis (PAGE) analysis was undertaken to show that during the formation of the 7E NPL nanocluster, each of the enzymes within the mixture were indeed being incorporated into the clusters. This analysis was done in an inverse approach were more NPL was added to each sample, the samples allowed to assemble, then loaded and run on a PAGE gel, and finally staining of the gel to reveal how much free protein made it into the gel and was separated *versus* what remained on the cluster and did not enter the gel.

(iv) ***TEM analysis of nanoclusters.*** Several different experiments were undertaken here. Intensive TEM analysis was undertaken of nanocluster formation for each of the NP

materials with the same concentration and ratio of the 7E optimization 2. These are found in manuscript **Figure 4**. Additionally nanocluster size and the ability to control this by altering relative protein ratio was also undertaken; results from this are also shown in manuscript **Figure 4**. The focus of these experiments was to collect enough information from the TEM micrographs to estimate the average nanocluster size dispersion and the number of NPs in each. Where applicable, numbers of samples examined are included with the data.

(v) ***Dynamic light scattering estimating the number of QDs in clusters.*** Dynamic light scattering (DLS) analysis of nanoclusters was also undertaken to provide a different type of analysis where the samples were not dehydrated and static on a grid as in the TEM analysis. This was also meant as a quasi-independent confirmation of the TEM results.

(vi) ***FRET assays estimating the number of QDs in clusters.*** In a similar vein as the above analyses, a FRET assay was undertaken to estimate the average number of QDs that were being incorporated into the 520 QD nanoclusters. This relied on QD-QD donor-acceptor FRET. This approach was also meant to act as a quasi-independent way of corroborating or verifying the data and estimates from the other analyses.

(vii) ***Estimating labeled enzyme incorporation into clusters with FRET.*** In conjunction with the other analyses, a different type of FRET assay was devised and implemented with dye-labeled protein acceptor and using the QDs in the cluster as donors. The object here was to provide an estimate of the percentage of enzyme being incorporated into clusters during formation especially since an excess of enzyme was typically present.

(viii) ***Simulations of nanocluster formation by diffusion limited aggregation.*** As a final contribution to the analyses, we also undertook simulations of nanocluster formation by diffusion limited aggregation (DLA). This helped understand how the clusters actually formed along with providing a plausible mechanism that can help explain their observed size.

Discussion of results from the physicochemical analyses. In drawing conclusions from the foregoing data it is important not to over-interpret the results, but instead to look for generalities, corroboration, and information descriptive of the underlying processes. Firstly, the agarose gel mobility assays confirm that individually the enzymes utilized in this study do indeed assemble on the NP materials v*ia* $(His)_6$ metal-affinity coordination as expected. Moreover, the same type of gel assay suggests that some proportion of all 7 enzymes present in the 7E system also bind to QDs. Although the agarose gel is not able to discern that this assembly produces nanoclusters *via* enzyme cross-linking, this is clearly seen by TEM and DLS. Critically, the DLS analysis also confirms that the nanoclusters do not form when the enzyme is monomeric; multimeric enzymes with their multiple pendant $(His)_6$ are required to effect the crosslinking. We also used agarose mobility shifts to verify assembly of the 7E and 9E (*i.e.*, 7E + Amy/Mal) clusters. For this analysis, the ratios were not those used in the assays but were instead arrived at empirically from the observed mobility shifts. Nevertheless, these results still verify changes to the clusters with each enzyme addition and their presumed incorporation into the cluster. The PAGE analysis also confirms that the enzymes within a 7E mixture are being incorporated into the nanoclusters *albeit* with a slightly different incorporation rate for each. The overall accuracy of these analyses is borne out by their agreement in estimating the average number of 520 QDs incorporated into the nanoclusters with the 7E Opt 2 ratios; specifically, the TEM average was found to be ~5, the DLS average was ~5, and the QD FRET assay average was ~4-7.

In terms of enzyme incorporation into the clusters, though not every combination was tested, overall the FRET incorporation assay consistently reported values around 30-40 % with a minimum value of 16 % (for PGK in the 9E cocktail) and a highest observed value of 72 % (FBA in the 7E cocktail); these values are in line with expectation based on the number of excess enzymes and the surface availability. Importantly, this range of variation suggests that not every cluster that forms will have every enzyme present, a reality not captured in the ideal situations simulated in order to optimize the enzyme ratios. Lastly, the simulations of DLA suggest a plausible mechanism by which the aggregates form and provides a rationale for the factors that may underpin their size and other physicochemical characteristics.

Geometric estimates of enzyme fitting to the NPs. Previous work described a generalized *in silico* methodology for estimating the number of proteins that could fit around a QD.[9] This approach was predicated on the assumption that the proteins are monomeric and only display a single, distal, and available point of attachment to the QD surface. In practice, the attachment is *via* the proteins $(His)_6$-displaying N- or C-termini and its coordination to the ZnS shell on the QD. In this previous study, the fitting was completely determined by the physical size/shape of the protein and how many could fit geometrically around the spherical QD. The validity of predictions made in that study were confirmed by subsequent physicochemical analysis.[9] This same approach was later used to determine that between 10-13 phosphotriesterase enzymes could bind to the surface of a QD of similar size as the 520 QD materials used here (~4.0 nm diameter) through the same $(His)_6$-QD shell Zn coordination.[2]

An important factor in this study was to consider how many of each given enzyme utilized could theoretically fit around the different QD and NPL materials especially when choosing the empirically-determined ratios for the 7E and 4E systems during initial testing. We describe these values (listed in manuscript **Table 1**) as semi-quantitative and used them as guidelines. Upper bound estimates were generated utilizing three geometric approximations, with the intent of utilizing the smallest of the obtained values. Examples are given below for a monomeric (Mal, MW ~74.5 kD), dimeric (Glk, MW ~73.8 kD), and tetrameric (PykA, MW ~214 kD) enzyme. The first step was to determine the minimum radius of the enzyme (r_{minE}), which was based on assuming spherical globular enzymes from the enzyme molecular weight (MW) and optimal packing in the case of dimers and tetramers. The three binding approximations used were: 1) Utilizing the surface area of the QD and the 2.2 nm^2 of surface area occupied per $(His)_6$ motif; we assume enzymes bound through a single $(His)_6$ tag and that all of the residues bind.[1] 2) Considering the area in the immediate vicinity of the QD surface and using the area 2 nm from the QD surface (chosen as the average midpoint of the enzyme), assuming random packing of the enzymes (65% of the area available) and then dividing this value by r_{minE} and rounding down to the lower full integer value. 3) Similar concept as the previous approximation but utilizing the concentric spherical volume from 1.1 nm to 4.6 nm from the QD surface. These distances were chosen as a means of accounting for the CL4 ligand displayed on the QD surface and then a reasonable distance based on the enzyme diameters considering their shapes and mass from PDB

structures, see **Supplementary Figure 13** below. Again, random packing of the enzymes was considered (65% of the volume available) in this part of the estimate.

Supplementary Table 8. Upper bound estimates utilizing geometric approximations of the enzymes that could bind to the different sized QDs.

Enzyme (r_{minE})	$(His)_6$ estimate			Area estimate			Volume estimate		
	520 QD	600 QD	660 QD	520 QD	600 QD	660 QD	520 QD	600 QD	660 QD
**Mal (2.8 nm)**	22	134	256	8	21	34	63	154	235
**Glk (3.5 nm)**	22	134	256	7	29	43	16	39	59
**PykA (5.5 nm)**	22	134	256	6	19	27	13	31	48

The 520-, 600-, and 660 nm emitting QDs have average diameters of ~4.0, 9.7, and 13.4 nm.

The Area estimates in **Supplementary Table 8** provide considerably lower values than the $(His)_6$ and Volume estimates and, as such, we rely more on the values determined with this methodology. We then determined ranges based on the different r_{minE} of the enzymes. As seen below in **Supplementary Table 9**, the range of molecular weights of the enzymes makes it so that the largest monomer (Mal) is on par with the smallest tetramers (FPK), and subsequently the range has considerable overlap

Supplementary Table 9. Approximate range of enzymes that could bind to the different sized QDs.

	520 QD	600 QD	660 QD
**Monomers**	4-12	16-28	26-43
**Dimers**	4-12	14-29	22-43
**Tetramers**	4-10	10-29	16-36

The 520-, 600-, and 660 nm emitting QDs have average diameters of ~4.0, 9.7, and 13.4 nm.

A similar process was utilized to determine fittings for the NPLs. Here, the materials were treated as having just fixed available surface area from the top and bottom for fittings and discounted any ability of the $(His)_6$ tag to coordinate to the edges of the NPLs.

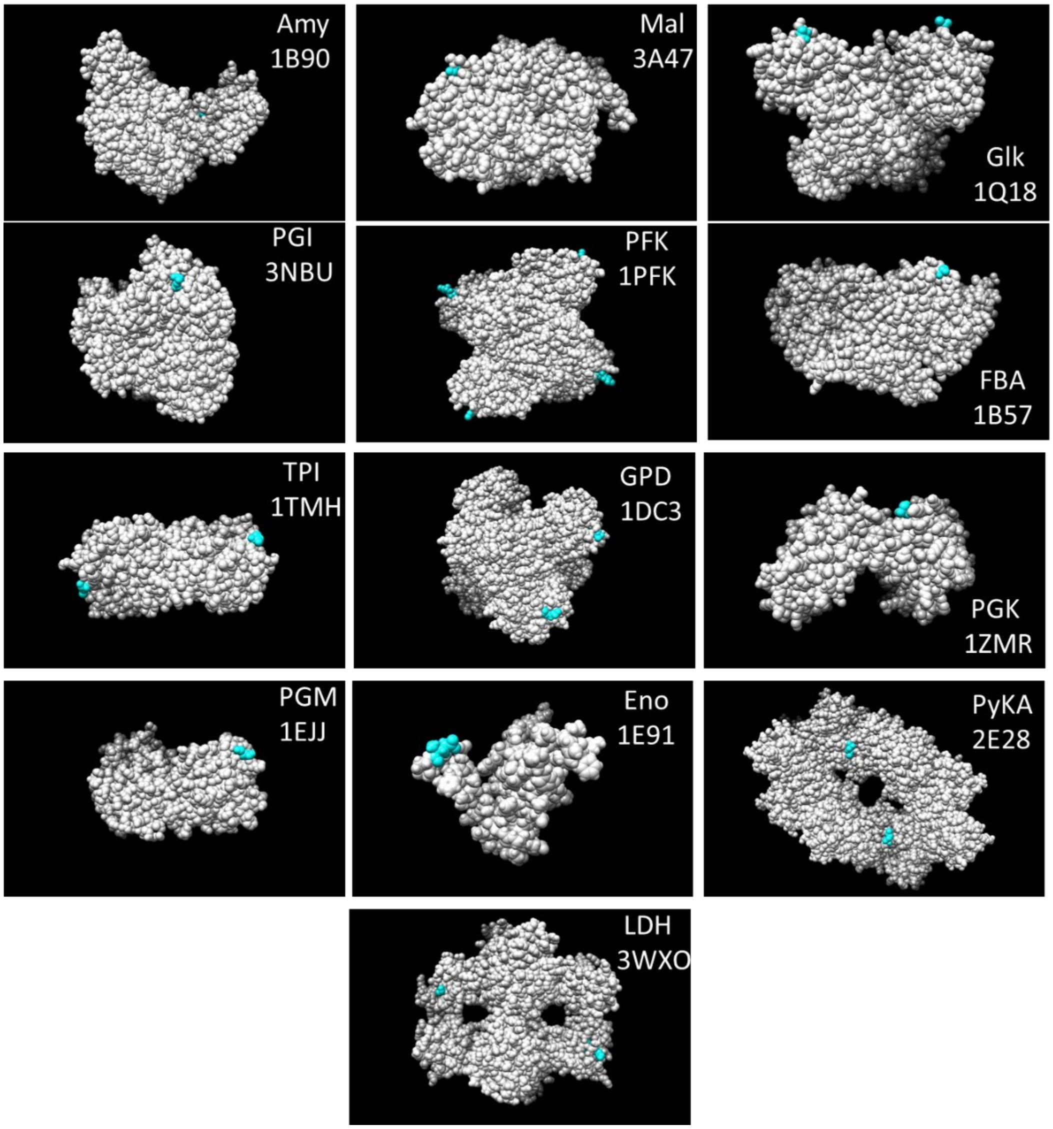

Supplementary Figure 13. PDB-based enzyme structures used for QD fitting analysis. Where visible, the blue residues indicate the point of the pET28 introduced amino acid linker sequence (MGSSHHHHHHSSGLVPRGSH). Structures retrieved from https://www.rcsb.org/.

Agarose gel mobility assays and PAGE analysis.

Agarose gel separation of 520 QDs assembled with increasing ratios of each enzyme utilized in this study were undertaken to confirm that each enzyme did indeed have the ability to coordinate to the surface of the ZnS-overcoated QDs when assembled as nanoconjugates. Low electroendosmosis (EEO) agarose gels were utilized with percentages as indicated in each image set. The percentage agarose in each gel was varied as needed to obtain separation. Gels were imaged on a Biorad Gel Doc XR System. This type of assay is now a common method for confirming that a protein or other molecule such as DNA has indeed assembled to a QD.[2-5, 12, 13, 30-33] 1 Tris/Borate/EDTA (TBE) buffer is 89 mM Tris, 89 mM boric acid, 2 mM EDTA pH 8.3 and was used as is unless otherwise indicated. In some cases, the pH was changed to enhance QD-protein separation. Images were collected at every 5 min or as indicated during separation to show the evolution of mobility differences with time. All gels were replicated at least 2 times independently to verify that the same separation patterns would be obtained. Representative images are shown here.

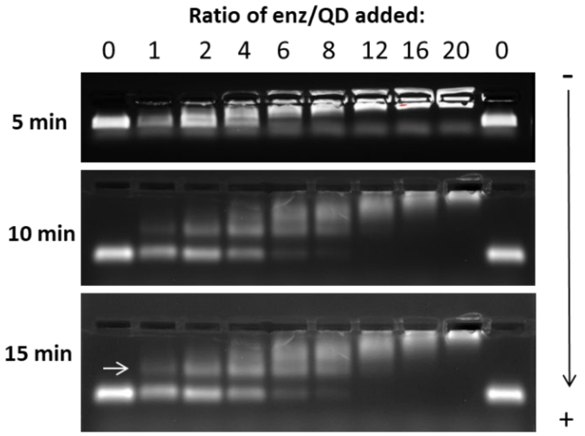

Supplementary Figure 14. Agarose gel mobility assay confirming amylase assembly to 520 nm emitting QDs capped with CL4 ligand. 5 pmol of QD/well in 0.9 % agarose 1×TBE buffer. Arrow indicates putative monovalently-labeled QD. Amylase monomer 59.6 kD. All gels were replicated at least 2 times independently to verify that the same separation patterns would be obtained. Representative images are shown here and below.

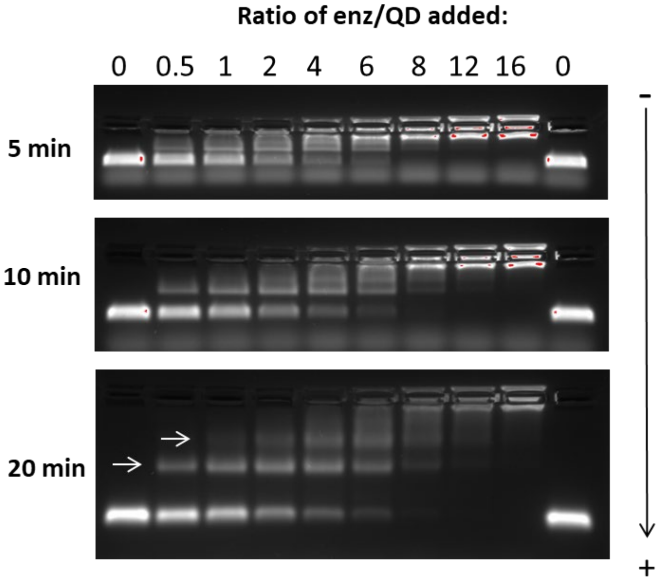

Supplementary Figure 15. Agarose gel mobility assay confirming maltase assembly to 520 nm emitting QDs capped with CL4 ligand. 5 pmol of QD/well in 1.0 % agarose 1×TBE buffer. Arrows indicates putative monovalently- and bivalently-labeled QD. Maltase monomer ~74.5 kD.

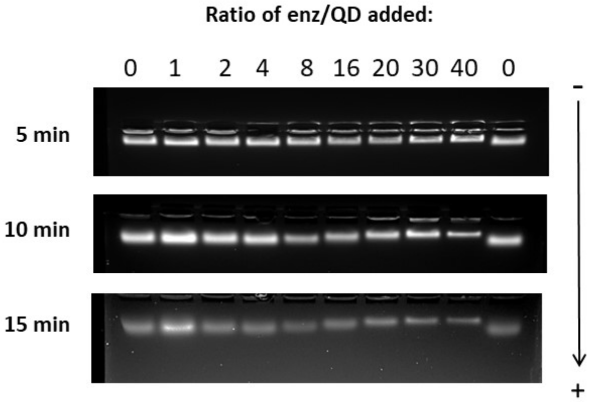

Supplementary Figure 16. Agarose gel mobility assay confirming invertase assembly to 520 nm emitting QDs capped with CL4 ligand. 5 pmol of QD/well in 1.0 % agarose 1×TBE buffer pH 6.45. QD-PGM conjugates did not separate when buffer pH was 8.3 (see **Supplementary Figure 24**) presumably due to the predicted strong negative charge of the protein (> -20). Similarly, the pH of the buffer must be adjusted here to enable migration in the gel. Invertase monomer ~47.2 kD.

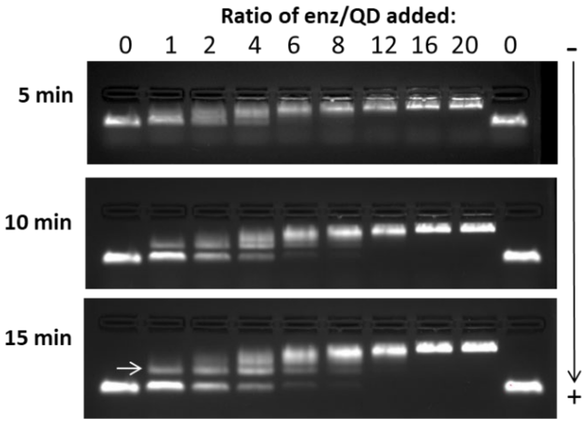

Supplementary Figure 17. Agarose gel mobility assay confirming glucokinase assembly to 520 nm emitting QDs capped with CL4 ligand. 5 pmol of QD/well in 1.5 % agarose 1×TBE buffer. Arrow indicates putative monovalently-labeled QD. Glucokinase monomer 36.9 kD.

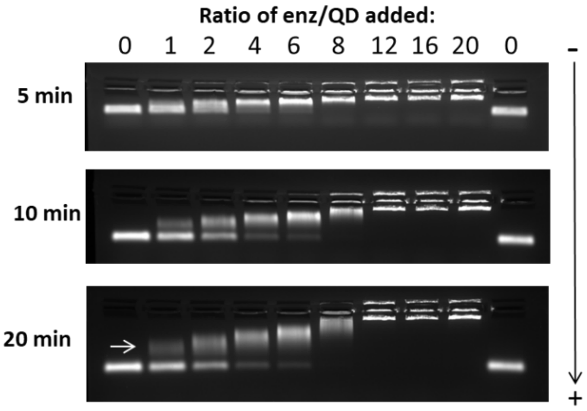

Supplementary Figure 18. Agarose gel mobility assay confirming phosphoglucose isomerase assembly to 520 nm emitting QDs capped with CL4 ligand. 5 pmol of QD/well in 1.5 % agarose 1×TBE buffer. Arrow indicates putative monovalently-labeled QD. Phosphoglucose isomerase monomer 63.7 kD.

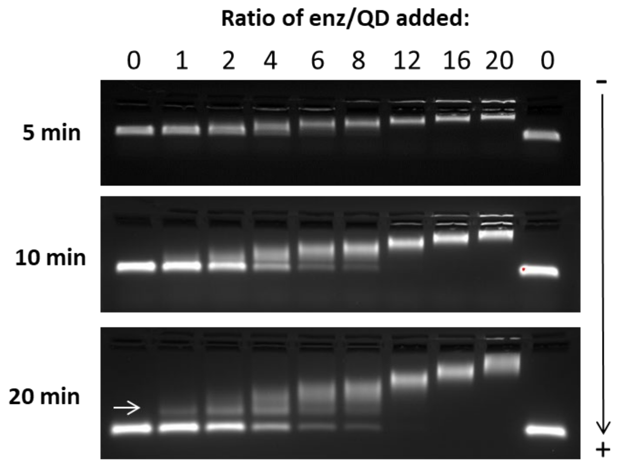

Supplementary Figure 19. Agarose gel mobility assay confirming phosphofructokinase assembly to 520 nm emitting QDs capped with CL4 ligand. 5 pmol of QD/well in 1.5 % agarose 1×TBE buffer. Arrow indicates putative monovalently-labeled QD. Phosphofructokinase monomer 34.6 kD.

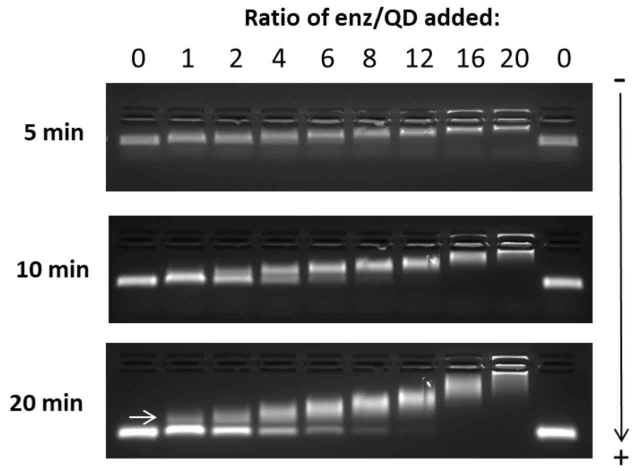

Supplementary Figure 20. Agarose gel mobility assay confirming fructose-bisphosphate aldolase assembly to 520 nm emitting QDs capped with CL4 ligand. 5 pmol of QD/well in 1.5 % agarose 1×TBE buffer. Arrow indicates putative monovalently-labeled QD. Fructose-bisphosphate aldolase monomer 41.3 kD.

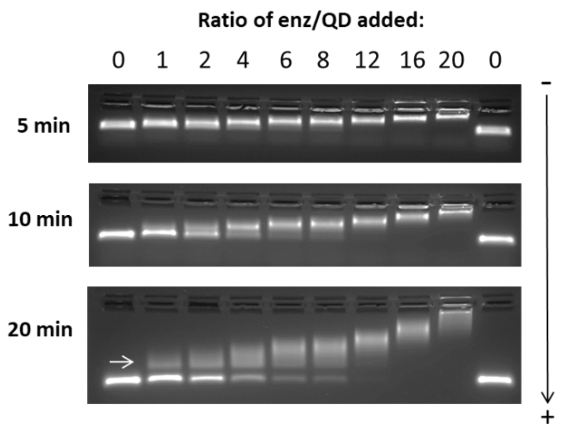

Supplementary Figure 21. Agarose gel mobility assay confirming triose phosphate isomerase assembly to 520 nm emitting QDs capped with CL4 ligand. 5 pmol of QD/well in 1.5 % agarose 1×TBE buffer. Arrow indicates putative monovalently-labeled QD. Triose phosphate isomerase monomer 29.1 kD.

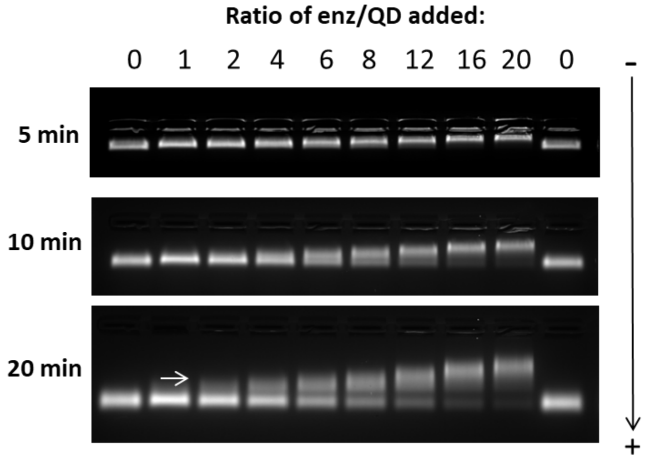

Supplementary Figure 22. Agarose gel mobility assay confirming glyceraldehyde-3-phosphate dehydrogenase assembly to 520 nm emitting QDs capped with CL4 ligand. 5 pmol of QD/well in 1.5 % agarose 1×TBE buffer. Arrow indicates putative monovalently-labeled QD. Glyceraldehyde-3-phosphate dehydrogenase monomer 37.7 kD.

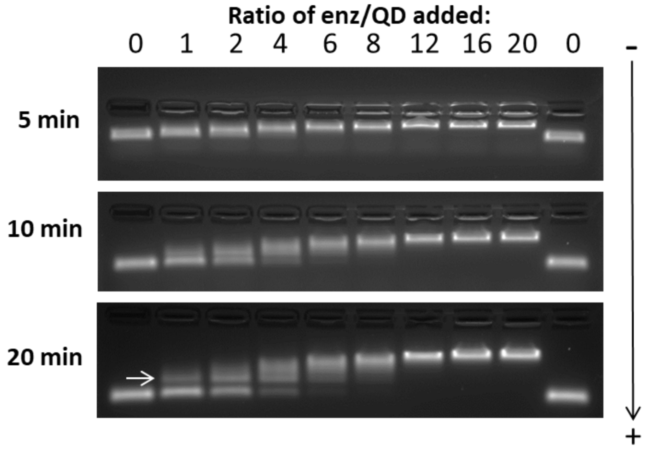

Supplementary Figure 23. Agarose gel mobility assay confirming phosphoglycerate kinase assembly to 520 nm emitting QDs capped with CL4 ligand. 5 pmol of QD/well in 1.5 % agarose 1×TBE buffer. Arrow indicates putative monovalently-labeled QD. Phosphoglycerate kinase monomer 43.3 kD.

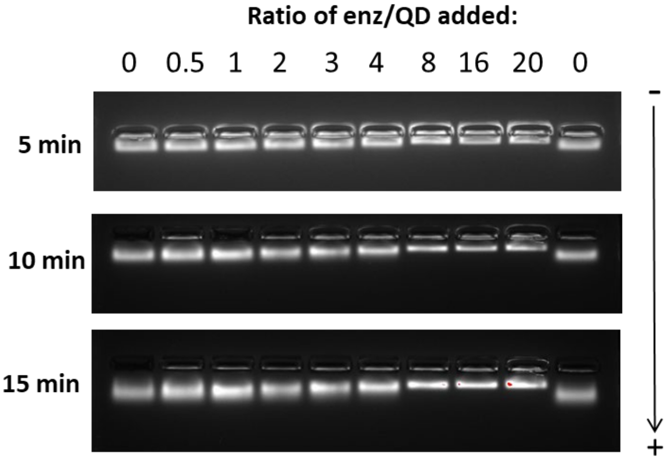

Supplementary Figure 24. Agarose gel mobility assay confirming phosphoglucomutase assembly to 520 nm emitting QDs capped with CL4 ligand. 5 pmol of QD/well in 1.0 % agarose 1×TBE buffer pH 6.78. QD-PGM conjugates did not separate when buffer pH was 8.3 presumably due to the predicted strong negative charge of the protein (> -20). Phosphoglucomutase monomer 58.3 kD.

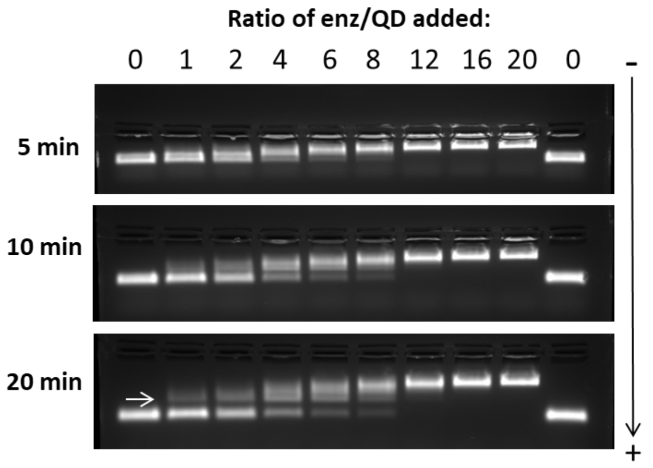

Supplementary Figure 25. Agarose gel mobility assay confirming enolase assembly to 520 nm emitting QDs capped with CL4 ligand. 5 pmol of QD/well in 1.5 % agarose 1×TBE buffer. Arrow indicates putative monovalently-labeled QD. Enolase monomer 47.8 kD.

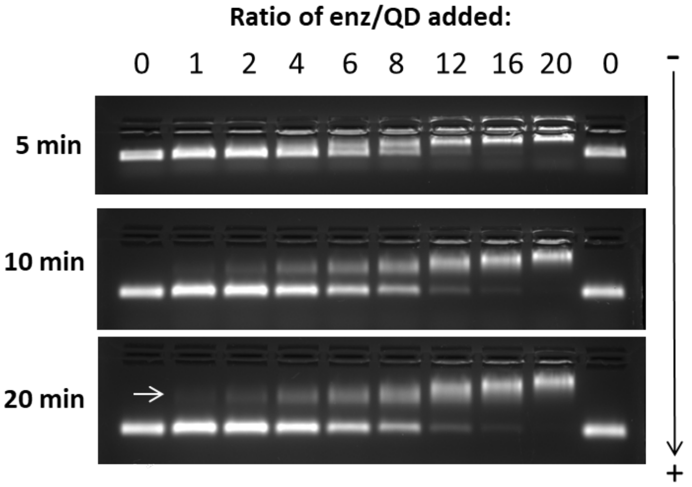

Supplementary Figure 26. Agarose gel mobility assay confirming pyruvate kinase assembly to 520 nm emitting QDs capped with CL4 ligand. 5 pmol of QD/well in 1.5 % agarose 1×TBE buffer. Arrow indicates putative monovalently-labeled QD. Pyruvate kinase monomer 53.5 kD.

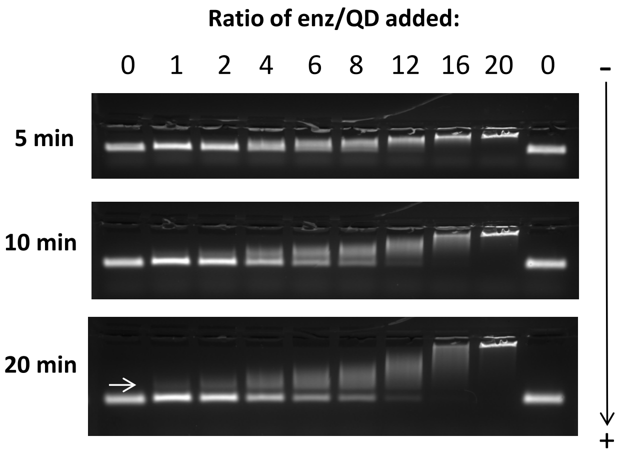

Supplementary Figure 27. Agarose gel mobility assay confirming lactate dehydrogenase assembly to 520 nm emitting QDs capped with CL4 ligand. 5 pmol of QD/well in 1.5 % agarose 1×TBE buffer. Arrow indicates putative monovalently-labeled QD. Lactate dehydrogenase monomer 38.7 kD.

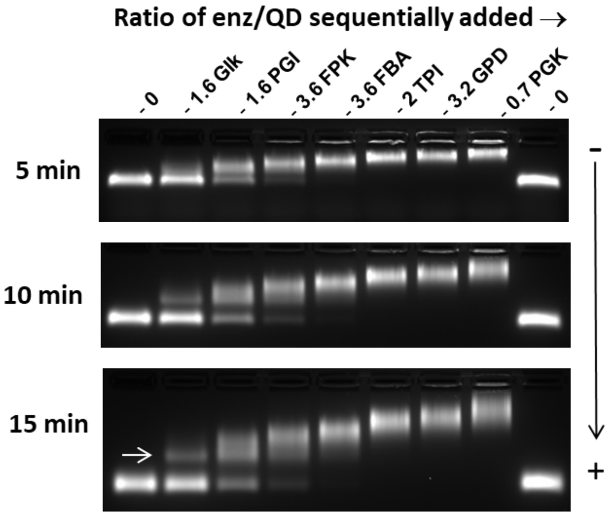

Supplementary Figure 28. Agarose gel mobility assay confirming assembly of 7 enzymes to 520 nm emitting QDs capped with CL4 ligand. 7.5 pmol of QD/well in 1.5 % agarose 1×TBE buffer. Enzymes added to QDs and their ratio per QD shown as they were sequentially added to samples for assembly. These ratios are different from those used in the catalytic assays and were arrived at empirically to reveal changes in mobility during electrophoresis with each sequential enzyme addition.

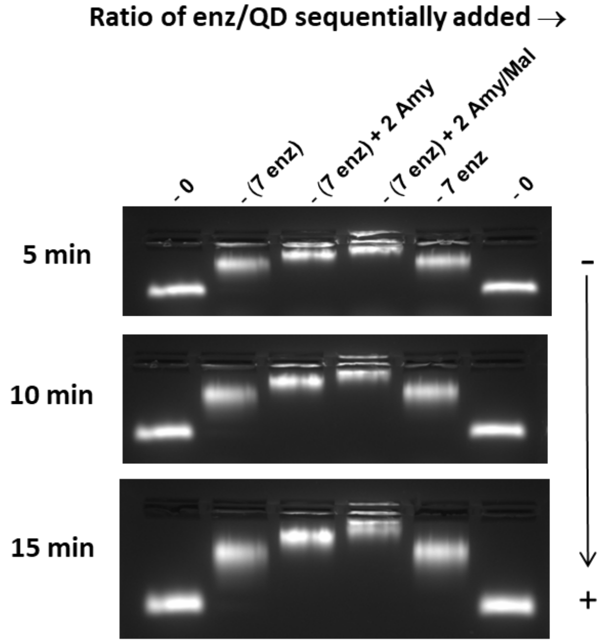

Supplementary Figure 29. Agarose gel mobility assay confirming assembly of 9 enzymes to 520 nm emitting QDs capped with CL4 ligand. 7.5 pmol of QD/well in 1.5 % agarose 1×TBE buffer. 7 enzyme ratios as in gel above. Enzymes added to QDs and their ratio per QD shown as they were sequentially added to samples for assembly. These ratios are different from those used in the catalytic assays and were arrived at empirically to reveal changes in mobility during electrophoresis with each sequential enzyme addition.

PAGE analysis:

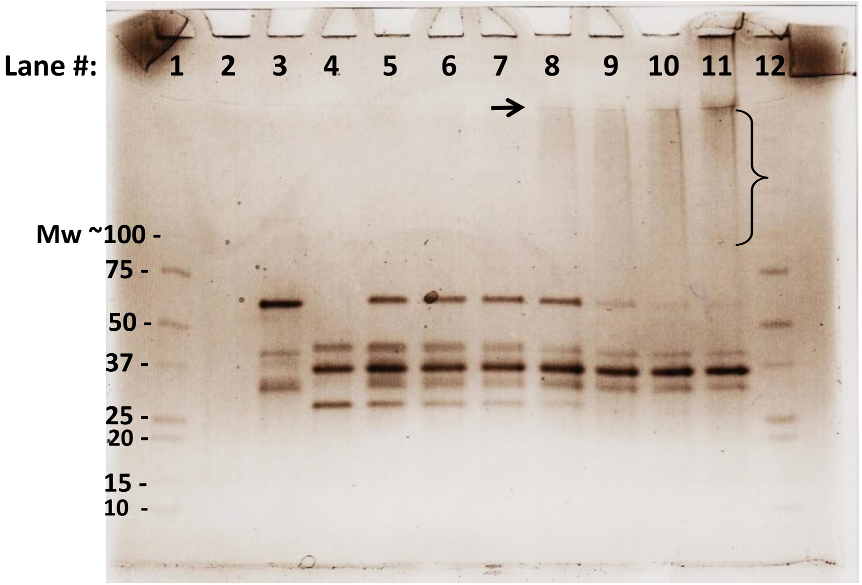

Gel Contents:

Lane 1 and Lane 12: Protein marker
Lane 2: NPL 13 pmol
Lane 3: Glk (36.9 kD monomer) PGI (63.7 kD dimer) PFK (34.6 kD mono) PGK(43.3 kD mono)
Lane 4: FBA (41.3 kD monomer) TPI (29.1 kD monomer) GPD (37.7 kD monomer)
Lane 5: 7 proteins 25 pmol each (no NPLs)
Lane 6: 7 proteins 25 pmol each + NPL 0.33 pmol
Lane 7: 7 proteins 25 pmol each + NPL 0.65 pmol
Lane 8: 7 proteins 25 pmol each + NPL 1.13 pmol
Lane 9: 7 proteins 25 pmol each + NPL 3.25 pmol
Lane 10: 7 proteins 25 pmol each + NPL 6.5 pmol
Lane 11: 7 proteins 25 pmol each + NPL 13 pmol

Supplementary Figure 30. PAGE analysis of NP-enzyme clusters. Representative 10% PAGE gel stained with commassie blue showing the results of preassembling a mixture of the 7 E pathway with increasing amounts of NPL. Protein depletion as a function of increasing NPL concentration is used here as evidence of cluster formation. Note how increasing NPL presence removes available protein from the main area of the gel between *ca*. 25 and 75 kD molecular weight as shown by the loss of intensity of each protein band as a function of increasing NPL. Simultaneously, there is an increasing amount of higher molecular weight staining (>100 kD) and also the appearance of a new dark band at the intersection of the loading and separating gels as indicated by the arrow. This serves as part of the evidence that multiple enzymes in the mixture coordinate to the NPLs as they form nanoclusters. Nanoclusters are not predicted to enter into the separating portion of the gel due to previous experience with these materials, rather they are expected to remain static due to large size and or precipitation in the gels. It is also probable that enzymes that are somewhat 'loosely' associated with the clusters may get stripped from the nanocluster as they separate in the gel. If so, this would suggest that the actual amount of enzyme in the cluster is larger. As each enzyme has different affinity for the crosslinked NPLs, they may not be stripped from the sample at the same rate. Protein sizes given for lane 3, 4 correspond to what the given proteins MW in the gel appears as with Glk, PFK, FBA, TPI, and GPD running as monomers.

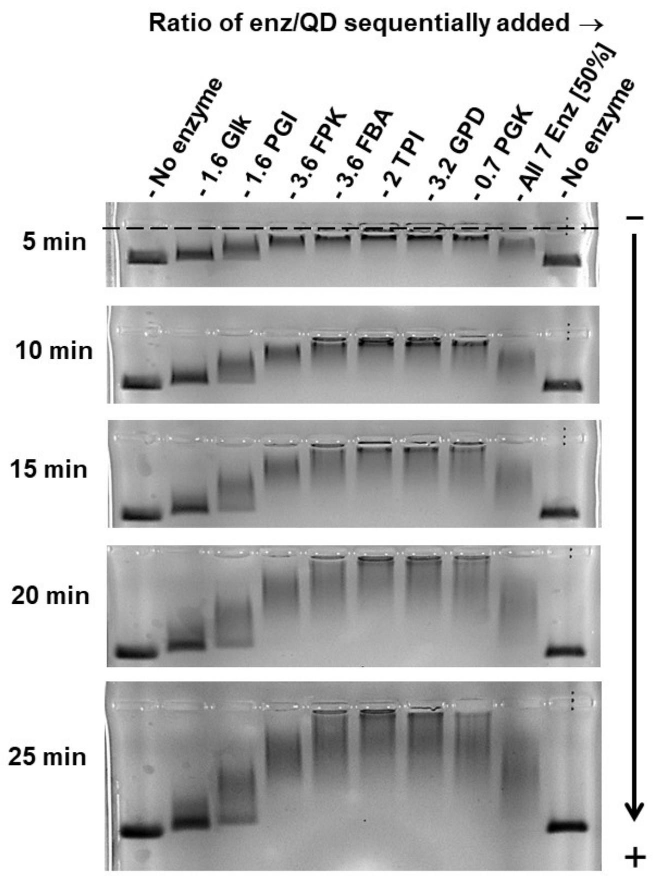

Supplementary Figure 31. Agarose gel mobility assay confirming assembly of 7 enzymes to 5 nm diameter AuNPs capped with 50% thioctic acid (TA) / 50% TA-nitrilotriacetic acid (NTA). 10 pmol of AuNP/well in 0.85% agarose 1×TBE buffer. Enzymes added to the AuNPs and their ratio per NP shown as they were sequentially added to samples for assembly. All 7 Enz [50%] sample has all 7 enzymes identical to the lane to its left but at 50% concentration. The black dashed line in the 5 min image indicates the location of the sample wells. These ratios are different from those used in the catalytic assays and were arrived at empirically to reveal changes in mobility during electrophoresis with each sequential enzyme addition.

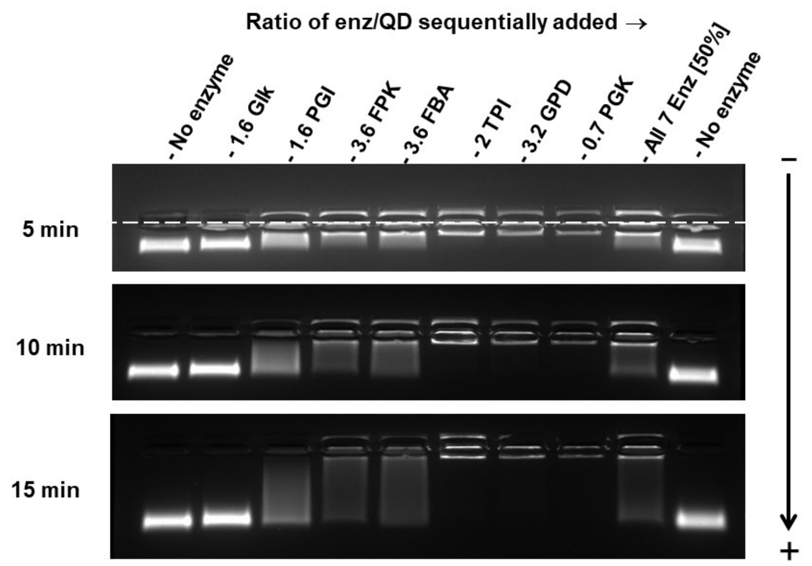

Supplementary Figure 32. Agarose gel mobility assay confirming assembly of 7 enzymes to commercial 525 nm emitting QDs capped with ITK-carboxyl ligands. 2.5 pmol of QD/well in 1.25 % agarose 1×TBE buffer. Enzymes added to QDs and their ratio per QD shown as they were sequentially added to samples for assembly. All 7 Enz [50%] sample has all 7 enzymes identical to the lane to its left but at 50% concentration. The white dashed line in the 5 min image indicates the location of the sample wells. These ratios are different from those used in the catalytic assays and were arrived at empirically to reveal changes in mobility during electrophoresis with each sequential enzyme addition. Addition of the last 3 enzymes resulted in almost no mobility presumably due to aggregate size. Dropping the concentration of all 7 enzymes by half showed a band coming back into the gel but migrating slightly slower than the unassembled QDs.

PAGE analysis:

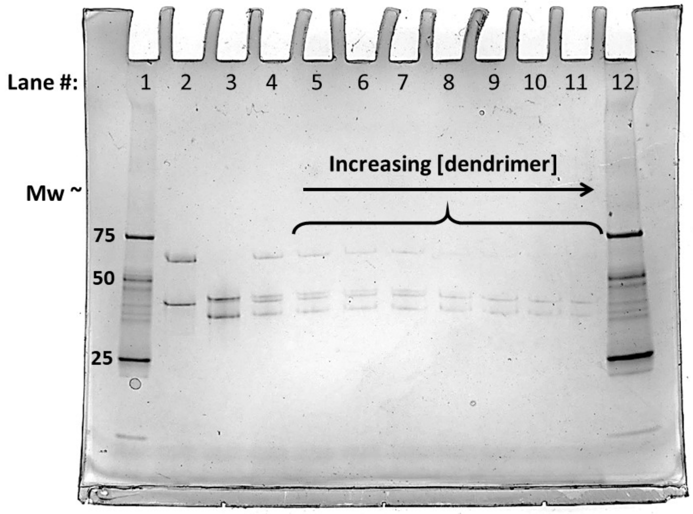

Gel Contents:
Lane 1 and Lane 12: Protein marker
Lane 2: Glk (36.9 kD monomer) PGI (63.7 kD dimer) PFK (34.6 kD mono) PGK (43.3 kD mono)
Lane 3: FBA (41.3 kD monomer) TPI (29.1 kD monomer) GPD (37.7 kD monomer)
Lane 4: 7 proteins 5 pmol each (no dendrimer)
Lane 5: 7 proteins 5 pmol each + dendrimer 0.3 pmol
Lane 6: 7 proteins 5 pmol each + dendrimer 0.7 pmol
Lane 7: 7 proteins 5 pmol each + dendrimer 1.3 pmol
Lane 8: 7 proteins 5 pmol each + dendrimer 2.5 pmol
Lane 9: 7 proteins 5 pmol each + dendrimer 5 pmol
Lane 10: 7 proteins 5 pmol each + dendrimer 10 pmol
Lane 11: 7 proteins 5 pmol each + dendrimer 20 pmol

Supplementary Figure 33. of 50x Ni^{2+} supplemented bis-MPA-COOH dendrimer. Protein depletion as a function of increasing dendrimer concentration is used here as evidence of cluster formation. Note how increasing dendrimer presence removes available protein from the main area of the gel between *ca*. 25 and 75 kD molecular weight as shown by the loss of intensity of each protein band as a function of increasing dendrimer. Due to the presumed fragility of the dendrimer protein clusters, they were not preheated prior to loading on the gel and the loading buffer was non-reducing with no SDS or urea added in. Hence, in comparison to **Supplementary Figure 30** above, some of the proteins comigrate. This serves as evidence that multiple enzymes in the mixture coordinate to the dendrimers as they form nanoclusters. Nanoclusters are not predicted to enter into the separating portion of the gel due to previous experience with these materials. Rather they are expected to remain static due to large size and or precipitation in the gels. It is also probable that enzymes that are somewhat 'loosely' associated with the clusters may get stripped from the nanocluster as they separate in the gel. If so, this would suggest that the actual amount of enzyme in the cluster is larger. As each enzyme has different affinity for the crosslinked NPLs, they may not be stripped from the clusters in the sample at the same rate.

TEM analysis of nanoclusters (QD ratio *versus* nanocluster size). Transmission electron microscopy (TEM) imaging of the NPs and NP-enzyme clusters were performed essentially as described in refs.[17, 34-36] The enzyme cocktail of interest was prepared from stock solutions and passed through 0.45 µm filters. The QD solution was sonicated and vortexed for ~3 min and passed through 0.45 µm filters. The NPL solution was sonicated and vortexed for at minimum 5 min. The enzyme and NP solutions were then mixed at the desired ratios, creating samples of 50 µl in 250 mM HEPES buffer with NP concentrations ranging from 10-150 nM depending on the sample. After mixing, the samples were allowed to conjugate at RT for >1 hour before placing on TEM grid, followed by washing, and drying. Three of the TEM experiments including those shown in manuscript Figure 4 a,b and d,e were replicated and returned essentially the same distributions as shown. All other data was collected from a single set of samples.

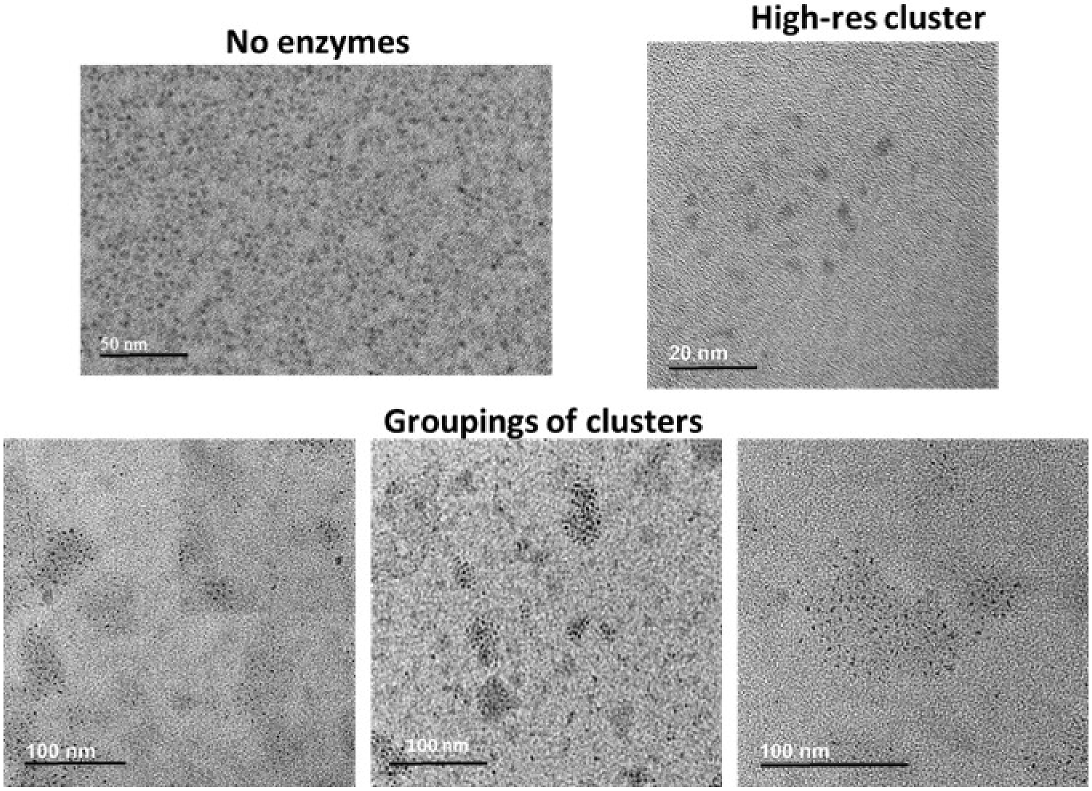

Supplementary Figure 34. Representative images of 520 nm QDs capped with CL4 assembled with the 7 enzyme system at Opt 2 ratios. Note the difference in clustering when enzymes are not present. The 520 nm emitting QDs have a diameter of 4.0 ± 0.4 nm.

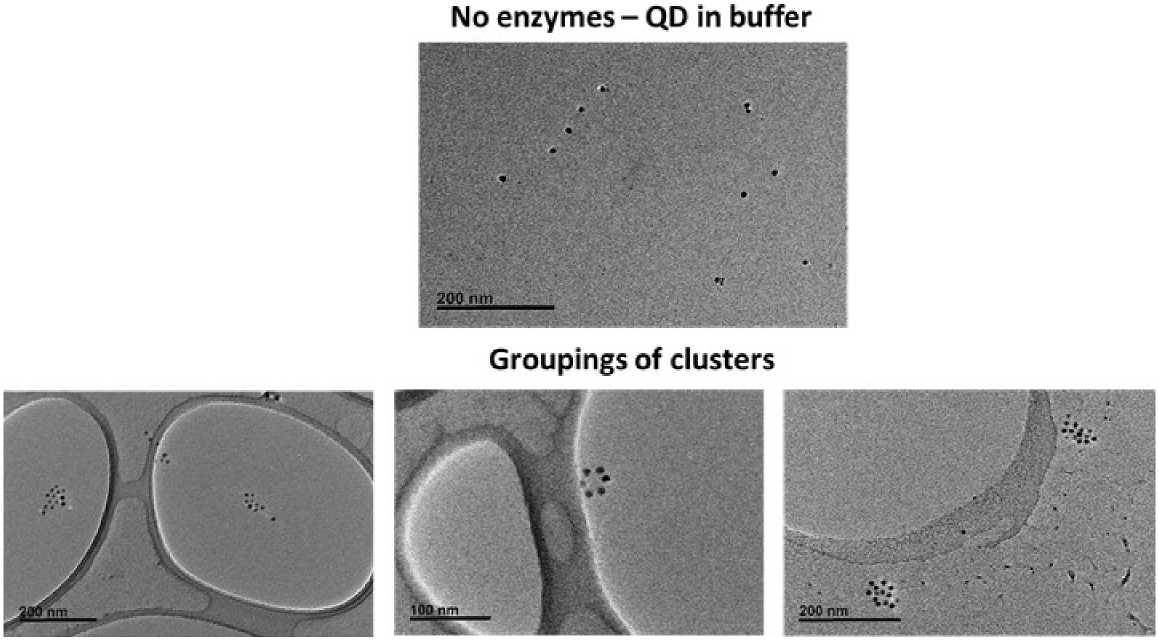

Supplementary Figure 35. Representative images of 600 nm emitting QDs capped with CL4 assembled with the 7 enzyme system at Opt 2 ratios. Note the difference in clustering when enzymes are not present. The 600 nm emitting QDs have a diameter of 9.7 ± 1.0 nm.

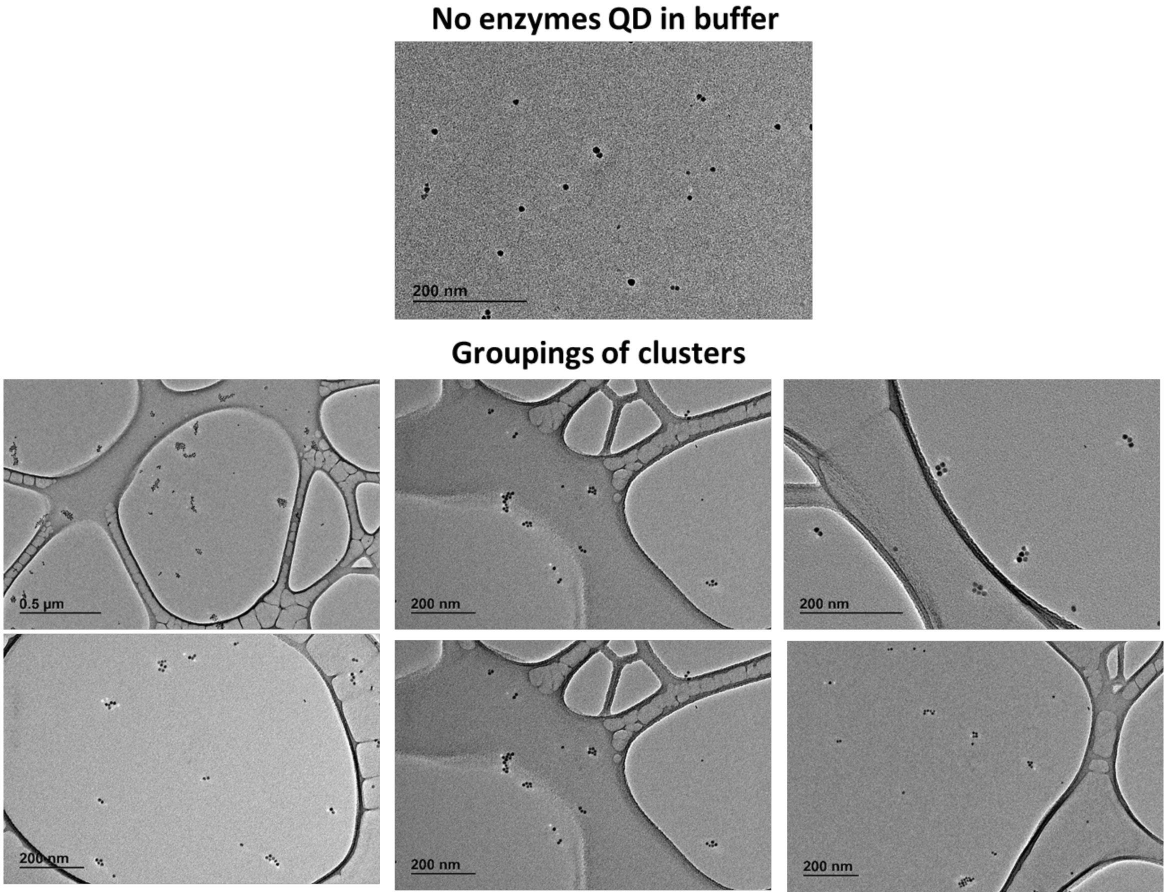

Supplementary Figure 36. Representative images of 600 nm emitting QDs capped with CL4 assembled with the 9 enzyme system that processes maltotetraose or maltoheptaose to 3-phosphoglycerate. Note the difference in clustering when enzymes are not present. The 600 nm emitting QDs have a diameter of 9.7 ± 1.0 nm. Ratios are as in **Supplementary Table 1**.

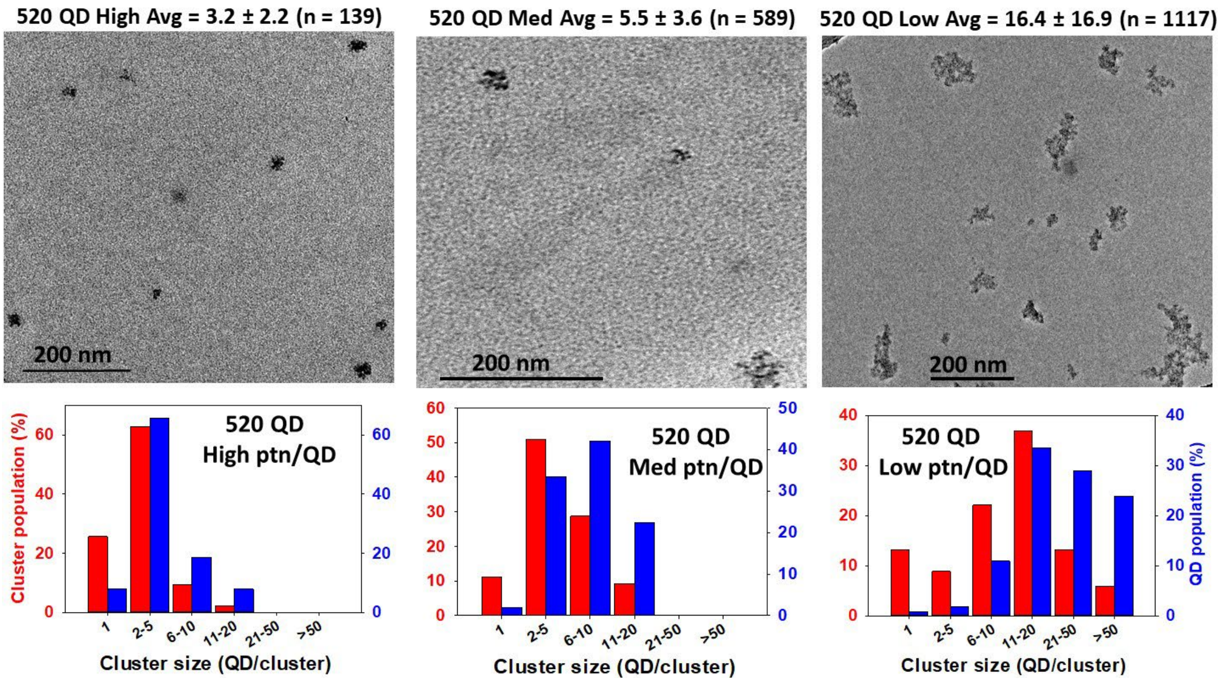

Supplementary Figure 37. TEM characterization of QD-enzyme clusters. Representative TEMs of 520 QDs assembled with the 7 enzyme system (Opt 2 ratios relative to each other) at high, medium (Med), and low relative overall protein concentration per NP. Ratios of protein per QD can be found in **Supplementary Table 2**. Average cluster size is given above the micrograph along with the number of QDs counted. Corresponding bar plots for each sample below showing the distribution of cluster sizes present (red) and number of QD per cluster size (blue).

At high protein ratio per 520 QD, small clusters with an average size of ~3.2 QD/cluster are observed. At low protein ratios, cluster size increases *ca*. 5 fold to ~16.4 QD/cluster. The relative ratio/concentration of protein for the 520 QDs shown in Manuscript **Figure 4A** are intermediate to that used for **Supplementary Figure 37**, and, consistent with this, the average cluster size of ~5.5 also falls between the two average high and low protein/QD values noted above. The plots in **Supplementary Figure 37** tabulate the cluster configurations showing most of the 520 QDs in small cluster sizes of 1-5 at high protein and then assembling into predominantly larger clusters at low protein.

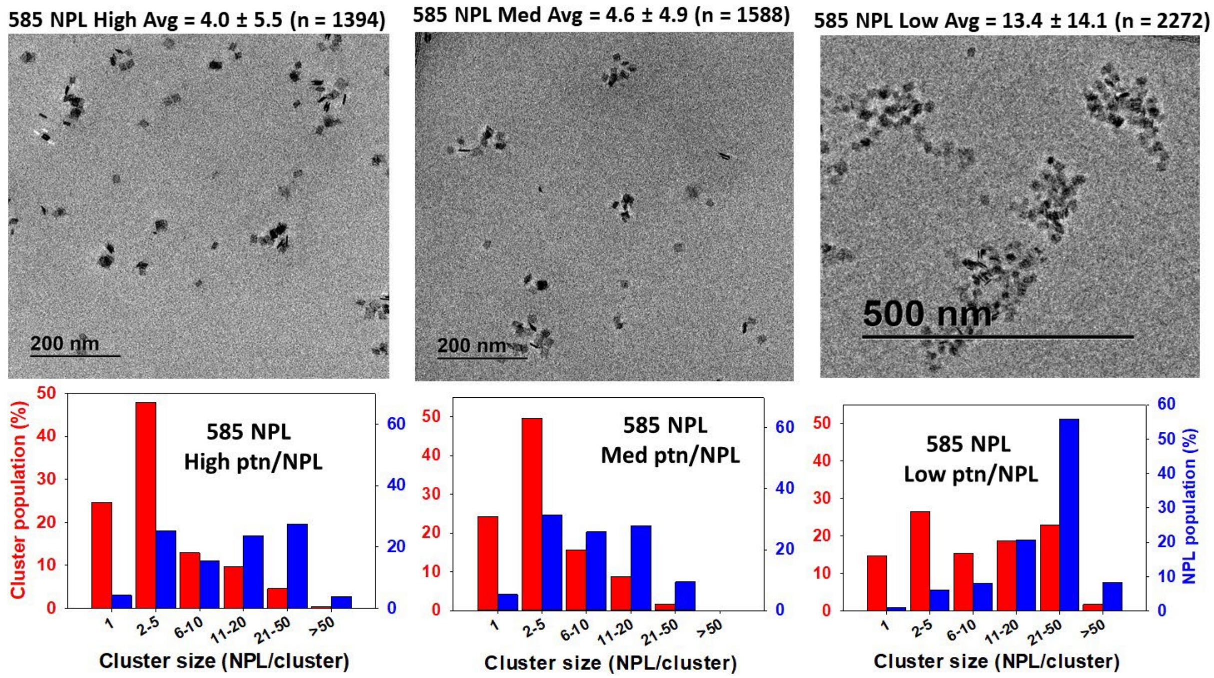

Supplementary Figure 38. TEM characterization of QD-enzyme clusters. Representative TEMs of 585 NPL assembled with the 7 enzyme system (Opt 2 ratios relative to each other) at high, medium (Med), and low relative overall protein concentration per NP. Ratios of protein per NPL can be found in **Supplementary Table 2**. Average cluster size is given above the micrograph along with the number of QDs counted. Corresponding bar plots for each sample below showing the distribution of cluster sizes present (red) and number of NPLs per cluster size (blue).

For the NPLs, high protein ratio yields average sizes of ~4 NPL/cluster which increases 3-fold to 13.4 with increased NPLs and the switch to low protein concentration/NPL. A similarly complex change in distribution profile is observed for the NPLs with change in protein ratio/concentration.

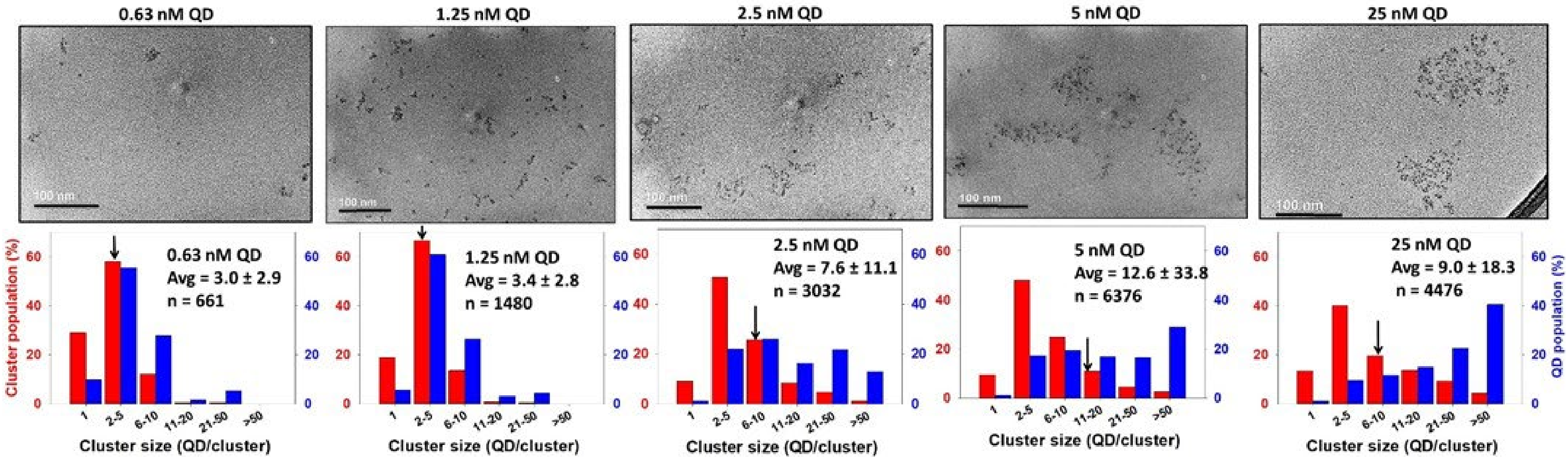

Supplementary Figure 39. TEM characterization of QD-enzyme clusters from Manuscript Figure 4C-E. Top - Representative TEMs of 520 QDs assembled with the 7 enzyme system (Opt 2 ratios with enzyme concentration fixed (Glk 5.5, PGI 1, PFK 9, FBA 12, TPI 1, GPD 27, PGK 7.5 nM) as assembled with the indicated concentrations of QD. Bottom - bar plots with corresponding cluster analysis and average cluster size (black arrow) and number of QDs counted given.

Dynamic light scattering estimating the number of QDs in clusters. Dynamic light scattering (DLS) studies were undertaken as part of the characterization of the nanoclustered systems and to support the TEM results with solution-based measurements. The DLS system utilized is described in refs.[11, 37] Individual components, *i.e.* 520 QDs, buffers, and enzymes were passed through 0.2 μm syringe filters before combining at the respective ratios, preparations were allowed to bind for a minimum of 90 min and then measured. Final concentrations of QDs were in the range of 10 nM. In addition to the 7E cocktail (QD + 7E at Opt 2 ratios), we measured QDs coated only with PGK (monomer) or GPD (tetramer) as control systems. In **Supplementary Figure 40**, the results are presented (note that the diameters are presented in log scale). The monomeric PGK increased the hydrodynamic diameter (D_H) of the QD but was unable to crosslink the QDs to create clusters. In contrast, both the 7E cocktail and the multimeric GPD showed considerable increase in the size of the assembled clusters. ***This, in and of itself, is a powerful control result and strong evidence supporting the concept that the multimeric enzymes are the critical element that serves to cross-link the QDs giving rise to higher order clusters***. For the clustered systems, the D_H only approximately doubled in size (**Supplementary Table 10**), yet the peaks are much broader, with values out as far as 45 nm for the upper limit. We highlight that the DLS measurements assumed spherical shapes and as such QD dimers would not necessarily result in doubling of the D_H. Estimates of the cluster size were obtained by dividing the larger volume by the smaller QD only volume; we take into account a random packing distribution that would allow only 65% of the larger volume as accessible. Volumes were obtained by fitting the DLS data (lines in **Supplementary Figure 40**) with modified Gaussian curves that account for the extended tails.

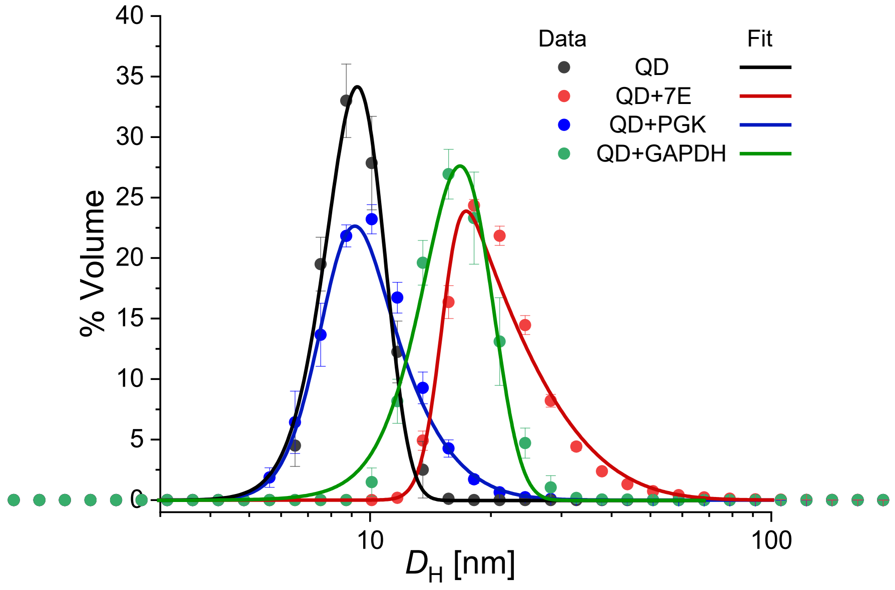

Supplementary Figure 40. DLS data of 520 QDs and the change in D_H upon addition of enzymes and enzyme cocktails. Fits are modified Gaussian curves to account for extended tails. All data along with the error are from results with a minimum of n = 3 separate sample run with 10 measurements each.

Supplementary Table 10. DLS data and estimates of QD cluster sizes.

Sample	D_H [nm]	Average QDs per cluster [a] / [range] [b]
QD	8.5 ± 1.5	-
QD PGK	9.5 ± 2.0	-
QD GAPDH	17 ± 4.0	5 / [4 - 15]
QD+7E	16 ± 10.0	5 / [2 - 22]

[a] Averaged values were obtained by dividing the reported cluster volume obtained by the fitted D_H (accounting for the random packing distribution) by the QD D_H. [b] The range of cluster sizes was estimated as above but considering the uncertainty of the D_H values.

FRET assays estimating the number of QDs in clusters. FRET experiments were realized in conjunction with the DLS experiments as an additional estimation of the average number of QDs being incorporated into the QD-enzyme nanoclusters. Small amounts (3% or 5%) of a slightly larger QD with a slight emission shift (from 520 nm to 545 nm) were added to a typical cluster formation. The supposition behind this approach was that if the clusters are formed and incorporate them, the larger QD will act as a FRET acceptor to the smaller 520 QD (520 QD → 540 QD Förster distance or R_0 = 6.9 nm, while the 520 QD → 520 QD homoFRET R_0 = 6.3 nm).[38-40] The FRET is observed as a decrease in the 520 nm emission if the clusters are indeed forming. To estimate the cluster formation, some working assumptions must be made including: 1) The 540 QDs do not modify the underlying cluster formation; and 2) the 520 QD homoFRET allows for infinite nearest neighbors. We then estimated FRET values based on the size of the clusters. Here, we assumed that only clusters that have an acceptor will be quenched and this is dependent on the fraction of acceptor 540 QD (A%) as well as on the cluster size, *i.e.* the number of QDs in a cluster.[41] The FRET efficiency (E_{FRET}) is then determined by estimating the Poisson probability of any cluster having at least 1 acceptor (which will result in 520 QD quenching) based on the A% and cluster size:

$$E_{FRET} = P(k > 0; \lambda = \frac{A\%}{QDs\ in\ cluster}) \qquad \text{(Supplementary Eq. 4)}$$

Where k > 0 signifies the presence of an acceptor in the cluster and the expected result (λ) is determined by dividing the A% by the size of the cluster. The results for QD with 7E and 9E cocktails are presented in **Supplementary Figure 41**. The lines are the estimated values with the data presented as the boxes with the uncertainty arising from repeats.

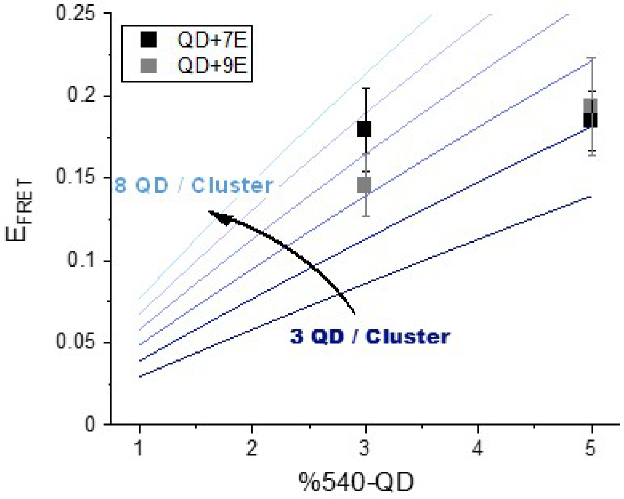

Supplementary Figure 41. FRET efficiency as a function of the percentage of 540 QD acceptor added to a cluster formation experiment. Lines represent the estimated FRET efficiency going from 3 QDs per cluster (dark blue) to 8 QDs per cluster (light blue). Data for the QDs with 7E cocktail are black squares and QDs with 9E cocktails are grey squares. Data shown is the mean from n = 3 independent experimental samples ± standard deviation.

Though the uncertainty of the values and required assumptions make this analytical strategy and its results more qualitative than quantitative, the results still clearly demonstrate that cluster formation was occurring. Furthermore, the obtained values were in line with the average cluster size reported by the TEM and DLS, specifically for QD + 7E with 4-7 QDs per cluster and QD + 9E with 4-6 QDs per cluster. Thus, these FRET assays provided a third independent methodology that demonstrated and supported the reported cluster sizes.

Estimating labeled enzyme incorporation into clusters with FRET. As the enzymatic assays were almost always realized with an excess amount of enzyme to available NP surface, unless multi-layer enzyme systems were forming, it was anticipated that a fraction of the enzymes would be free in solution. To quantify the amount of enzyme bound to the QD clusters we utilized FRET once more. Again due to the 520 QD having the smallest radius and as such the larger FRET efficiency, the 520 QD was chosen as the NP for this characterization. In these assays, the enzyme to be studied was labeled with Cy3 dyes (Cy3 monofunctional reactive dye, GE Healthcare) in excess and purified to eliminate any free dye.[1] UV-Vis absorption spectroscopy was used to quantify the average number of dye labels attached per enzyme and showed that these ranged from 3-10 dyes per enzyme. We utilized these purified enzymes to create FRET calibration curves in which the increasing FRET within the QD-enzyme assembly correlated with increasing enzyme concentration present, see **Supplementary Figure 42**.[1] These curves assume the centrosymmetric distribution of the Cy3 around the QD remains valid through the enzyme range and in the presence of any QD clusters that may have formed.[38]

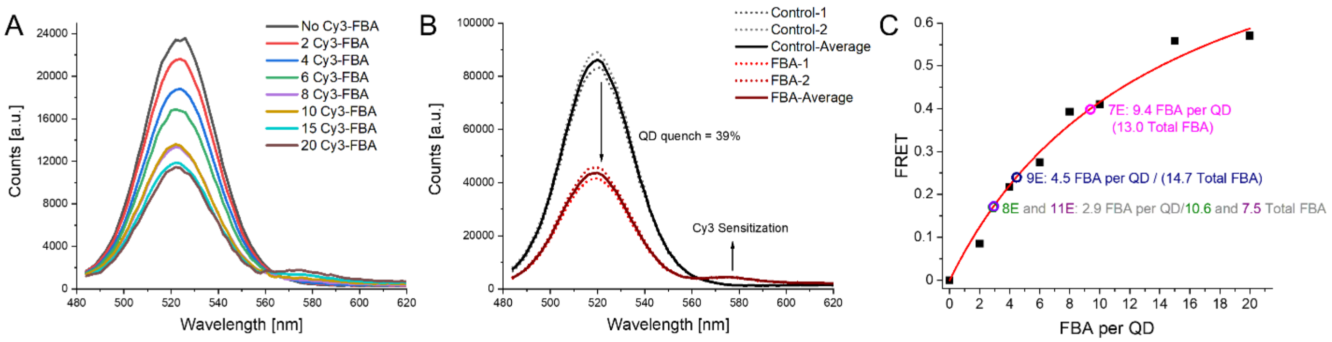

Supplementary Figure 42. Spectral characterization to determine number of bound enzymes in QD clusters. Samples were always run at least in duplicates. **(A)** FRET calibration curve of 520 QD with increasing number of Cy3-labeled FBA enzymes. **(B)** The 7E QD cluster was formed either with a 7E cluster containing no labeled FBA (black) or with Cy3-labeled FBA (red). Dotted lines are the original repeats with the full lines showing the average. **(C)** The FRET calibration curve from A is shown as black squares and the corresponding FRET fit is the red line. FRET efficiency gathered from the QD quenching as seen in panel B are then graphed on the fit to calculate the number of FBA per QD.

For these analyses, the enzyme directed QD clusters was subsequently prepared by replacing the pertinent enzyme with the labeled version of the enzyme, all other enzymes remained unlabeled. In the first example seen in **Supplementary Table 11**, the QD and 7E cocktail was mixed and allowed to bind for 90 minutes before the fluorescent spectra were obtained. A fully unlabeled 7E cocktail was used for the normalized QD emission and the FRET efficiency in the cluster could then be correlated to the calibration curve and the number of enzyme-Cy3 present per QD, see **Supplementary Figure 42**.

Supplementary Table 11. Enzyme incorporation into QD clusters in the 7E system.

Enzyme	Experimental E-Cy3 bound to QD cluster[a]	Total E-Cy3/QD added to solution[b]	Percentage bound
**Glk (dimer)**	3.9 ± 0.9	8.7	45 ± 10 %
**FBA (dimer)**	9.4 ± 2.1	13	72 ± 16 %
**PGK (monomer)**	5.0 ± 1.0	11.6	43 ± 8 %

[a]This methodology is unable to distinguish between labeled enzyme bound to individual QDs and to those bound to QD-clusters, but as we assume that the labeled enzyme will act similarly as the unlabeled enzyme there is no need to distinguish them. [b]These values are the 'Opt 2' proportion for the 7E mixture reported in the main text.

The same experimental format was undertaken with other enzymes including maltase (monomer), invertase (tetramer), and pyruvate kinase II (tetramer) as well as in assemblies that extended the number of enzymes to 7E, 8E, 9E, 11E, 12E and 13E cocktail systems with addition of the upstream saccharification enzymes and the downstream 4E system as shown in **Supplementary Table 12**.

Supplementary Table 12. Enzyme incorporation into QD-clusters in selected systems. The values for each are the FRET determined incorporation / the total amount of labeled enzyme added to the system (and finally the incorporation percentage).

Enzyme\Cocktail	7E	8E	9E	11E	12E	13E
Glk	3.9/8.7 (45%)	4.3/6.8 (63%)	3.6/6.0 (60%)	-	-	-
FBA	9.4/13.0 (72%)	2.9/10.6 (27%)	4.5/14.7 (30%)	2.9/7.5 (39%)	-	-
PGK	5.0/11.6 (43%)	4.0/9.9 (40%)	0.8/5.1 (16%)	-	-	-
Mal	-	-	3.0/6.5 (47%)	-	-	1.3/3.0 (44%)
Inv	-	2.6/11.9 (22%)	-	-	3.1/18.5 (17%)	-
PykA	-	-	-	4.8/10.7 (44%)	-	5.9/12.3 (47%)

Though not every combination was tested, overall the assay consistently reported values around 30-40 % incorporation with a minimum value of 16 % (for PGK in the 9E cocktail) and the highest observed value of 72 % (FBA in the 7E cocktail); these values are in line with the

excess enzyme to surface availability. We currently have no additional insight into whether the form of the enzyme (monomer/dimer/tetramer) modifies the incorporation rate of that enzyme into the cluster, nor if there is a preference for one enzyme over another. The latter points would be influenced by enzyme shape, location and availability of the $(His)_6$ on a given enzyme, number of actual binding sites on a NP material, how one enzyme can influence the attachment or incorporation of another, along with probably many other less discernable factor(s). We note that both the highest value and third lowest value were reported by the same enzyme (FBA in the 7E and 8E cluster respectively). Similarly, though the reported values are the result of at least 2 repeats, we caution any excessive interpretation into the specific values reported and suggest that they be taken as a general confirmation that all tested enzymes bind and that their conjugation is dependent on available QD surface area, making multilayer enzyme assembly less likely.

An assay of a similar nature was utilized to investigate what the percentage of bound enzyme was in the case of changing ratios of total enzyme to NP. We realized the experiment with labeled FBA and PGK enzymes though all enzymes are present in the 7E cocktail ratio. By decreasing the total amount of enzyme per QD, we observed a larger portion of the enzymes bound to the QDs, see **Supplementary Figure 42**. At the high enzyme to QD ratio (130-180 total E/QD), the bound percentage was 11 ± 1.6 % for FBA and 36 ± 5 % for PGK. For both enzymes, the percentage increases as the enzyme/QD ratio decreases. In fact, for the low ratio (~2E/QD), the percentage of binding is above 100% (250 ± 36 % for FBA and 331 ± 72 % for PGK). We interpret this as further support for the cluster formation of the QDs. At the lower ratio there is less than 1 acceptor per QD, but if the systems are forming clusters a single Cy3-labeled enzyme (with multiple dyes labeled on that protein) can act as an acceptor to multiple QD donors resulting in higher FRET. This is not to say that there is not cluster formation at the higher E/QD ratios as well.

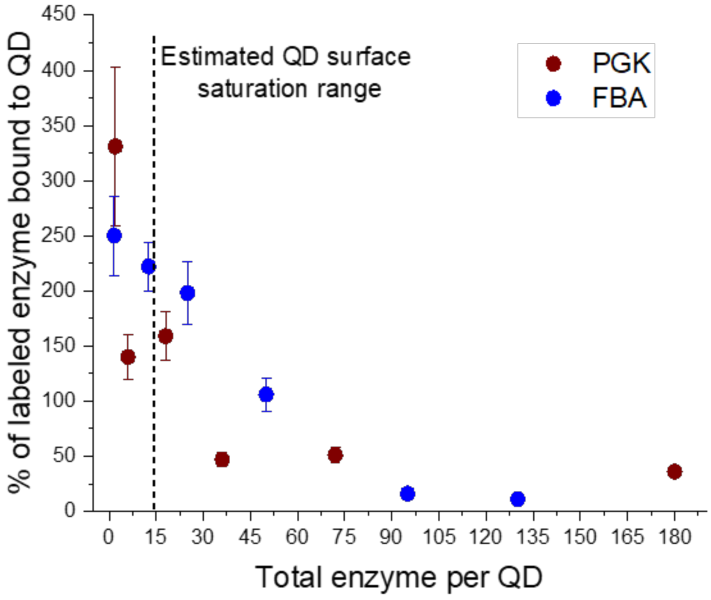

Supplementary Figure 43. Percentage of labeled enzyme bound to a QD surface as the total number of enzymes to QD increases. Data shown is the mean from n = 3 independent experimental samples ± standard deviation.

Numerical simulation of the formation of nanoparticle aggregates. The majority of the enzymes we utilize are multimeric (see Manuscript **Table 1**), and a salient fact about them in our work is that each subunit is terminated by its own poly histidine or $(His)_6$ tag. That these tags have a strong affinity for the QD/NPL particles[16, 30] implies that the multimeric enzymes have the potential to crosslink NPs, and in this way bring about the formation of NP clusters. Indeed we do observe such clustering experimentally, *e.g.*, see the TEM images of **Figure 4** in the main manuscript and also the TEM section and **Supplementary Figure 46** here in the examples in this section. To obtain further support for this conclusion, we performed numerical modeling of the enzyme-mediated cluster formation. As a basis for these simulations, we assume that the clustering rate is set by diffusion and the phenomenon is thus an example of classical diffusion-limited aggregation (DLA).[42, 43] Our goal is to formulate a DLA model and use it to better understand the system by comparing qualitative and semi-quantitative predictions with experiment (as opposed to using it to provide a pointless quantitative matching with experiment *via* curve-fitting).

Our DLA model assumes the system consists initially of free QDs and a mix of monomeric and/or dimeric enzymes. For purposes of computational efficiency, we restrict the model to two space dimensions and take it to be of the form of a cellular automaton in that we discretize space and time by situating the constituents at the nodes of a hexagonal lattice. Given this arrangement, the maximum velocity is one lattice spacing per time step, with slower speeds being achievable simply by introducing a probability of motion; in particular, we make the speeds inversely proportional to size in keeping with the Stokes-Einstein drag formula. Our general algorithm for computing the reaction-diffusion dynamics is presented in **Supplementary Figure 44**. At each time step, the constituents are sequentially evaluated for a potential move in a random direction, and if a collision occurs the move is either not made or results in an irreversible binding event of enzyme and QD/cluster. We make the probability of binding depend on the current coverage of the QD as a way of modeling the limited number of binding sites/access of a given QD.

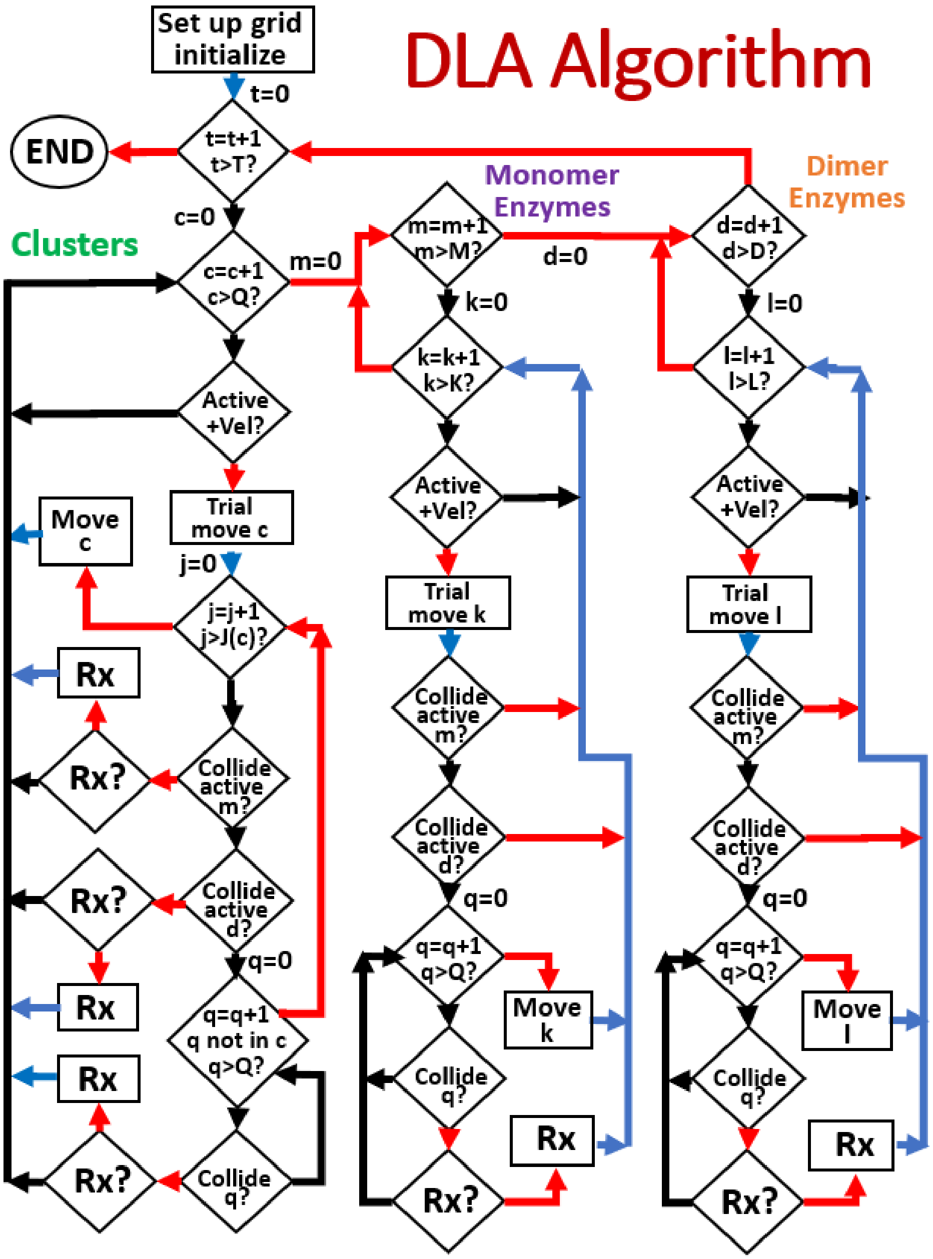

Supplementary Figure 44. General DLA algorithm where T is the total number of simulation time steps, M is the total number of different types of monomeric enzymes with K(m) being the number of type m, D is the total number of different types of dimeric enzymes with L(d) being the number of type d, Q is the number of QDs and J(c) is the total of QDs in cluster c. When enzymes and clusters can no longer react they are designated inactive and this is true also of clusters that disappear (*i.e.*, by uniting with another cluster). Initially, each QD is regarded as a "degenerate" cluster. Velocities can be reduced from the mesh velocity by introducing probabilities of movement that can depend on cluster size. Also reaction rates can be reduced as QDs become "passivated" by monomeric enzymes. Note that at all decision points in this flowchart, the red (black) arrow should be followed if the condition is true (false).

Before discussing simulation results it should be noted that if the initial system consisted of QDs and dimeric enzymes in excess, then after infinite time all of the QDs would be bound together into one giant cluster. However, as the QD clusters grow in size, they move ever more slowly, and therefore we expect an exponential slowing down of the growth in cluster size. In other words, one would expect the long-time behavior to be kinetically limited. By contrast, for the regime when the dimeric enzymes are not in excess ($[E_2]/[QD] < 1$), it would be more likely to see the steady state actually achieved. As a special case, if we assume that no "circular" clusters form, then it is readily shown that the average cluster size that will be reached in the steady state is:

$$\langle S\rangle_{ss} = \frac{1}{1 - [E_2]/[QD]} \qquad \text{for } [E_2] < [QD] \qquad \text{(Supplementary Eq. S5)}$$

This function is plotted in **Supplementary Figure 45** (red line). Again, when $[E_2]/[QD] \geq 1$, the steady state limit is a single very large cluster in which all of the QDs are joined, however, as this regime is approached (*i.e.*, in the vicinity of the $[E_2]/[QD] = 1$ asymptote) we expect a kinetically limited situation. Illustrating this behavior, in **Supplementary Figure 45** we show the average cluster sizes obtained after a long (but finite) time in numerical DLA simulations for various values of the $[E_2]/[QD]$ ratio.

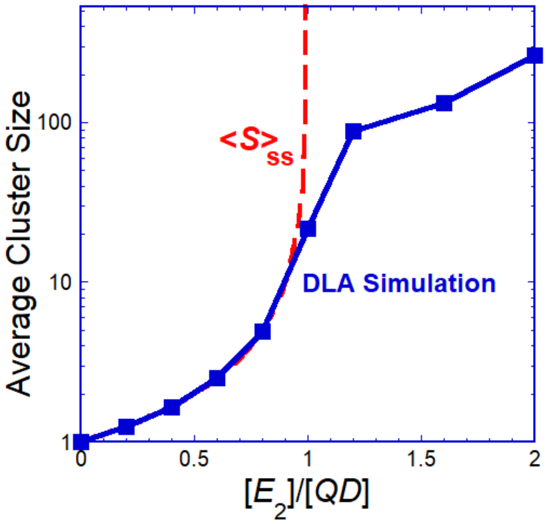

Supplementary Figure 45. For a system consisting of QDs and dimeric enzymes, a comparison of the analytical formula for steady state (S, red) and a DLA simulation (DLA, blue) that is kinetically limited.

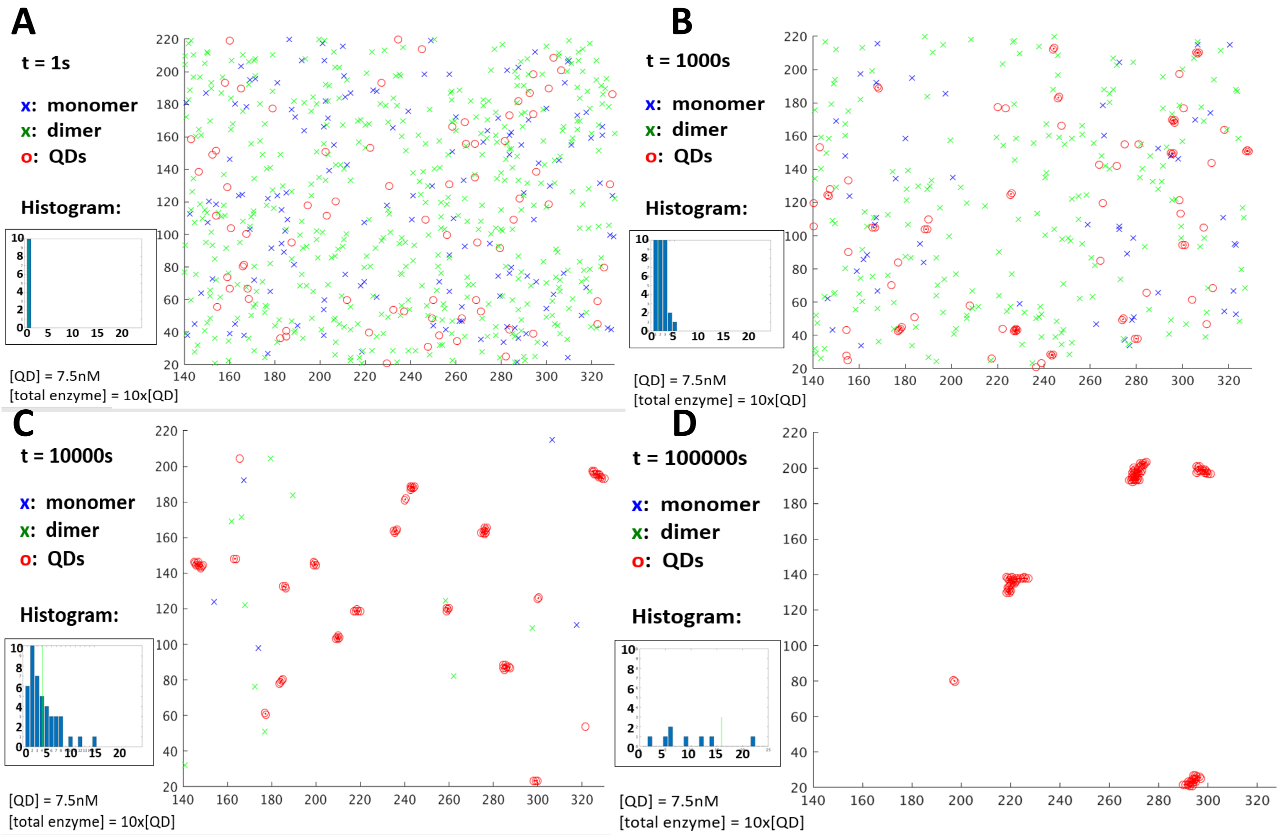

Supplementary Figure 46. Depictions of the DLA reaction of a system with QDs (red), monomeric enzymes (blue), and dimeric enzymes (green) after **(A)** 1s, **(B)** 1000s, **(C)** 10000s, and **(D)** 100000s. The inset gives a histogram of the cluster sizes with an average cluster size of 15 QDs after reacting for 100000s.

Turning next to cluster formation in a simulated reaction involving both monomeric and dimeric enzymes, in **Supplementary Figure 46** we show four close-up snapshots from a transient run after 1 sec, 1,000 sec, 10,000 sec, and 100,000 sec of simulated time. The "final" state in simulation (*i.e.*, after more than a day of simulated time) appears quite similar to what we observe experimentally in manuscript **Figure 4**, with a wide distribution of sizes and an average size of about 15 QDs. We take this, and other similar simulations (data not shown), to be further support for the idea that the clusters seen experimentally are generated by the multimeric enzymes driving a kinetically-limited DLA. Additionally, this view of the process of cluster formation implies that the clusters so formed will configure as small dendritic trees (as opposed, for example, to quasi-spherical agglomerations) that are characteristic of the DLA phenomenon and are seen in **Supplementary Figure 46D**.[42, 43]

Supplementary assay data

Comparison of enzyme activity when diluted on and off NP.

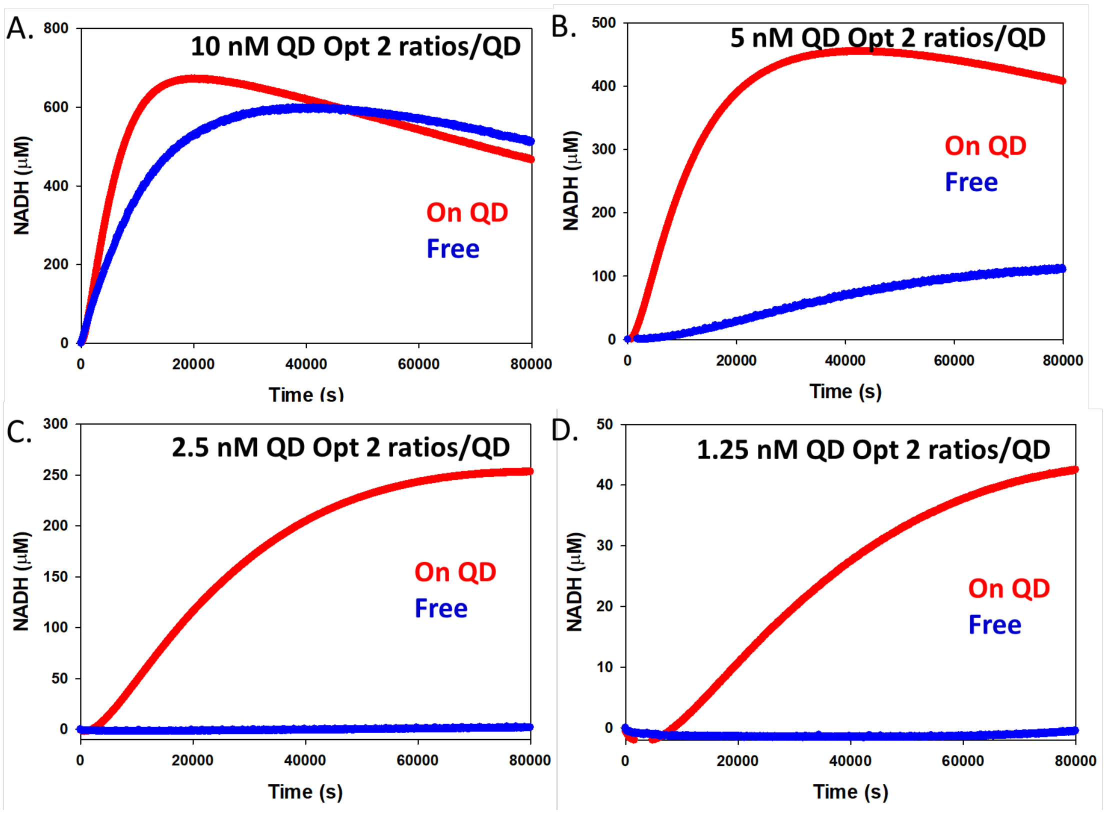

Supplementary Figure 47. Comparison of enzyme activity on/off QD with serial dilutions. 520 nm QDs were assembled with the 7 enzyme cascade that transforms glucose to 3-PG. Opt 2 ratios for each enzyme per QD were used during assembly; these are 5.5 Glk, 1 PGI, 9 FPK, 12 FBA, 1 TPI, 27 GPD, and 7.5 PGK per QD. Stock solutions of the QD-7E assemblies were then serially diluted for assay such that the QD concentration were **(A)** 10 nM, **(B)** 5 nM, **(C)** 2.5 nM, **(D)** 1.25 nM, and 0.63 nM. The concentration of enzyme present in each reaction is then obtained by multiplying the Opt 2 ratio of that enzyme by the QD concentration. For example, in panel A for the 10 nM QD sample, the enzyme concentrations present are 55 nM Glk, 10 nM PGI, 90 nM FPK, 120 nM FBA, 10 nM TPI, 270 nM GPD, and 75 nM PGK. Catalytic activity was then monitored by assaying NADH formation for each dilution *versus* the exact same amount of free enzyme without QD present as a control. No catalysis was observed for the 0.63 nM sample. As is seen in panel A, the total amount of enzyme present is too high for substantial channeling behavior to manifest as compared to the free enzyme control since this is most likely above diffusion limited conditions. Here the channeling manifests as increased overall rate during the initial time versus that of the free enzyme. However, by simply diluting the QD-enzyme cluster concentrations 2-fold channeling behavior is seen at 5 nM. Moreover, at 2.5 nM QD and lower concentrations, the free enzyme configuration does not result in any appreciable catalysis over the time scale measured. We ascribe the small drop in NADH concentration at later time points in panels A,B to GPD reverse reactions becoming more favorable as more 1,3-bisphosphoglycerate and 3-PG build up.

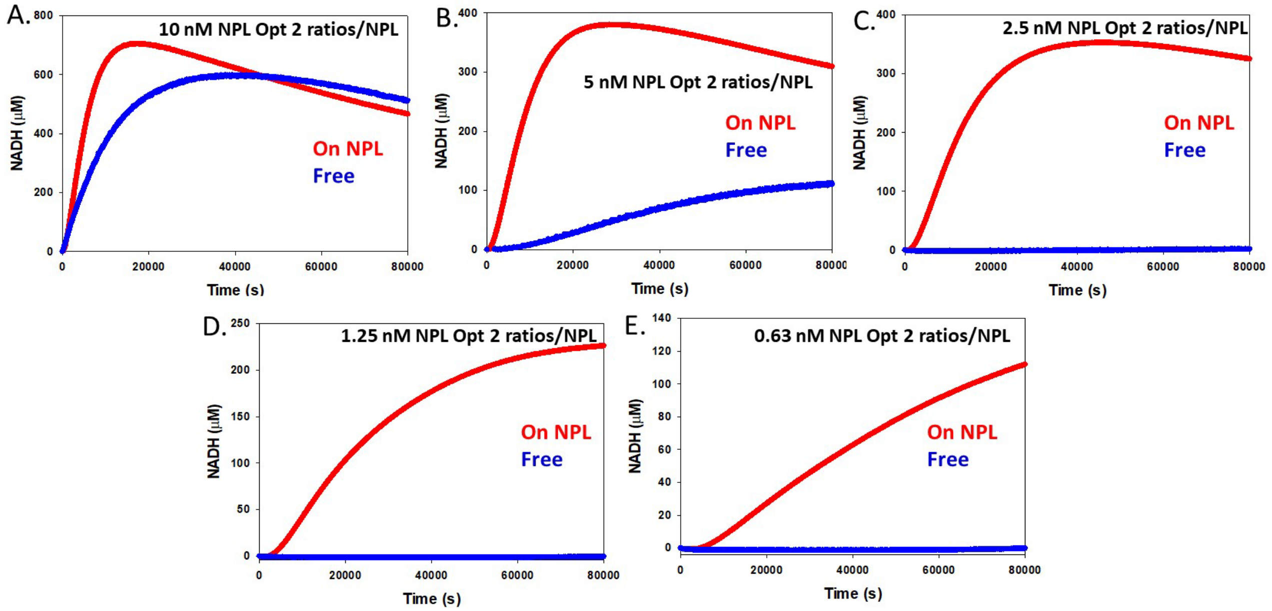

Supplementary Figure 48. Comparison of enzyme activity on/off NPL with serial dilutions. NPLs were assembled with the 7 enzyme cascade that transforms glucose to 3-PG. Opt 2 ratios for each enzyme per NPL were used during assembly; these are 5.5 Glk, 1 PGI, 9 FPK, 12 FBA, 1 TPI, 27 GPD, and 7.5 PGK per NPL. Stock solutions of the NPL-7E assemblies were then serially diluted for assay such that the NPL concentration were **(A)** 10 nM, **(B)** 5 nM, **(C)** 2.5 nM, **(D)** 1.25 nM, and **(E)** 0.63 nM. The concentration of enzyme present in each reaction is then obtained by multiplying Opt 2 ratio of that enzyme by the NPL concentration. For example, in panel A for the 10 nM NPL sample, the enzyme concentrations present are 55 nM Glk, 10 nM PGI, 90 nM FPK, 120 nM FBA, 10 nM TPI, 270 nM GPD, and 75 nM PGK. Catalytic activity was then monitored by assaying NADH formation for each dilution *versus* the exact same amount of free enzyme without NPL present as a control. As is seen in panel A, the total amount of enzyme present is too high for channeling behavior to manifest as compared to the free enzyme control since this would be above diffusion limitations. Here the channeling manifests as increased overall rate during the initial time versus that of the free enzyme. However, by simply diluting the NPL-enzyme cluster concentrations 2-fold channeling behavior is seen. Again, at 2.5 nM NPL and lower concentrations, the free enzyme configuration does not result in any appreciable catalysis over the time scale measured. In this configuration, significant catalysis was observed for the 0.63 nM sample (~16x dilution from 10 nM format) *versus* the previous QD format in **Supplementary Figure 47** attesting to the ability of the NPLs to assemble enzymes for these purposes. We ascribe the small drop in NADH concentration at later time points in panels A,B to GPD reverse reactions becoming more favorable as more 1,3-bisphosphoglycerate and 3-PG build up.

Enzyme kinetics at different ratios on NP. The following tables contain a summary listing of kinetic parameters determined for each individual enzyme both on/off QD or NPL as pertinent. For each assay, the total amount of enzyme was kept constant while the ratio of NP to enzyme was varied.

Supplementary Table 13. Enzyme and nanoparticle concentrations used for ratio *versus* activity assays.

Enzyme Name:	Enzyme Concentration (nM)	NP Final Concentration (nM)
**Amy**	2.5	0 - 10
**Mlt**	3	0 - 12
**Inv**	200	0 - 200
**Glk**	3	0 - 12
**PGI**	0.105	0 - 1.05
**PFK**	3	0 - 12
**FBA**	3	0 - 30
**TPI**	3	0 - 30
**GPD**	3	0 - 12
**PGK**	3	0 - 12
**PGM**	12	0 - 120
**Eno**	3	0 - 30
**PykA**	----	----
**LDH**	2.5	0-10

Supplementary Table 14. Amylase (Amy) with 520 QDs.

	Ratio of Enzyme / QD							
**Parameter**	**Free enzyme**	**0.25**	**0.5**	**1**	**2**	**4**	**8**	**16**
V_{max} **(µM s^{-1})**	0.063 ± 0.001	0.063 ± 0.003	0.059 ± 0.003	0.059 ± 0.003	0.071 ± 0.003	0.066 ± 0.002	0.057 ± 0.002	0.061 ± 0.002
K_M **(µM)**	213 ± 15	200 ± 37	198 ± 41	187 ± 39	257 ± 40	240 ± 31	186 ± 37	216 ± 33
k_{cat} **(s^{-1})**	25.0 ± 0.4	25.3 ± 1.0	23.6 ± 1.0	23.5 ± 1.0	28.2 ± 1.0	26.6 ± 0.8	22.7 ± 1.0	24.4 ± 0.8
k_{cat}/K_M **(mM^{-1}s^{-1})**	118 ± 8	127 ± 24	119 ± 25	126 ± 27	110 ± 18	111 ± 15	122 ± 25	113 ± 18
**S.A. (µmol min^{-1} mg^{-1})**	25.2 ± 0.4	25.4 ± 1.0	23.7 ± 1.0	23.6 ± 1.0	28.4 ± 1.0	26.7 ± 0.8	22.8 ± 1.0	24.5 ± 0.8

Notes: Assayed using coupled enzymatic assay. **Notes:** Kinetic values based on monomer MW.

Supplementary Table 15. Maltase (Mal) with 520 QDs.

	Ratio of Enzyme / QD							
**Parameter**	**Free enzyme**	**0.25**	**0.5**	**1**	**2**	**4**	**8**	**16**
V_{max} (μM s^{-1})	0.001 ± 0.001	0.063 ± 0.003	0.044 ± 0.001	0.060 ± 0.002	0.046 ± 0.002	0.028 ± 0.008	0.018 ± 0.001	0.060 ± 0.002
K_M (μM)	335 ± 80	496 ± 70	249 ± 32	401 ± 49	341 ± 58	247 ± 29	305 ± 40	390 ± 39
k_{cat} (s^{-1})	0.40 ± 0.03	21.3 ± 1.0	14.7 ± 0.5	20.1 ± 0.7	15.3 ± 0.7	9.3 ± 0.3	6.2 ± 0.2	20.1 ± 0.6
k_{cat}/K_M ($mM^{-1}s^{-1}$)	1.2 ± 0.30	42.8 ± 6.3	59.0 ± 7.8	50.2 ± 6.4	44.9 ± 7.9	37.6 ± 4.5	20.3 ± 2.7	51.6 ± 5.4
S.A. (μmol min^{-1} mg^{-1})	0.32 ± 0.02	16.9 ± 0.7	11.7 ± 0.4	16.1 ± 0.6	12.2 ± 0.6	7.4 ± 0.2	4.9 ± 0.2	16.1 ± 0.5

Notes: Assayed using direct enzymatic assay with commercial 4-nitrophenyl-α-D-glucopyranoside (4NDG) substrate. Maltase = 3 nM
Kinetic values based on monomer MW.

Supplementary Table 16. Maltase with NPLs.

	Ratio of Enzyme / NPL							
	**Free enzyme**	**0.25**	**0.5**	**1**	**2**	**4**	**8**	**16**
V_{max} (μM s^{-1})	0.001 ± 0.001	0.355 ± 0.021	0.245 ± 0.034	0.328 ± 0.018	0.088 ± 0.020	0.136 ± 0.020	0.091 ± 0.006	0.017 ± 0.001
K_M (μM)	335 ± 80	1751 ± 229	1742 ± 516	1966 ± 219	275 ± 221	903 ± 359	1241 ± 211	113 ± 40
k_{cat} (s^{-1})	0.40 ± 0.03	59.1 ± 3.6	40.8 ± 5.6	54.7 ± 2.9	14.6 ± 3.3	22.7 ± 3.4	15.2 ± 1.1	2.8 ± 0.2
k_{cat}/K_M ($mM^{-1}s^{-1}$)	1.2 ± 0.30	33.76 ± 4.86	23.42 ± 7.64	27.81 ± 3.44	53.02 ± 44.12	25.17 ± 10.68	12.27 ± 2.26	24.81 ± 8.93
S.A. (μmol min^{-1} mg^{-1})	0.32 ± 0.02	47.6 ± 2.9	32.9 ± 4.5	44 ± 2.4	11.8 ± 2.6	18.3 ± 2.7	12.3 ± 0.9	2.3 ± 0.2

Notes: Assayed using direct enzymatic assay with commercial 4-nitrophenyl-α-D-glucopyranoside (4NDG) substrate. Maltase = 6 nM
Kinetic values based on monomer MW.

Supplementary Table 17. Invertase (Inv) with 520 QDs.

Ratio of Enzyme / QD				
**Parameter**	**Free enzyme**	**1**	**2**	**4**
V_{max} (μM s^{-1})	0.014 ± 0.001	0.019 ± 0.001	0.022 ± 0.002	0.104 ± 0.011
K_M (μM)	191 ± 100	1388 ± 597	3620 ± 1310	---
k_{cat} (s^{-1})	0.07 ± 0.00	0.10 ± 0.01	0.12 ± 0.01	0.55 ± 0.06
k_{cat}/K_M (mM^{-1}s^{-1})	0.39 ± 0.21	0.07 ± 0.03	0.03 ± 0.01	---
S. A. (μmol min^{-1} mg^{-1})	0.024 ± 0.001	0.032 ± 0.002	0.038 ± 0.003	0.18 ± 0.02

Notes: Assayed using a coupled assay format. '---'designates that useful values could not be determined.
No useful values could be determined for any other ratios due to this enzymes slow catalysis.

Supplementary Table 18. Invertase with NPLs.

Ratio of Enzyme / NPL			
**Parameter**	**Free enzyme**	**1**	**2**
V_{max} (nM s^{-1})	0.014 ± 0.001	0.007 ± 0.00	0.005 ± 0.00
K_M (μM)	191 ± 100	---	264 ± 341
k_{cat} (s^{-1})	0.07 ± 0.00	0.04 ± 0.01	0.03 ± 0.00
k_{cat}/K_M (mM^{-1}s^{-1})	0.39 ± 0.21	---	0.10 ± 0.13
S. A. (μmol min^{-1} mg^{-1})	0.024 ± 0.001	0.012 ± 0.001	0.008 ± 0.001

Notes: Assayed using a coupled assay format. '---'designates that useful values could not be determined.
No useful values could be determined for any other ratios due to this enzyme's extremely slow catalysis.

Supplementary Table 19. Glucokinase (Glk) with 520 QDs.

Ratio of Enzyme / QD						
**Parameter**	**Free enzyme**	**0.25**	**0.5**	**1**	**2**	**4**
V_{max} (μM s^{-1})	0.013 ± 0.001	0.147 ± 0.003	0.180 ± 0.005	0.176 ± 0.005	0.163 ± 0.021	0.102 ± 0.003
K_M (μM)	36 ± 10	116 ± 13	110 ± 13	127 ± 18	100 ± 57	90 ± 11
k_{cat} (s^{-1})	2.6 ± 0.1	29.3 ± 0.7	35.8 ± 0.9	35.3 ± 1.1	32.6 ± 4.2	20.4 ± 0.5
k_{cat}/K_M ($mM^{-1}s^{-1}$)	73 ± 21	252 ± 28	325 ± 40	278 ± 40	325 ± 191	226 ± 27
S.A. (μmol min^{-1} mg^{-1})	4.2 ± 0.2	47.7 ± 1.1	58.2 ± 1.5	57.4 ± 1.7	53.0 ± 6.8	33.1 ± 0.6

Notes: Assayed using coupled enzymatic assay. Kinetic values based on monomer MW.

Supplementary Table 20. Glucokinase (Glk) with NPLs.

Ratio of Enzyme / NPL						
**Parameter**	**Free enzyme**	**0.25**	**0.5**	**1**	**2**	**4**
V_{max} (μM s^{-1})	0.013 ± 0.001	0.043 ± 0.001	0.038 ± 0.001	0.028 ± 0.001	0.010 ± 0.001	0.011 ± 0.001
K_M (μM)	36 ± 10	70 ± 7	47 ± 6	41 ± 5	---	---
k_{cat} (s^{-1})	2.6 ± 0.1	14.5 ± 0.2	12.5 ± 0.2	9.4 ± 0.2	3.5 ± 0.1	3.6 ± 0.1
k_{cat}/K_M ($mM^{-1}s^{-1}$)	73 ± 21	206 ± 21	266 ± 36	230 ± 28	---	---
S.A. (μmol min^{-1} mg^{-1})	4.2 ± 0.2	11.8 ± 0.2	10.2 ± 0.2	7.7 ± 0.1	2.9 ± 0.1	2.9 ± 0.1

Notes: '---'designates that useful values could not be determined.

Supplementary Table 21. Phosphoglucose isomerase (PGI) with 520 QDs.

	Ratio of Enzyme / QD							
**Parameter**	**Free enzyme**	**0.1**	**0.25**	**0.5**	**1**	**2**	**4**	**8**
V_{max} (μM s^{-1})	0.261 ± 0.006	0.227± 0.005	0.213 ± 0.002	0.217 ± 0.004	0.203 ± 0.009	0.208 ± 0.004	0.204 ± 0.003	0.20 ± 0.003
K_M (μM)	393 ± 31	442 ± 32	391 ± 16	429 ± 27	324 ± 54	406 ± 26	367 ± 22	357 ± 20
k_{cat} (s^{-1})	2481 ± 53	2165 ± 44	2025 ± 23	2066 ± 36	1929 ± 85	1982 ± 35	1941 ± 31	1906 ± 29
k_{cat}/K_M (mM^{-1}s^{-1})	6313 ± 518	4900 ± 368	5178 ± 221	4818 ± 315	5954 ± 1030	4882 ± 329	5294 ± 323	5335 ± 315
S.A. (μmol min^{-1} mg^{-1})	2338 ± 50	2040 ± 41	1908 ± 22	1947 ± 34	1817 ± 80	1868 ± 33	1829 ± 29	1796 ± 27

Notes: Assayed using commercial enzyme assay kit**.** Kinetic values based on monomer MW

Supplementary Table 22. Phosphofructokinase I isomerase (PFK) with 520 QDs.

	Ratio of Enzyme / QD							
**Parameter**	**Free enzyme**	**0.1**	**0.2**	**0.5**	**1**	**2**	**4**	**8**
V_{max} (μM s^{-1})	0.030 ± 0.001	0.030 ± 0.001	0.032 ± 0.001	0.031 ± 0.001	0.029 ± 0.001	0.026 ± 0.001	0.023 ± 0.001	0.019 ± 0.001
K_M (μM)	202 ± 21	239 ± 32	210 ± 24	198 ± 24	198 ± 24	169 ± 20	170 ± 24	116 ± 18
k_{cat} (s^{-1})	10.1 ± 0.2	9.9 ± 0.3	10.6 ± 0.3	10.2 ± 0.3	9.5 ± 0.2	8.7 ± 0.2	7.7 ± 0.2	6.5 ± 0.2
k_{cat}/K_M (mM^{-1}s^{-1})	50.2 ± 5.3	41.3 ± 5.6	50.7 ± 6.0	51.5 ± 6.4	48.2 ± 6.0	51.5 ± 6.2	45.2 ± 6.5	55.7 ± 8.6
S.A. (μmol min^{-1} mg^{-1})	17.6 ± 0.4	17.1 ± 0.5	18.4 ± 0.5	17.7 ± 0.5	16.5 ± 0.4	15.1 ± 0.3	13.4 ± 0.4	11.2 ± 0.3

Notes: Assayed using commercial enzyme assay kit. Kinetic values based on monomer MW

Supplementary Table 23. Phosphofructokinase I isomerase (PFK) with NPLs.

	Ratio of Enzyme / NPL							
**Parameter**	**Free enzyme**	**0.25**	**0.5**	**1**	**2**	**4**	**8**	**12**
V_{max} (nM s^{-1})	2.25 ± 0.08	3.91 ± 0.11	3.54 ± 0.14	3.57 ± 0.10	3.03 ± 0.12	2.73 ± 0.14	2.39 ± 0.12	3.35 ± 0.32
K_M (μM)	3.43 ± 8.62	50.03 ± 10.94	43.96 ± 16.08	72.72 ± 13.95	103.30 ± 25.62	215.40 ± 61.89	86.02 ± 27.06	200.50 ± 102.30
k_{cat} (s^{-1})	0.75 ± 0.03	1.30 ± 0.04	1.18 ± 0.05	1.19 ± 0.03	1.01 ± 0.04	0.91 ± 0.05	0.80 ± 0.04	1.12 ± 0.11
k_{cat}/K_M (mM^{-1}s^{-1})	218.2 ± 547.9	26.1 ± 5.7	26.8 ± 9.9	16.4 ± 3.2	9.8 ± 2.5	4.2 ± 1.2	9.3 ± 2.9	5.6 ± 2.9
S.A. (μmol min^{-1} mg^{-1})	0.32 ± 0.01	0.57 ± 0.02	0.51 ± 0.02	0.52 ± 0.01	0.44 ± 0.02	0.39 ± 0.02	0.35 ± 0.02	0.48 ± 0.05

Notes: Assayed using commercial enzyme assay kit. Kinetic values based on monomer MW.

Supplementary Table 24. Fructose-bisphosphate aldolase (FBA) with 520 QDs.

	Ratio of Enzyme / QD							
**Parameter**	**Free enzyme**	**0.1**	**0.2**	**0.5**	**1**	**2**	**4**	**8**
V_{max} (μM s^{-1})	0.020 ± 0.001	0.017 ± 0.001	0.019 ± 0.001	0.017 ± 0.001	0.017 ± 0.001	0.017 ± 0.001	0.016 ± 0.001	0.016 ± 0.001
K_M (μM)	216 ± 23	177 ± 28	156 ± 15	127 ± 26	191 ± 23	164 ± 21	149 ± 24	155 ± 28
k_{cat} (s^{-1})	6.8 ± 0.2	5.7 ± 0.2	6.2 ± 0.1	5.5 ± 0.2	5.9 ± 0.2	5.5 ± 0.2	5.4 ± 0.2	5.5 ± 0.2
k_{cat}/K_M ($mM^{-1}s^{-1}$)	31.4 ± 7.3	32.3 ± 7.2	38.8 ± 8.8	43.4 ± 9.0	30.7 ± 7.3	33.9 ± 7.9	36.4 ± 8.2	35.2 ± 8.2
S.A. (μmol min^{-1} mg^{-1})	9.8 ± 0.2	8.3 ± 0.3	9.0 ± 0.2	8.0 ± 0.3	8.5 ± 0.2	8.0 ± 0.2	7.9 ± 0.3	7.9 ± 0.3

Notes: Assayed using commercial enzyme assay kit. Kinetic values based on monomer MW.

Supplementary Table 25. Triose phosphate isomerase (TPI) with 520 QDs.

	Ratio of Enzyme / QD							
**Parameter**	**Free enzyme**	**0.1**	**0.25**	**0.5**	**1**	**2**	**4**	**8**
V_{max} (μM s^{-1})	0.582 ± 0.035	0.745 ± 0.049	0.717 ± 0.035	0.582 ± 0.037	0.533 ± 0.014	0.806 ± 0.048	0.619 ± 0.029	0.524 ± 0.013
K_M (μM)	1717 ± 236	3144 ± 395	2984 ± 277	2045 ± 280	1728 ± 101	3241 ± 360	2340 ± 232	1802 ± 100
k_{cat} (s^{-1})	194 ± 12	249 ± 17	239 ± 12	194 ± 12	178 ± 5	269 ± 16	207 ± 10	175 ± 4
k_{cat}/K_M ($mM^{-1}s^{-1}$)	113 ± 17	79 ± 11	80 ± 8	95 ± 14	103 ± 7	83 ± 10	88 ± 10	97 ± 6
S.A. (μmol min^{-1} mg^{-1})	401 ± 24	513 ± 34	493 ± 24	401 ± 25	367 ± 10	555 ± 33	426 ± 20	360 ± 9

Notes: Assayed using coupled enzymatic assay progress curve. Kinetic values based on monomer MW.

Supplementary Table 26. Glyceraldehyde-3-phosphate dehydrogenase (GPD) with 520 QDs.

	Ratio of Enzyme / QD							
**Parameter**	**Free enzyme**	**0.25**	**0.5**	**1**	**2**	**4**	**8**	**12**
V_{max} (μM s^{-1})	0.010 ± 0.001	0.050 ± 0.004	0.037 ± 0.002	0.022 ± 0.001	0.019 ± 0.001	0.015 ± 0.001	0.011 ± 0.001	0.010 ± 0.001
K_M (μM)	3494 ± 1039	2872 ± 727	2495 ± 592	1344 ± 273	1321 ± 311	917 ± 250	238 ± 85	169 ± 55
k_{cat} (s^{-1})	3.2 ± 0.04	16. 7 ± 1.2	12.3 ± 0.8	7.38 ± 0.3487	6.5 ± 0.4	5.1 ± 0.3	3.6 ± 0.2	3.3 ± 0.1
k_{cat}/K_M (mM^{-1}s^{-1})	0.92 ± 0.01	5.80 ± 1.52	4.90 ± 1.20	5.49 ± 1.15	4.90 ± 1.18	5.56 ± 1.55	15.15 ± 5.49	19.48 ± 6.36
**S.A. (μmol min^{-1} mg^{-1})**	1.2 ± 0.1	6.6 ± 0.5	4.9 ± 0.3	2.9 ± 0.1	2.6 ± 0.1	2.0 ± 0.1	1.4 ± 0.1	1.3 ± 0.1

Notes: Assayed using direct NADH monitoring. Kinetic values based on monomer MW.

Supplementary Table 27. Glyceraldehyde-3-phosphate dehydrogenase (GPD) with NPLs.

	Ratio of Enzyme / NPL							
**Parameter**	**Free enzyme**	**0.25**	**0.5**	**1**	**2**	**4**	**8**	**12**
V_{max} (μM s^{-1})	0.010 ± 0.001	0.031 ± 0.001	0.024 ± 0.001	0.022 ± 0.001	0.037 ± 0.002	0.042 ± 0.003	0.027 ± 0.002	0.021 ± 0.001
K_M (μM)	3494 ± 1039	696 ± 153	1296 ± 267	1726 ± 399	4567 ± 882	5675 ± 1047	2892 ± 600	1300 ± 325
k_{cat} (s^{-1})	3.2 ± 0.04	10.3 ± 0.5	7.9 ± 0.4	7.3 ± 0.4	12.3 ± 0.7	14.0 ± 0.8	9.0 ± 0.5	6.9 ± 0.4
k_{cat}/K_M (mM^{-1}s^{-1})	0.92 ± 0.01	14.85 ± 3.32	6.14 ± 1.29	4.25 ± 1.01	2.69 ± 0.54	2.47 ± 0.48	3.13 ± 0.67	5.31 ± 1.37
**S.A. (μmol min^{-1} mg^{-1})**	1.2 ± 0.1	4.1 ± 0.2	3.2 ± 0.2	2.9 ± 0.2	4.9 ± 0.3	5.6 ± 0.3	3.6 ± 0.2	2.7 ± 0.2

Notes: Assayed using direct NADH monitoring. Kinetic values based on monomer MW.

Supplementary Table 28. Phosphoglycerate kinase (PGK) with 520 QDs.

	Ratio of Enzyme / QD							
**Parameter**	**Free enzyme**	**0.1**	**0.25**	**0.5**	**1**	**2**	**4**	**8**
V_{max} (μM s^{-1})	0.054 ± 0.003	0.169 ± 0.010	0.160 ± 0.007	0.142 ± 0.005	0.100 ± 0.003	0.067 ± 0.003	0.073 ± 0.002	0.061 ± 0.002
K_M (μM)	1048 ± 171	867 ± 164	1009 ± 152	942 ± 107	1052 ± 109	820 ± 130	1139 ± 106	862 ± 116
k_{cat} (s^{-1})	18 ± 1	57 ± 3	54 ± 2	48 ± 2	34 ± 1	23 ± 1	25 ± 1	21 ± 1
k_{cat}/K_M ($mM^{-1}s^{-1}$)	17.3 ± 3.0	65.8 ± 13.0	53.7 ± 8.4	51.1 ± 6.0	32.3 ± 3.5	27.6 ± 4.5	21.7 ± 2.1	23.8 ± 3.3
S.A. (μmol min^{-1} mg^{-1})	25.1 ± 1.3	79.1 ± 4.5	75.1 ± 3.3	66.7 ± 2.3	47.0 ± 1.5	31.3 ± 1.4	34.3 ± 1.0	28.4 ± 1.1

Notes: Assayed using coupled enzymatic assay. Kinetic values based on monomer MW.

Supplementary Table 29. Phosphoglycerate kinase (PGK) with NPLs.

	Ratio of Enzyme / NPLs							
**Parameter**	**Free enzyme**	**0.25**	**0.5**	**1**	**2**	**4**	**8**	**12**
V_{max} (μM s^{-1})	0.056 ± 0.002	0.132 ± 0.009	0.135 ± 0.007	0.120 ± 0.005	0.092 ± 0.005	0.069 ± 0.004	0.072 ± 0.005	0.089 ± 0.008
K_M (μM)	1267 ± 143	3414 ± 533	2782 ± 353	2148 ± 250	2077 ± 321	1439 ± 241	1900 ± 360	2684 ± 555
k_{cat} (s^{-1})	19 ± 01	44 ± 3	45 ± 2	40 ± 2	31 ± 2	23 ± 1	24 ± 2	30 ± 3
k_{cat}/K_M ($mM^{-1}s^{-1}$)	14.7 ± 1.7	12.9 ± 2.2	16.2 ± 2.2	18.6 ± 2.3	14.8 ± 2.4	15.9 ± 2.8	12.7 ± 2.5	11.1 ± 2.5
S.A. (μmol min^{-1} mg^{-1})	25.8 ± 0.9	60.8 ± 4.1	62.3 ± 3.2	55.3 ± 2.4	42.6 ± 2.4	31.7 ± 1.78	33.4± 2.3	41.2 ± 3.6

Notes: Assayed using commercial enzyme assay kit. Kinetic values based on monomer MW.

Supplementary Table 30. Phosphoglycerate mutase (PGM) with 520 QDs.

	Ratio of Enzyme / QD							
**Parameter**	**Free enzyme**	**0.1**	**0.25**	**0.5**	**1**	**2**	**4**	**8**
V_{max} (μM s^{-1})	0.012 ± 0.001	0.068 ± 0.004	0.064 ± 0.004	0.057 ± 0.004	0.055 ± 0.004	0.069 ± 0.004	0.058 ± 0.003	0.043 ± 0.002
K_M (μM)	3584 ± 1138	3995 ± 894	3381 ± 865	3331 ± 889	2558 ± 81	3454 ± 749	2949 ± 677	2725 ± 595
k_{cat} (s^{-1})	1.0 ± 0.1	5.7 ± 0.3	5.4 ± 0.3	4.8 ± 0.3	4.6 ± 0.3	5.8 ± 0.3	4.8 ± 0.3	3.6 ± 0.2
k_{cat}/K_M ($mM^{-1}s^{-1}$)	0.29 ± 0.09	1.430± 0.33	1.59 ± 0.42	1.43 ± 0.39	1.80 ± 0.58	1.67 ± 0.37	1.64 ± 0.39	1.33 ± 0.30
S.A. (μmol min^{-1} mg^{-1})	1.05 ± 0.09	5.88 ± 0.34	5.54 ± 0.35	4.90 ± 0.34	4.73 ± 0.35	5.93 ± 0.33	4.99 ± 0.28	3.73 ± 0.20

Notes: Assayed using coupled enzymatic assay. Kinetic values based on monomer MW.

Supplementary Table 31. Phosphoglycerate mutase with NPLs.

	Ratio of Enzyme / NPL							
**Parameter**	**Free enzyme**	**0.25**	**0.5**	**1**	**2**	**4**	**8**	**12**
V_{max} (μM s^{-1})	0.012 ± 0.001	0.025 ± 0.00	0.033 ± 0.002	0.034 ± 0.002	0.034 ± 0.002	0.027 ± 0.002	0.029 ± 0.002	0.023 ± 0.001
K_M (μM)	4624 ± 1710	3684 ± 1793	2886 ± 936	4244 ± 1020	3763 ± 995	3236 ± 873	4281 ± 1055	3733 ± 892
k_{cat} (s^{-1})	0.7 ± 0.0	1.5 ± 0.0	2.0 ± 0.0	2.1 ± 0.0	2.1 ± 0.0	1.7 ± 0.0	1.8 ± 0.0	1.4 ± 0.0
k_{cat}/K_M ($mM^{-1}s^{-1}$)	0.16 ± 0.06	0.42 ± 0.2	0.71 ± 0.23	0.49 ± 0.12	0.56 ± 0.15	0.52 ± 0.14	0.43 ± 0.11	0.39 ± 0.09
S.A. (μmol min^{-1} mg^{-1})	0.74 ± 0.07	1.58 ± 0.19	2.10 ± 0.16	2.15 ± 0.13	2.18 ± 0.14	1.74 ± 0.11	1.88 ± 0.12	1.49 ± 0.09

Notes: Assayed using coupled enzymatic assay. Kinetic values based on monomer MW.

Supplementary Table 32. Enolase (Eno) with 520 QDs.

	Ratio of Enzyme / QD							
**Parameter**	**Free enzyme**	**0.1**	**0.2**	**0.5**	**1**	**2**	**4**	**8**
V_{max} (μM s^{-1})	0.069 ± 0.009	0.110 ± 0.014	0.107 ± 0.014	0.079 ± 0.010	0.080 ± 0.009	0.063 ± 0.008	0.079 ± 0.009	0.039 ± 0.004
K_M (μM)	2040 ± 72	2609 ± 847	2506 ± 843	2279 ± 727	2050 ± 633	2097 ± 710	2782 ± 765	2075 ± 575
k_{cat} (s^{-1})	23 ± 3	37 ± 5	35 ± 5	26 ± 3	26 ± 3	21 ± 3	26 ± 3	13 ± 1
k_{cat}/K_M (mM^{-1}s^{-1})	11.2 ± 4.3	14.0 ± 4.9	14.1 ± 5.1	11.4 ± 3.9	12.9 ± 4.2	10.0 ± 3.6	9.3 ± 2.8	6.2 ± 1.8
S.A. (μmol min^{-1} mg^{-1})	28.7 ± 3.7	45.9 ± 5.8	44.3 ± 5.8	32.7 ± 3.9	33.2 ± 3.8	26.2 ± 3.3	32.5 ± 3.6	16.1 ± 1.7

Notes: Assayed using coupled enzymatic assay. Kinetic values based on monomer MW.

Note: Pyruvate kinase II (PykA) values used are from ref.[17]

Supplementary Table 33. Lactate Dehydrogenase (LDH) with 520 QDs.

	Ratio of Enzyme / QD							
**Parameter**	**Free enzyme**	**0.25**	**0.5**	**1**	**2**	**4**	**8**	**12**
V_{max} (μM s^{-1})	0.24 ± 0.03	0.52 ± 0.03	0.51 ± 0.02	0.54 ± 0.02	0.48 ± 0.02	0.55 ± 0.07	0.48 ± 0.04	0.72 ± 0.21
K_M (μM)	31470 ± 6901	7148 ± 1071	10640 ± 1360	14790 ± 1571	14350 ± 1734	33590 ± 8705	34310 ± 6011	31040 ± 7550
k_{cat} (s^{-1})	95.5 ± 10.4	209.3 ± 10.1	203.2 ± 9.3	216.6 ± 9.1	191.9 ± 8.7	221 ± 29.5	192.4 ± 17.5	289.3 ± 83.1
k_{cat}/K_M ($mM^{-1}s^{-1}$)	3.04 ± 0.74	29.28 ± 4.61	19.1 ± 2.59	14.64 ± 1.67	13.37 ± 1.72	6.58 ± 1.92	5.61 ± 1.11	3.18 ± 1.6
S.A. (μmol min^{-1} mg^{-1})	37 ± 4	81.1 ± 3.9	78.8 ± 3.6	83.9 ± 3.5	74.4 ± 3.4	85.7 ± 11.4	74.6 ± 6.8	112.1 ± 32.2

Notes: Assayed using direct NADH monitoring. Kinetic values based on monomer MW.

Supplementary Table 34. Lactate Dehydrogenase (LDH) with NPLs.

	Ratio of Enzyme / NPL							
**Parameter**	**Free enzyme**	**0.25**	**0.5**	**1**	**2**	**4**	**8**	**12**
V_{max} (μM s^{-1})	0.24 ± 0.03	0.66 ± 0.03	0.58 ± 0.02	0.43 ± 0.02	0.43 ± 0.02	0.2 ± 0.01	0.17 ± 0.01	0.15 ± 0.01
K_M (μM)	31470 ± 6901	8143 ± 1230	10380 ± 996	16620 ± 1955	27680 ± 3082	16940 ± 1647	12480 ± 1564	14070 ± 1506
k_{cat} (s^{-1})	95.5 ± 10.4	264.4 ± 13.4	231 ± 8	171.6 ± 8.3	172.2 ± 9.3	79.3 ± 3.2	67.8 ± 3.2	61.5 ± 2.6
k_{cat}/K_M ($mM^{-1}s^{-1}$)	3.04 ± 0.74	32.47 ± 5.17	22.26 ± 2.27	10.33 ± 1.31	6.22 ± 0.77	4.68 ± 0.49	5.43 ± 0.73	4.37 ± 0.5
S.A. (μmol min^{-1} mg^{-1})	37 ± 4	102.5 ± 5.2	89.5 ± 3.1	66.5 ± 3.2	66.7 ± 3.6	30.8 ± 1.2	26.3 ± 1.2	23.8 ± 1

Notes: Assayed using direct NADH monitoring. Kinetic values based on monomer MW.

Supplementary Table 35. Mass spectral analysis of QD and enzyme activity on and off/QD.

Enzymatic Reaction		Target analyte	MS SIR (m/z)		Analytical Chromatogram for Substate+		
Substrate	E[2]		Predicted	Observed	+E[2]	+E@QD[3]	+QD[4]
Maltoheptaose	Amylase	Maltose	341.11	341.03	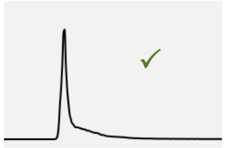	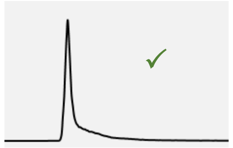	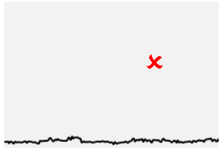
Maltose*	Maltase	Glucose	179.06	178.97	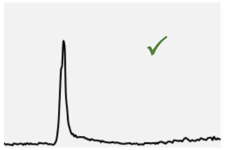	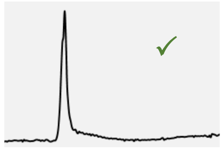	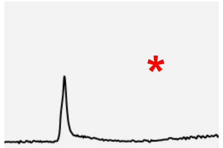
Sucrose	Invertase	Glucose (Fructose also detected)	179.06	179.05	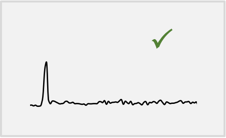	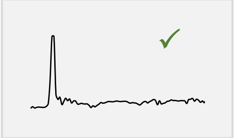	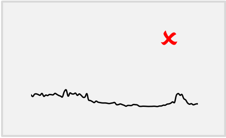

Glucose	Glk[5]	Glucose-6-Phosphate	259.13	258.97	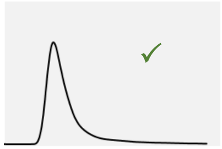	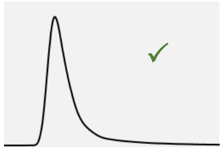	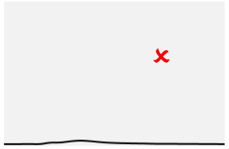
Fructose-6-Phosphate	PFK[6]	Fructose-1,6-Biphosphate	339.11	338.97	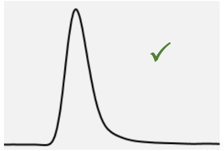	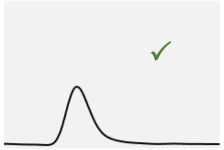	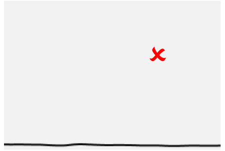
Fructose-1,6-Biphosphate	FBA[7]	Glyceraldehyde-3-Phosphate	169.05	168.90	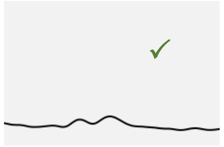	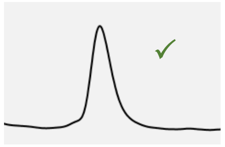	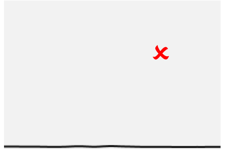
3-Phosphoglycerate	PGM[8] + ENO[9]	Phosphoenolpyruvate	167.03	166.90	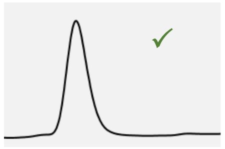	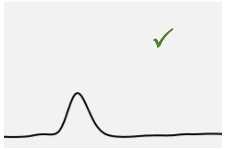	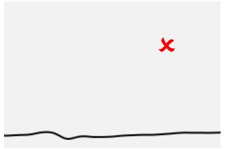

✓ ✓

Phosphoenolpyruvate	PykA[10]	Pyruvate	87.05	87.00	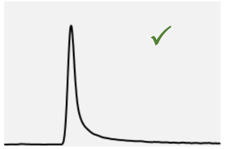	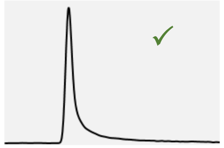	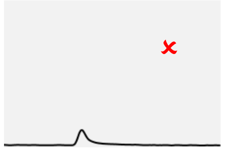
Pyruvate	LDH[11]	Lactate	89.07	89.00	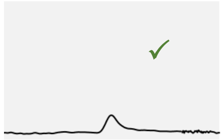	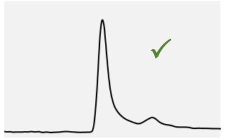	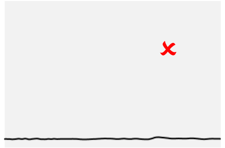

Notes:[1]SIR: Single Ion Recording; [2]E: Enzyme; [3]E@QD:Enzyme on Quantum Dots; [4]QD: Quantum Dots (see text for details); [5]Glk: Glucokinase; [6]PFK: Phosphofructokinase; [7]FBA: Fructose-bisphosphate aldolase; [8]PGM: Phosphoglycerate Mutase; [9]ENO: Enolase; [10]PykA: Pyruvate kinase A; [11]LDH: Lactate dehydrogenase *Presence of glucose detected in maltose standard (Sigma-Aldrich).

Test of reverse direction gluconeogenic reactions starting with 3-PG. To determine if the reverse reaction from 3-PG to dihydroxyacetone phosphate was viable, the consumption of NADH was monitored over 48 hrs. The following enzymes were assembled with increasing amounts of QDs: PGK, GAPDH, and TPI. Stock solutions of enzyme and QD were made in buffer containing: 50 mM Tris HCl (pH = 8), 10 mM NaCl, and 12 mM $MgCl_2$. Tris buffer was utilized as a more optimal buffer for this reaction set. Enzymes were added to QDs in a similar manner as described previously: PGK followed by GAPDH followed by TPI. Reaction mixtures were allowed to assemble at 4ºC for at least 1 hr. To a 384 well plate, 25 µL of enzyme-QD construct was added followed by 25 µL of substrate solution. The final concentration of QDs ranged from 0 to 12 nM while the final concentration of PGK was approximately 9 nM, 30 nM GAPDH, and 1 nM TPI. The final concentration of the components of the substrate solution were as follows: 10 mM 3-phosphoglycerate, 100 µM ADP, 3 mM dibasic/monobasic phosphate, 2 mM NADH, and 40 mM polyphosphate in 50 mM Tris HCl buffer. The plate was immediately placed in a Tecan Sapphire plate reader and a kinetic program was started as described previously however a time point was taken every 2.5 minutes for 1000 kinetic cycles. Data was converted to NADH concentration as described previously.

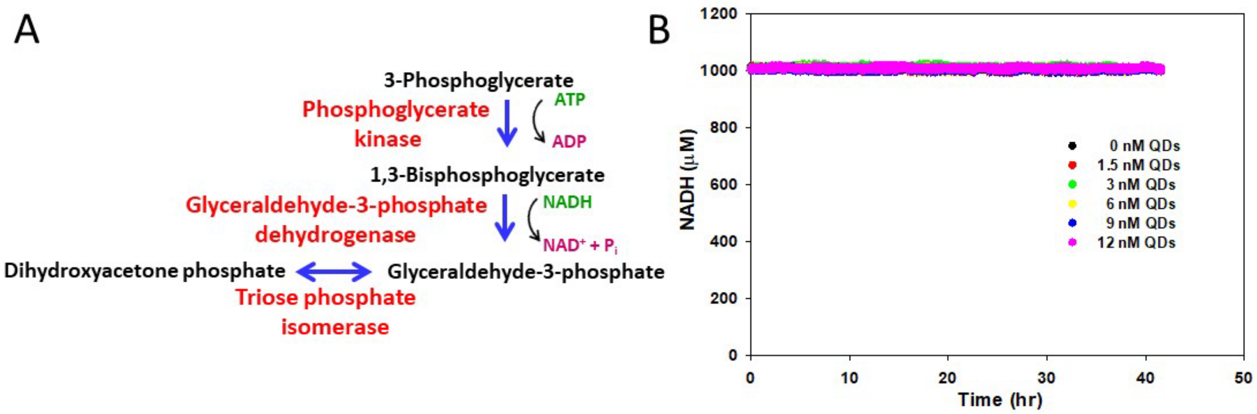

Supplementary Figure 49. Gluconeogenic reaction starting with 3-PG. (A) Reaction scheme showing coupled reactions utilized. **(B)** Experimental results where a fixed concentration of the 3 enzymes was assembled with increasing concentrations of QDs as indicated and assayed. There is no NADH conversion observed.

Assay shaking and testing different enzyme assembly orders with the QDs.

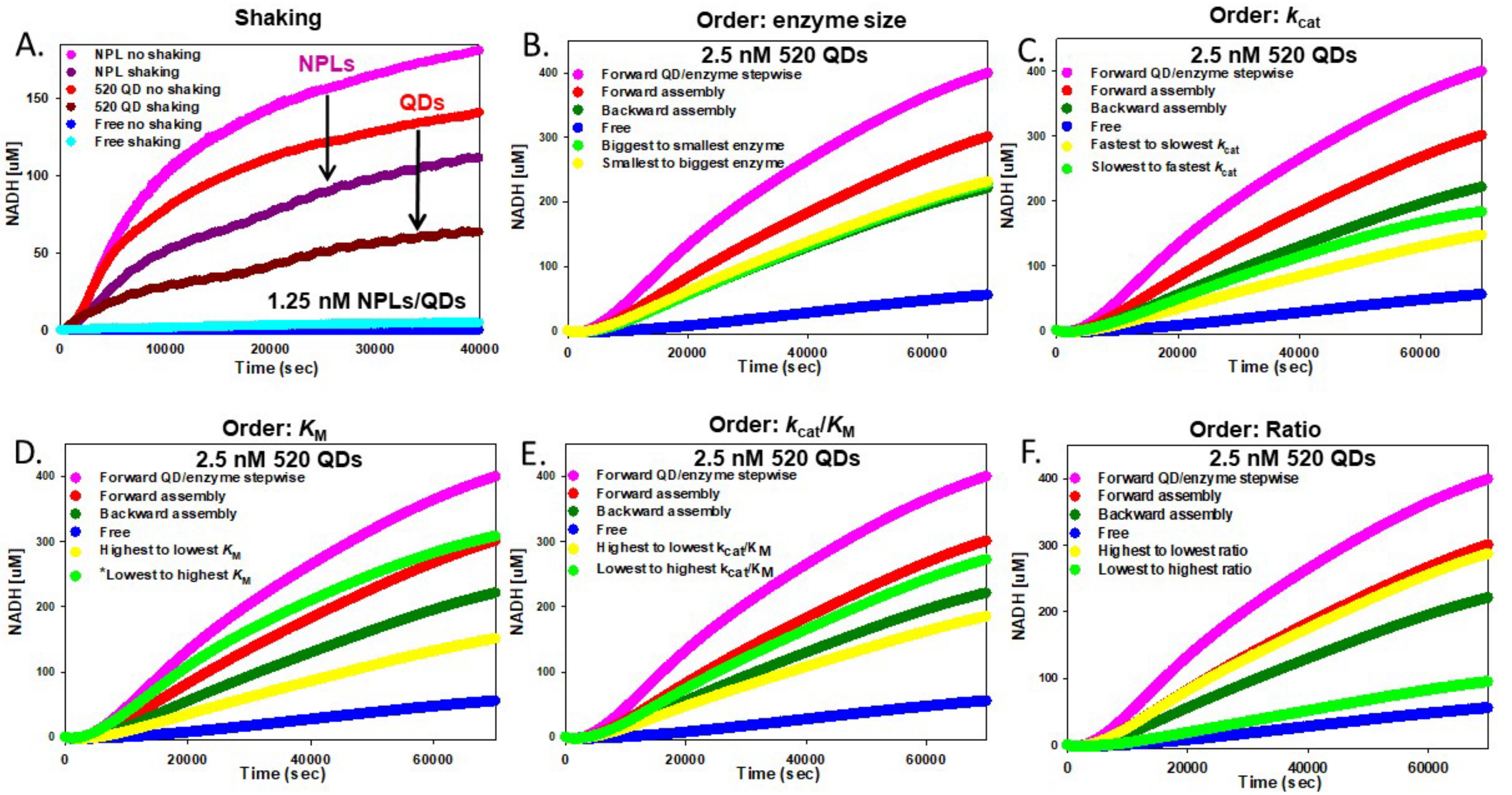

Supplementary Figure 50. Assay shaking and assembly order. (A) NPLs or 520 QDs assembled with the 7E system at Opt 2 ratios and assayed with no shaking or continuous shaking of the sample plate. **(B-F)** Results from a series of assays carried out where the order of enzyme assembly was varied. Within each of these panels the Forward QD/enzyme stepwise, Forward assembly, Backward assembly, and Free data plots taken from Figure 4F are shown for comparison. To these data are added assay results where clusters were assembled with enzymes in the following order: **(B)** enzyme size (biggest to smallest and smallest to biggest), **(C)** k_{cat} (fastest to slowest and slowest to fastest), **(D)** K_M (highest to lowest and lowest to highest), **(E)** k_{cat}/K_M (highest to lowest and lowest to highest), and **(F)** enzyme ratio to QD present (highest to lowest and lowest to highest),

Forward, backward, and batch assembly results (Manuscript Figure 5E, F and Supplementary Figure 50). We presume that in the Forward approach, the enzymes have a higher probability of being in a somewhat more sequential order from the inside of the cluster to the outside while the Backward assembly approach has the converse effect. In both configurations, the Glk sees the same initial concentration of glucose but in the Forward assembly, the initially formed glucose-6-phosphate (G6P) intermediate has a high probability of being generated inside the cluster and being utilized by the next enzyme and so on rather than diffusing away to the bulk. The further stepwise assembly of **Figure 5F** may allow for a higher probability of more enzyme to be present in a cluster along with formation of potentially bigger clusters due to the stepwise ordering of enzyme and QD addition. This latter approach may also prevent a given enzyme from binding up all the available QD attachment space during its addition. In the Backward assembly, the Glk would be at the cluster periphery and this would allow more G6P intermediate to diffuse away to bulk. This supposition is supported by the results in manuscript **Figure 5C** where cluster presence protected glucose from the activity of free glucose oxidase. For this to occur, the enzymes would have to be inside the cluster. The ordered assembly format (Forward) may also help increase the probability that almost every cluster has at least several copies of the first enzymes in the 7E or 4E cascade present whereas with batch assembly this would be more random and thus less efficient as observed experimentally.

We further tested the effect of assembly order based on kinetic or enzyme size/ratio variables to see if these contributed to channeled catalytic flux. This included ordered assembly based on enzyme size (biggest to smallest and smallest to biggest), k_{cat} (fastest to slowest and slowest to fastest), K_M (highest to lowest and lowest to highest), k_{cat}/K_M (highest to lowest and lowest to highest) and enzyme ratio to NP (highest to lowest and lowest to highest). Ordering of k_{cat} and K_M was meant to look for any potential dominating effect from rate and selectivity within the cluster, respectively, while k_{cat}/K_M similarly looked at efficiency. As shown in **Supplementary Figure 50**, ordering based on enzyme size **(Supplementary Figure 50B)** and k_{cat} **(Supplementary Figure 50C)**, in forward and backward directions did not produce any substantial improvement. We do note that assembly based on and K_M **(Supplementary Figure 50D)** and k_{cat}/K_M **(Supplementary Figure 50E)** (highest to lowest) did yield progress curves that were similar to the forward assembly in magnitude. We also note that the enzyme order for these samples and the forward assembly are almost identical suggesting this as the reason for the apparent improvement

– we are simply recapitulating the forward assembly. The assembly based on ratio (highest to lowest) **(Supplementary Figure 50F)** also matches forward assembly and the reason behind this are intriguing but harder to explain. We speculate that this allows for more of the lower activity enzymes to be present in the cluster thus contributing to improving flux. The plots in **Supplementary Figure 50B-E** reflect intrinsic properties of the enzymes and these results show that these properties cannot fully account for the observed enhancement relative to the order. Overall, based on these crude assays, we conclude that enzyme sequentiality seems to be more significant of a factor for kinetic flux in these clusters than ratio, size or kinetic parameters. Another hypothesis is that enzyme addition order directly effects cluster size macroscopically or microscopically and that this, in turn, contributes towards the improved kinetic flux.

We do note in the literature some debate on whether enzyme proximity is more influential than sequentiality or order for channeling. The current result runs somewhat counter to suggestions that enzyme order should not contribute significantly to channeling and that proximity is paramount.[44-48] Indeed, Wingreen and co. stresses "the importance of achieving maximum density but the relative unimportance of internal cluster organization" for their 2 enzyme system.[49] That may be true for 2 enzymes – but with multiple steps the order and the proximity that order would bring in a tight cluster may become more important for achieving efficient channeling. We do not delve further into this more complex issue here beyond this speculation.

Testing of other enzyme aggregated materials

Nickel-induced aggregation of enzymes. There is literature precedent for divalent cations including Ni^{2+} aggregating proteins together and especially those displaying terminal hexahistidine motifs.[50-55] Towards potentially exploring this route to create enzyme clusters, we tested the effect of increasing concentrations of Ni^{2+} on the 7E cascade to ascertain how this would affect baseline activity and kinetic flux through the system. The Ni^{2+} concentration ranged from 10 nM up to 50 μM with the lower boundary being similar to the amount of enzyme and NPs used in our assays. The 7E system was allowed to interact with and assemble to Ni^{2+} in a similar manner as described in pervious sections. Briefly, nine stock solutions were made with varying concentrations of nickel chloride hexahydrate, 0 to 100 μM, each containing the following: 55 nM GLK, 10 nM PGI, 90 nM FPK, 120 nM FBA, 10 nM TPI, 270 nM GPD, 75 nM PGK. The solutions were assembled in 250 mM HEPES buffer at pH = 8 and allowed to assemble overnight at 4ºC. The following morning, 25 μL aliquots were added to a 384-well plate to achieve quadruplicate replicates. To start the reaction, 25 μL of substrate solution was added to each well and consisted of: 30 mM $MgCl_2$, 15 mM ATP, 15 mM ADP, 20 mM glucose, 8 mM diphosphate/monophosphate solution, and 4.5 mM NAD^+ in 250 mM HEPES. The plate was immediately placed in a Tecan Spark plate reader and the absorbance at 340 nm was measured overtime for up to 18 hrs. The final concentration of all components in 250 mM HEPES buffer excluding Ni^{2+} are as follows: 27.5 nM GLK, 5 nM PGI, 45 nM FPK, 60 nM FBA, 5 nM TPI, 135 nM GPD, 37.5 nM PGK, 15 mM $MgCl_2$, 7.5 mM ATP, 7.5 mM ADP, 10 mM glucose, 4 mM diphosphate/monophosphate solution, and 2.25 mM NAD^+. Absorbance values were then converted to NADH concentration values employing Beer's law and a molar absorptivity of 6220 $M^{-1}cm^{-1}$. The rate of NADH formation was determined from the slope of the linear portions of each progress curve. As seen in **Supplementary Figure 51**, this resulted in a significant decrease in the overall catalytic rate and flux through the system. Indeed, by 50 μM Ni^{2+}, the rate is down more than 10-fold. The decrease in flux presumably occurs by the metal poisoning and perturbation of the active sites of the different enzymes present in an unpredictable manner. Moreover, the Ni^{2+} would affect the overall rate of flux at concentrations required to form protein aggregates or clusters and thereby negate our ability to numerically simulate the systems. Since this approach negatively affects one of our key precepts, namely by modifying enzyme activity in a manner that cannot be accounted for, it was not pursued further.

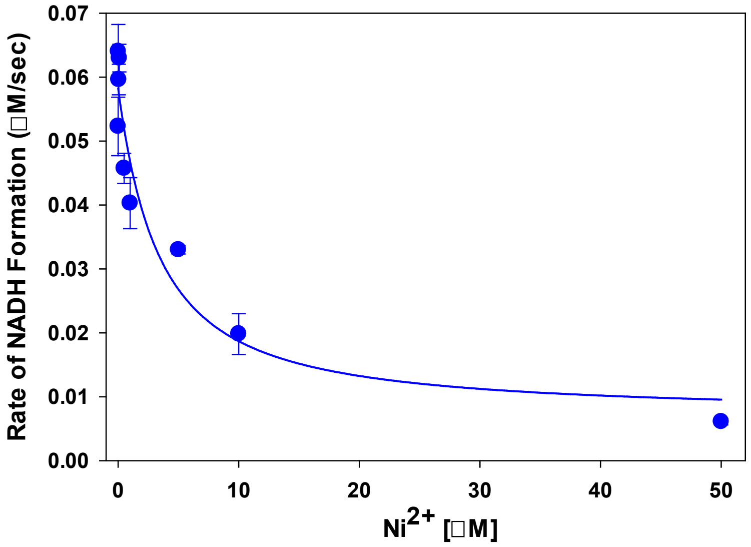

Supplementary Figure 51. Testing of 7E system *versus* increasing Ni^{2+} presence. A fixed concentration of the 7E system free in solution at Opt 2 ratios was assembled and tested against the indicated increasing concentrations of Ni^{2+}. Corresponding initial rates of NADH formation are shown as a function of Ni^{2+} concentration. Data shown is the mean from n = 3 independent experimental samples ± standard deviation.

Tannic acid-induced aggregation and cross-linking of enzymes. There is literature precedent for tannic acid (TA) in various forms and procedures being utilized for aggregating proteins together.[56, 57] Towards potentially exploring this route to create enzyme clusters, we tested the effect of increasing concentrations of TA on the 7E cascade to ascertain how this would affect baseline activity and kinetic flux through the system. The TA concentration ranged from 2 nM up to 1 μM with the lower boundary being similar to the amount of enzyme and NPs used in our assays. The 7E system was allowed to interact with and assemble to TA in a similar manner as described in pervious sections. Briefly, stock solutions were assembled with varying concentrations of TA each containing the following: 55 nM GLK, 10 nM PGI, 90 nM FPK, 120 nM FBA, 10 nM TPI, 270 nM GPD, 75 nM PGK. The solutions were assembled in 250 mM HEPES buffer at pH = 8 and allowed to assemble overnight at 4ºC. Assays were implemented in the same manner as that described above for the Ni^{2+} aggregation assay. As seen in **Supplementary Figure 51**, this resulted in a significant decrease in the overall catalytic rate and flux through the system. Indeed, by 40 nM TA, the rate is down almost half. The decrease in flux presumably occurs by perturbation of the active sites of the different enzymes present in an unpredictable manner. This presumably occurs since the TA interacts with the enzymes through hydrophobic, hydrogen and electrostatic interactions. These can induce protein unfolding as previously observed with lysozyme.[58] Similar to the above, this approach negatively affects one of our key precepts, namely by modifying enzyme activity in a manner that cannot be accounted for, thus it was not pursued further.

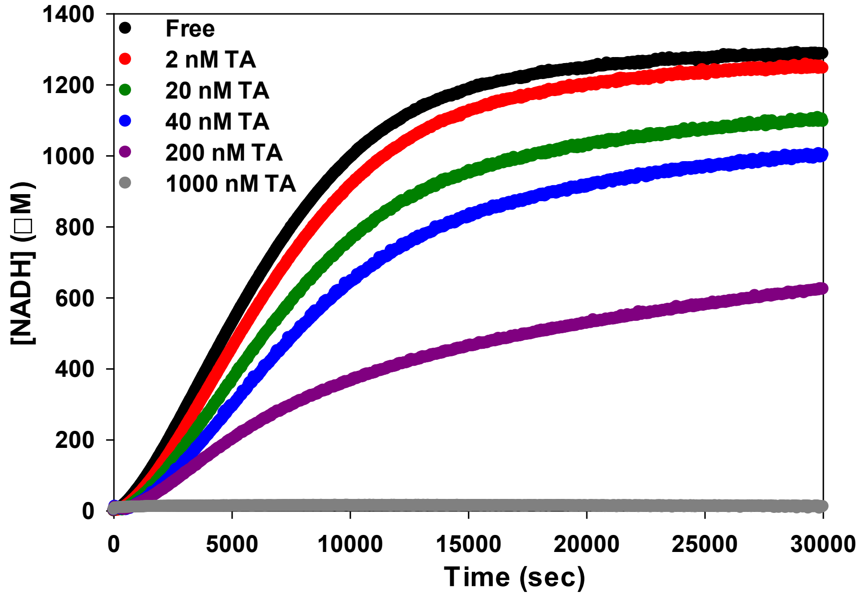

Supplementary Figure 52. Testing of 7E system *versus* increasing tannic acid (TA) presence. A fixed concentration of the 7E system free in solution at Opt 2 ratios was assembled and tested against the indicated increasing concentrations of TA. Corresponding NADH formation is shown as a function of TA concentration.

Glutaraldehyde-induced aggregation and cross-linking of enzymes. There is a strong literature precedent for glutaraldehyde (GA) being utilized for aggregating proteins together to form crosslinked enzyme aggregates (CLEAs).[59, 60] Towards potentially exploring this chemical route to create enzyme clusters, we tested the effect of increasing concentrations of GA on the 7E cascade to ascertain how this would affect baseline activity and kinetic flux through the system. The GA concentration ranged from 10 nM up to 1 μM with the lower boundary being similar to the amount of enzyme and NPs used in our assays. The 7E system was allowed to interact with and assemble to GA in a similar manner as described in previous sections. Assays were implemented in the same manner as that described above for the Ni^{2+} aggregation and TA assays. As seen in **Supplementary Figure 52**, this resulted in a significant decrease in the overall catalytic rate and flux through the system. Indeed, by 500 nM GA, the rate is down almost 25% and by 1 μM the system is no longer functional. The decrease in flux presumably occurs by perturbation of the active sites of the different enzymes present in an unpredictable manner along with the crosslinking. GA chemically reacts with proteins in several different ways by condensing amines including *via* Mannich reactions, and/or Michael addition and reductive amination. Similar to that described above, this approach negatively affects one of our key precepts, namely by modifying enzyme activity in a manner that cannot be accounted for, thus it was not pursued further.

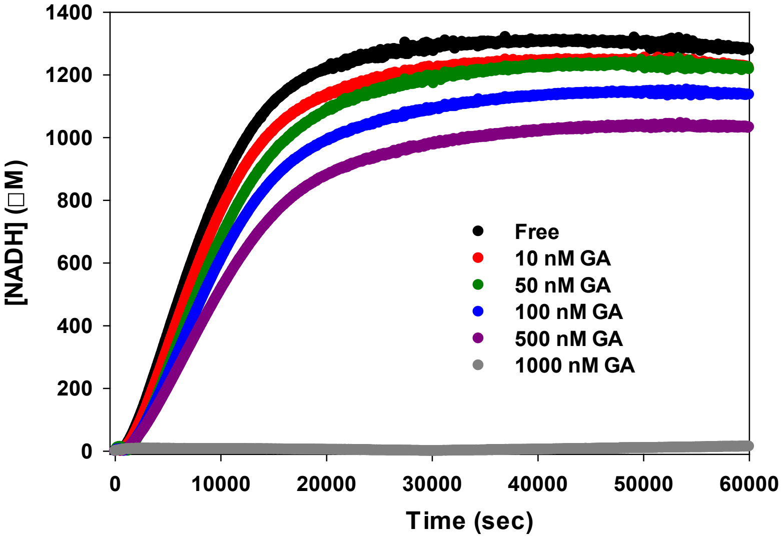

Supplementary Figure 53. Testing of 7E system *versus* increasing glutaraldehyde (GA) presence. A fixed concentration of the 7E system free in solution at Opt 2 ratios was assembled and tested against the indicated increasing concentrations of GA. Corresponding NADH formation is shown as a function of TA concentration to estimate the affect.

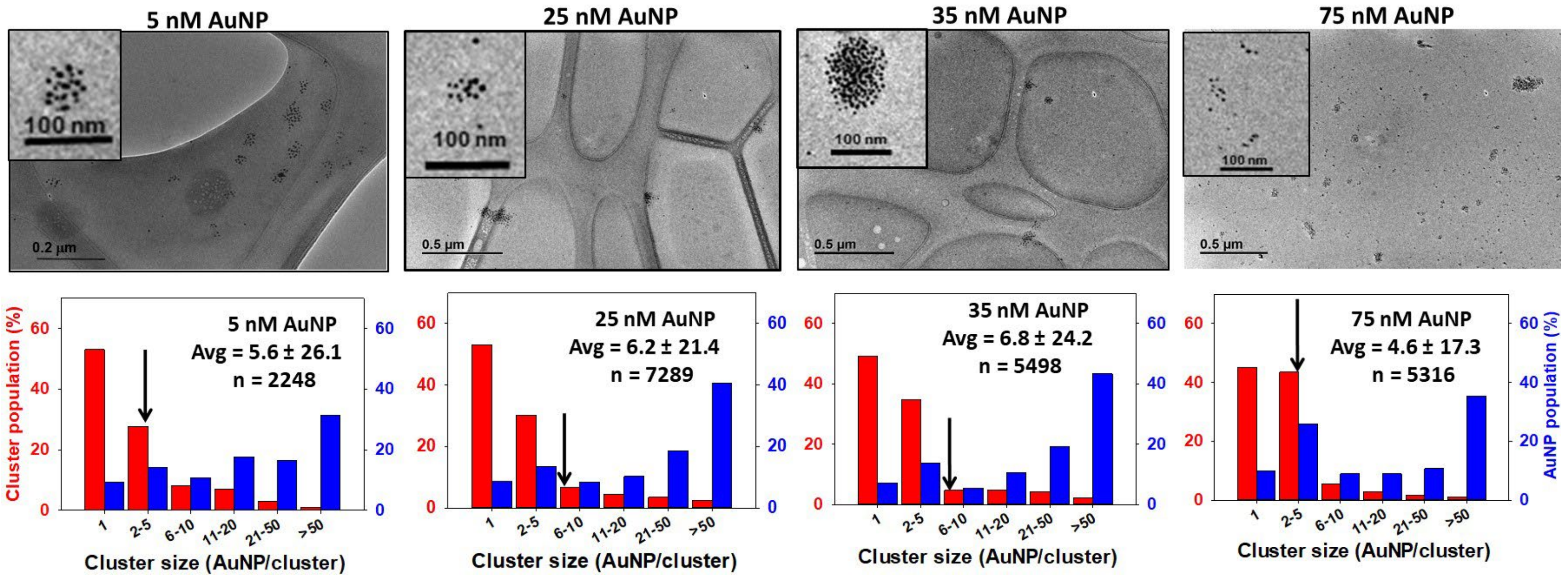

Supplementary Figure 54. TEM characterization of 5 nm diameter AuNP-enzyme clusters from Manuscript Figure 7A-C. Top - Representative TEMs of AuNPs assembled with the 7 enzyme system (Opt 2 ratios with enzyme concentration fixed (Glk 5.5, PGI 1, PFK 9, FBA 12, TPI 1, GPD 27, PGK 7.5 nM) as assembled with the indicated concentrations of AuNP. Bottom - bar plots with corresponding cluster analysis and average cluster size (black arrow) and number of AuNPs counted given. The high-resolution insets in the TEMs show a representative AuNP cluster from that sample – but not one that is necessarily reflective of the average size.

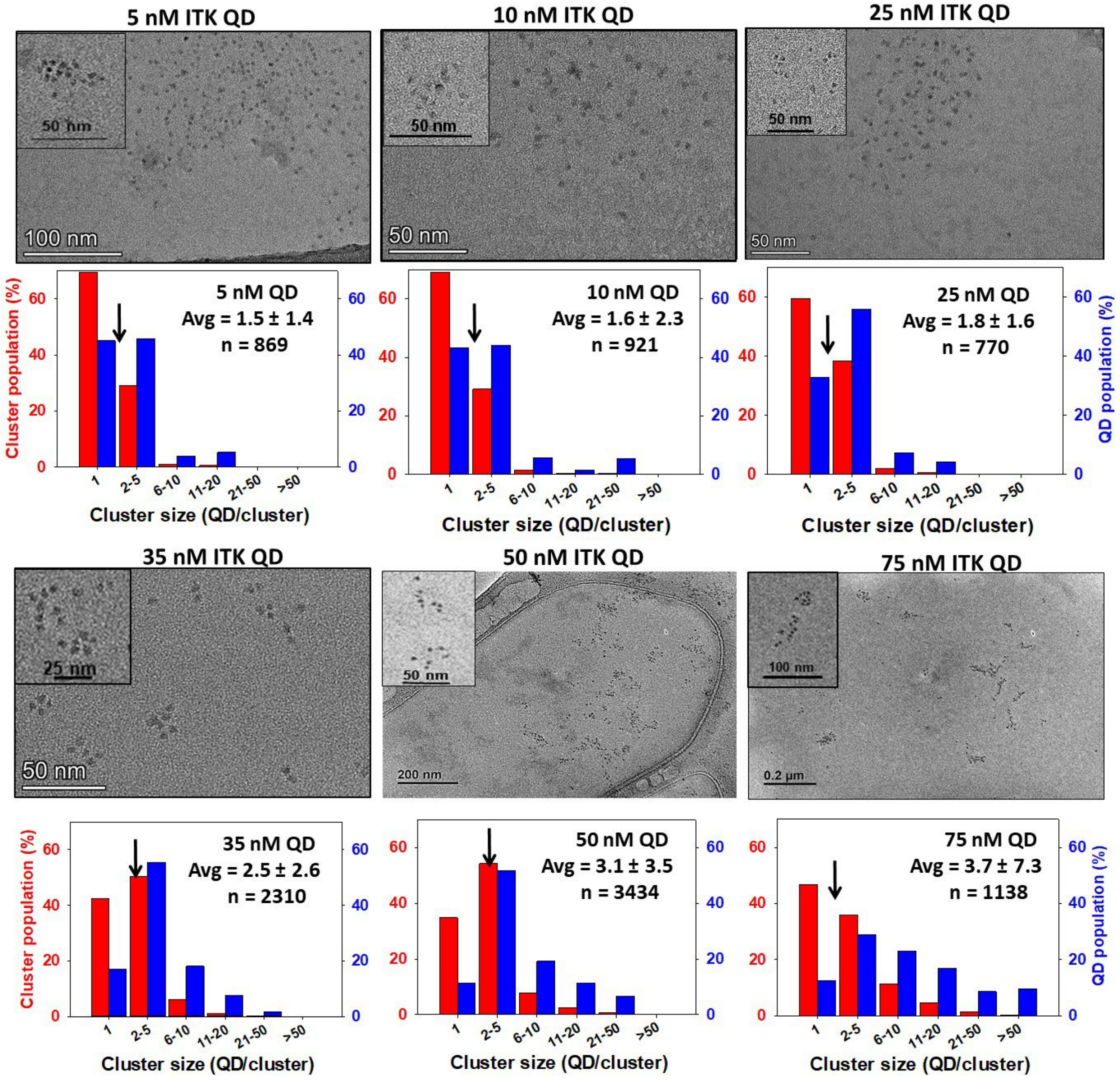

Supplementary Figure 55. TEM characterization of 525 nm emitting ITK carboxyl QD-enzyme clusters from Manuscript Figure 7D-F. Top - Representative TEMs of QDs assembled with the 7 enzyme system (Opt 2 ratios with enzyme concentration fixed (Glk 5.5, PGI 1, PFK 9, FBA 12, TPI 1, GPD 27, PGK 7.5 nM) as assembled with the indicated concentrations of QD. Bottom - bar plots with corresponding cluster analysis and average cluster size (black arrow) and number of QDs counted given. The high-resolution insets in the TEMs show representative QD cluster(s) from that sample – but not those that are necessarily reflective of the average size.

Supplementary References

1. Diaz, S.A., Breger, J.C. & Medintz, I.L. Monitoring enzymatic proteolysis using either enzyme- or substrate-bioconjugated quantum dots. *Methods in Enzymology* **571**, 19-54. (2016).
2. Breger, J.C. et al. Understanding how nanoparticle attachment enhances phosphotriesterase kinetic efficiency. *ACS Nano* **9**, 8491-8503 (2015).
3. Breger, J.C. et al. Assembling high activity phosphotriesterase composites using hybrid nanoparticle peptide-DNA scaffolded architectures. *Nano Futures* **1**, 011002 (2017).
4. Breger, J.C. et al. Nanoparticle size influences localized enzymatic enhancement-A case study with phosphotriesterase. *Bioconjugate Chemistry* **30**, 2060-2074 (2019).
5. Breger, J.C. et al. Quantum dot display enhances activity of a phosphotriesterase trimer. *Chemical Communications* **51**, 6403-6406 (2015).
6. Boeneman, K. et al. Selecting improved peptidyl motifs for cytosolic delivery of disparate protein and nanoparticle materials. *ACS Nano* **7**, 3778-3796 (2013).
7. Sapsford, K.E. et al. Monitoring botulinum neurotoxin a activity with peptide-functionalized quantum dot resonance energy transfer sensors. *ACS Nano* **5**, 2687-2699 (2011).
8. Breger, J.C. et al. Nanoparticle cellular uptake by dendritic wedge peptides: achieving single peptide facilitated delivery. *Nanoscale* **9**, 10447-10464 (2017).
9. Prasuhn, D.E. et al. Polyvalent display and packing of peptides and proteins on semiconductor quantum dots: Predicted versus experimental results. *Small* **6**, 555-564 (2010).
10. Prasuhn, D.E., Susumu, K. & Medintz, I.L. Multivalent conjugation of peptides, proteins, and DNA to semiconductor quantum dots. *Methods in Molecular Biology* **726**, 95-110 (2011).
11. Green, C.M. et al. Direct and efficient conjugation of quantum dots to DNA nanostructures with peptide-PNA. *ACS Nano* **15**, 9101-9110 (2021).
12. Berti, L., D'Agostino, P.S., Boeneman, K. & Medintz, I.L. Improved peptidyl linkers for self-assembly of semiconductor quantum dot bioconjugates. *Nano Research* **2**, 121-129 (2009).
13. Gemmill, K.B. et al. Optimizing protein coordination to quantum dots with designer peptidyl linkers. *Bioconjugate Chemistry* **24**, 269-281 (2013).
14. Gemmill, K.B. et al. Evaluation of diverse peptidyl motifs for cellular delivery of semiconductor quantum dots. *Analytical and Bioanalytical Chemistry* **405**, 6145-6154 (2013).
15. Goldman, E.R. et al. A hybrid quantum dot-antibody fragment fluorescence resonance energy transfer-based TNT sensor. *Journal of the American Chemical Society* **127**, 6744-6751 (2005).
16. Sapsford, K.E. et al. Kinetics of metal-affinity driven self-assembly between proteins or peptides and CdSe-ZnS quantum dots. *Journal of Physical Chemistry C* **111**, 11528-11538 (2007).
17. Vranish, J.N. et al. Enhancing coupled enzymatic activity by colocalization on nanoparticle surfaces: Kinetic evidence for directed channeling of intermediates. *ACS Nano* **12**, 7911-7926 (2018).

18. Vranish, J.N., Ancona, M.G., Walper, S.A. & Medintz, I.L. Pursuing the promise of enzymatic enhancement with nanoparticle assemblies. *Langmuir* **34**, 2901-2925 (2018).
19. Hers, H.G. & Hue, L. Gluconeogenesis and related aspects of glycolysis. *Annual Review of Biochemistry* **52**, 617-653 (1983).
20. Scrutton, M.C. & Utter, M.F. Regulation of glycolysis and gluconeogenesis in animal tissues. *Annual Review of Biochemistry* **37**, 249-302 (1968).
21. Klein, W.P. et al. Enhanced catalysis from multienzyme cascades assembled on a DNA origami triangle. *ACS Nano* **13**, 13677-13689 (2019).
22. Rios, L.M. & Sahinidis, N.V. Derivative-free optimization: a review of algorithms and comparison of software implementations. *Journal of Global Optimization* **56**, 1247-1293 (2013).
23. Shahriari, B., Swersky, K., Wang, Z.Y., Adams, R.P. & de Freitas, N. Taking the human out of the loop: A review of Bayesian optimization. *Proceedings of the IEEE* **104**, 148-175 (2016).
24. Acerbi, L. & Ma, W.J. in 31st Annual conference on neural information processing systems (NIPS), Vol. 30 (Long Beach, CA; 2017).
25. Beber, M.E. et al. eQuilibrator 3.0: a database solution for thermodynamic constant estimation. *Nucleic Acids Research* **50**, D603-D609 (2022).
26. Stellwagen, E., Prantner, J.D. & Stellwagen, N.C. Do zwitterions contribute to the ionic strength of a solution? *Analytical Biochemistry* **373**, 407-409 (2008).
27. Breger, J.C. et al. Quantum dot lipase biosensor utilizing a custom-synthesized peptidyl-ester substrate. *ACS Sensors* **5**, 1295-1304 (2020).
28. Diaz, S.A. et al. Quantum dots as Forster resonance energy transfer acceptors of lanthanides in time-resolved bioassays. *ACS Applied Nano Materials* **1**, 3006-3014 (2018).
29. Gemmill, K.B. et al. Examining the polyproline nanoscopic ruler in the context of quantum dots. *Chemistry of Materials* **27**, 6222-6237 (2015).
30. Blanco-Canosa, J.B. et al. Recent progress in the bioconjugation of quantum dots. *Coordination Chemistry Reviews* **263**, 101-137 (2014).
31. Brown, C.W. et al. Kinetic enhancement of the diffusion-limited enzyme beta-galactosidase when displayed with quantum dots. *RSC Advances* **5**, 93089-93094 (2015).
32. Claussen, J.C. et al. Probing the enzymatic activity of alkaline phosphatase within quantum dot bioconjugates. *Journal of Physical Chemistry C* **119**, 2208-2221 (2015).
33. Mathur, D. & Medintz, I.L. Analyzing DNA nanotechnology: A call to arms for the analytical chemistry community. *Analytical Chemistry* **89**, 2646-2663 (2017).
34. Oh, E. et al. PEGylated luminescent gold nanoclusters: Synthesis, characterization, bioconjugation, and application to one- and two-photon cellular imaging. *Particle & Particle Systems Characterization* **30**, 453-466 (2013).
35. Susumu, K. et al. Purple-, blue-, and green-emitting multishell alloyed quantum dots: Synthesis, characterization, and application for ratiometric extracellular pH sensing. *Chemistry of Materials* **29**, 7330-7344 (2017).
36. Susumu, K. et al. A new family of pyridine-appended multidentate polymers as hydrophilic surface ligands for preparing stable biocompatible quantum dots. *Chemistry of Materials* **26**, 5327-5344 (2014).
37. Wang, Z. et al. Melanin produced by the fast-growing marine bacterium Vibrio natriegens through heterologous biosynthesis: Characterization and Application. *Applied and Environmental Microbiology* **86**, 5 e02749-19 (2020).

38. Hildebrandt, N. et al. Energy transfer with semiconductor quantum dot bioconjugates: A versatile platform for biosensing, energy harvesting, and other developing applications. *Chemical Reviews* **117**, 536-711 (2017).
39. Algar, W.R., Hildebrandt, N., Vogel, S.S. & Medintz, I.L. FRET as a biomolecular research tool-understanding its potential while avoiding pitfalls. *Nature Methods* **16**, 815-829 (2019).
40. Medintz, I.L. & Hildebrandt, N. (eds.) FRET-Förster Resonance Energy Transfer: From Theory to Applications. (John Wiley & Sons, Wenheim Germany; 2013).
41. Zijlstra, N., Blum, C., Segers-Nolten, I.M.J., Claessens, M.M.A.E. & Subramaniam, V. Molecular composition of sub-stoichiometrically labeled α-synuclein oligomers determined by single-molecule photobleaching. *Angewandte Chemie Int. Ed.* **51**, 8821-8824 (2012).
42. Sander, L.M. Diffusion-limited aggregation: a kinetic critical phenomenon? *Contemporary Physics* **41**, 203-218 (2000).
43. Witten, T.A. & Sander, L.M. Diffusion-limited aggregation, A kinetic critical phenomenon. *Physical Review Letters* **47**, 1400-1403 (1981).
44. Idan, O. & Hess, H. Engineering enzymatic cascades on nanoscale scaffolds. *Current Opinion in Biotechnology* **24**, 606-611 (2013).
45. Idan, O. & Hess, H. Origins of activity enhancement in enzyme cascades on scaffolds. *ACS Nano* **7**, 8658-8665 (2013).
46. Kuzmak, A., Carmali, S., von Lieres, E., Russell, A.J. & Kondrat, S. Can enzyme proximity accelerate cascade reactions? *Scientific Reports* **9**, 455 (2019).
47. Wheeldon, I. et al. Substrate channelling as an approach to cascade reactions. *Nature Chemistry* **8**, 299-309 (2016).
48. Sweetlove, L.J. & Fernie, A.R. The role of dynamic enzyme assemblies and substrate channelling in metabolic regulation. *Nature Communications* **9**, 2136 (2018).
49. Castellana, M. et al. Enzyme clustering accelerates processing of intermediates through metabolic channeling. *Nature Biotechnology* **32**, 1011-1018 (2014).
50. Friedberg, F. Effects of metal binding on protein structure. *Quarterly Reviews of Biophysics* **7**, 1-33 (1974).
51. Hedberg, Y.S. et al. Synergistic effects of metal-induced aggregation of human serum albumin. *Colloids and Surfaces B-Biointerfaces* **173**, 751-758 (2019).
52. Kozlowski, H., Potocki, S., Remelli, M., Rowinska-Zyrek, M. & Valensin, D. Specific metal ion binding sites in unstructured regions of proteins. *Coordination Chemistry Reviews* **257**, 2625-2638 (2013).
53. Sokolowska, M., Krezel, A., Dyba, M., Szewczuk, Z. & Bal, W. Short peptides are not reliable models of thermodynamic and kinetic properties of the N-terminal metal binding site in serum albumin. *European Journal of Biochemistry* **269**, 1323-1331 (2002).
54. Yang, J. & Black, J. Competitive-binding of chromium, cobalt and nickel to serum-proteins. *Biomaterials* **15**, 262-268 (1994).
55. Lopez-Laguna, H. et al. Divalent cations: A molecular glue for protein materials. *Trends in Biochemical Sciences* **45**, 992-1003 (2020).
56. Chen, C., Yang, H., Yang, X. & Ma, Q.H. Tannic acid: a crosslinker leading to versatile functional polymeric networks: a review. *RSC Advances* **12**, 7689-7711 (2022).

57. Nie, X.H., Zhao, L.M., Wang, N.N. & Meng, X.H. Phenolics-protein interaction involved in silver carp myofibrilliar protein films with hydrolysable and condensed tannins. *Lwt-Food Science and Technology* **81**, 258-264 (2017).
58. Su, J. et al. Effect of tannic acid on lysozyme activity through intermolecular noncovalent binding. *Journal of Agriculture and Food Research* **1**, 100004 (2019).
59. Barbosa, O. et al. Glutaraldehyde in bio-catalysts design: a useful crosslinker and a versatile tool in enzyme immobilization. *RSC Advances* **4**, 1583-1600 (2014).
60. Migneault, I., Dartiguenave, C., Bertrand, M.J. & Waldron, K.C. Glutaraldehyde: behavior in aqueous solution, reaction with proteins, and application to enzyme crosslinking. *Biotechniques* **37**, 790-802 (2004).
